# Supplementary figures and images for: High-throughput characterization of photocrosslinker-bearing ion channel variants to map residues critical for function and pharmacology
Source: PLoS Biol. 2021 Sep 7;19(9):e3001321. doi: 10.1371/journal.pbio.3001321 (PMC8448361; doi:10.1371/journal.pbio.3001321)

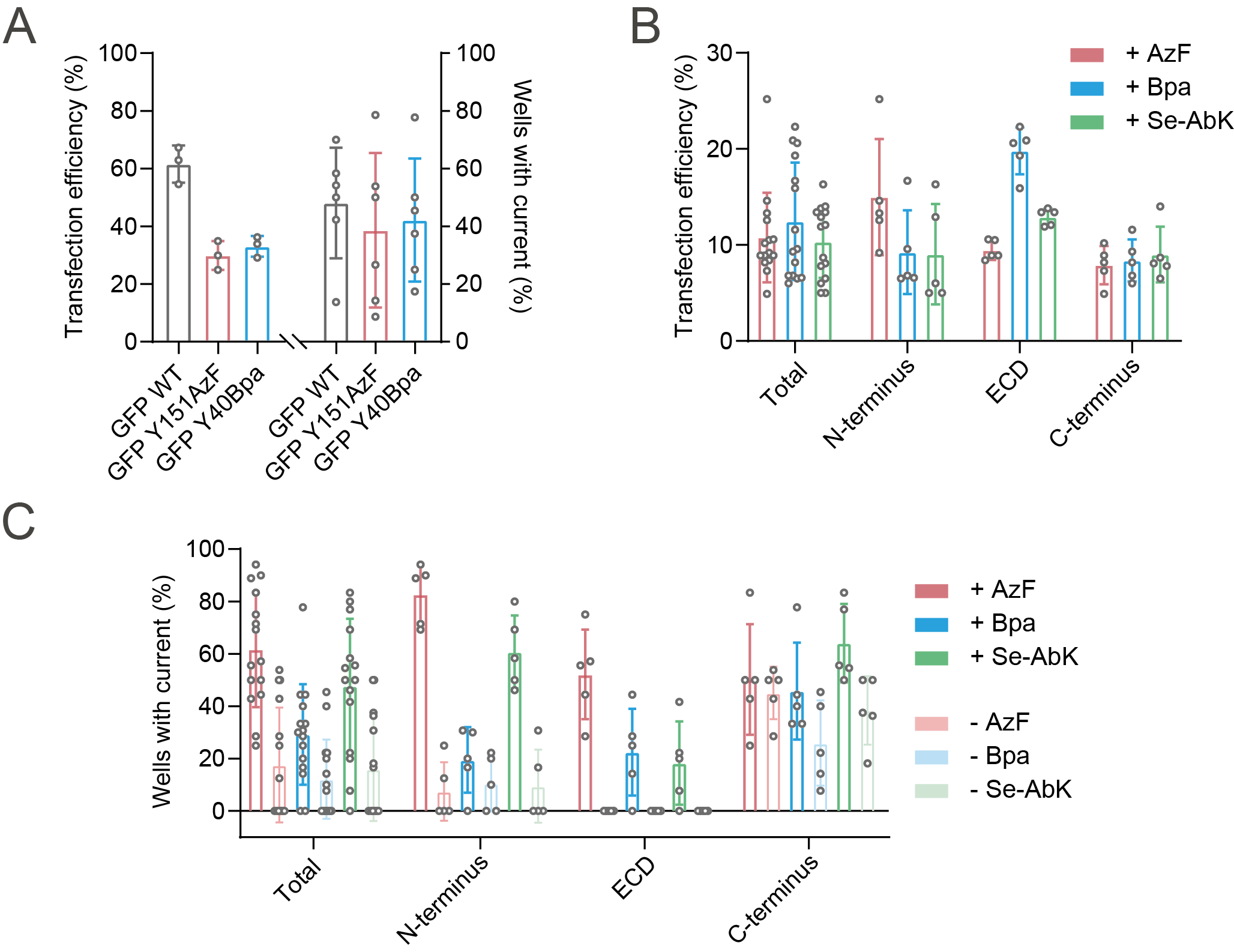

Supplement: S1 Fig — (A) Transfection efficiency and percentage of wells showing proton-gated currents (of wells harboring a patched cell with >100 MΩ seal) for hASIC1a WT co-transfected with WT GFP, GFP Y151AzF, or GFP Y40Bpa. While the transfection efficiency is reduced considerably from 61.6% to 29.9% and 33.1% upon co-transfection of GFP Y151AzF or GFP Y40Bpa, respectively, the decrease in mean current density is less pronounced from around 48% to 39% and 42%. This illustrates that nonsense suppression in GFP results in a decrease in apparent TE compared to WT and shows that both WT and TAG-containing GFP can be used as a reporter to enrich transfected cells for APC. (B) Transfection efficiency of 45 randomly selected hASIC1a variants assessed for ncAA incorporation in the N-terminus, ECD or carboxyl terminus. Of note, only cells grown in the presence of ncAA were FACS-sorted and assessed for transfection efficiency. (C) Percentage of wells showing proton-gated currents (of wells harboring a patched cell with >100 MΩ seal). Cells grown in the absence of ncAA (lighter shades in right panel) show currents in 0%–50% of the wells, depending on incorporation specificity, which varies in the different protein domains. All values shown as mean ± SD. The underlying data have been deposited at zenodo.org (https://doi.org/10.5281/zenodo.4906985; file 14). APC, automated patch clamp; ECD, extracellular domain; FACS, fluorescence-activated cell sorting; hASIC1a, human acid-sensing ion channel 1a; ncAA, noncanonical amino acid; SD, standard deviation; TE, transfection efficiency; WT, wild type. (TIF) [file pbio.3001321.s001.tif]

**S2 Fig.**


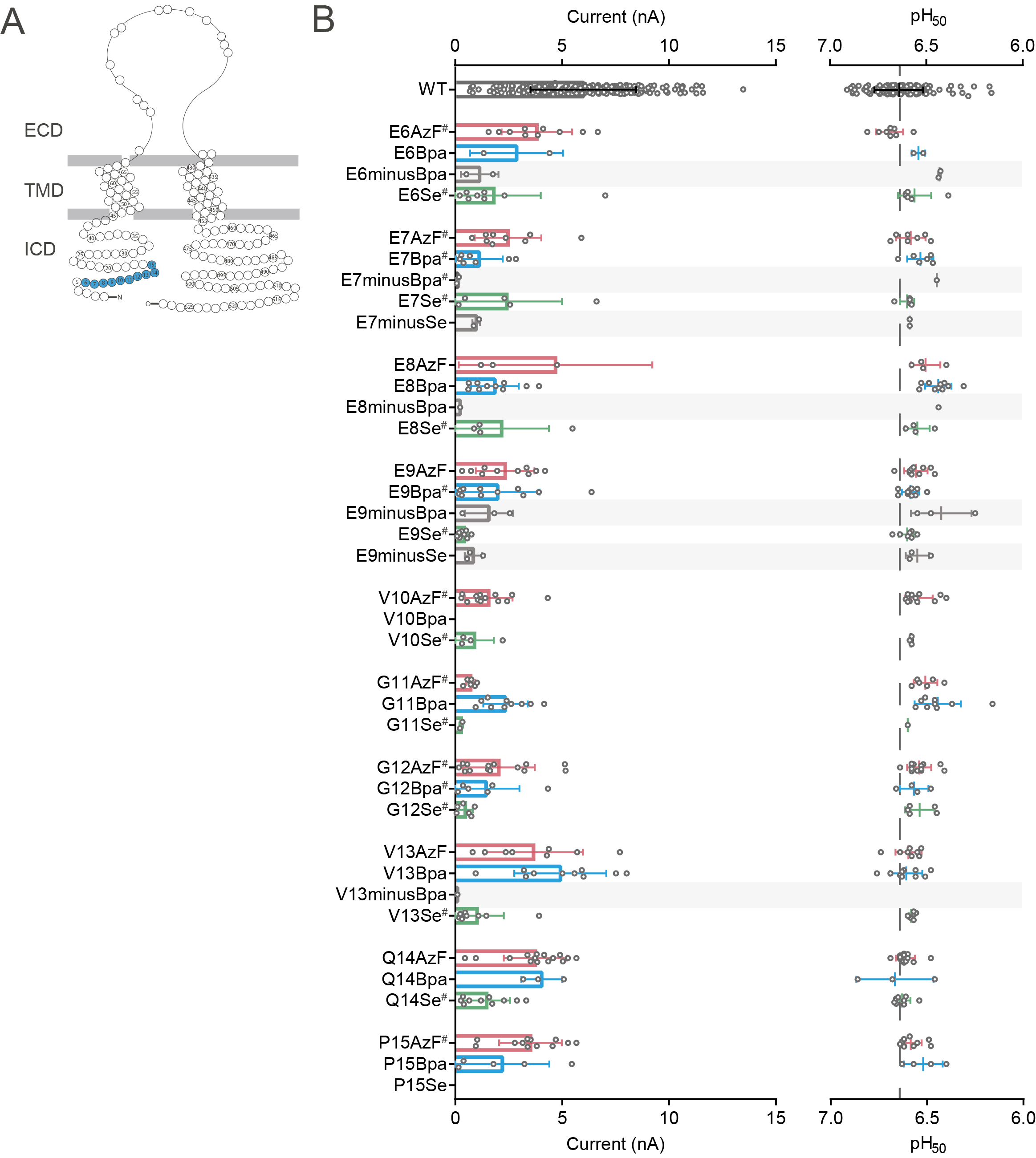


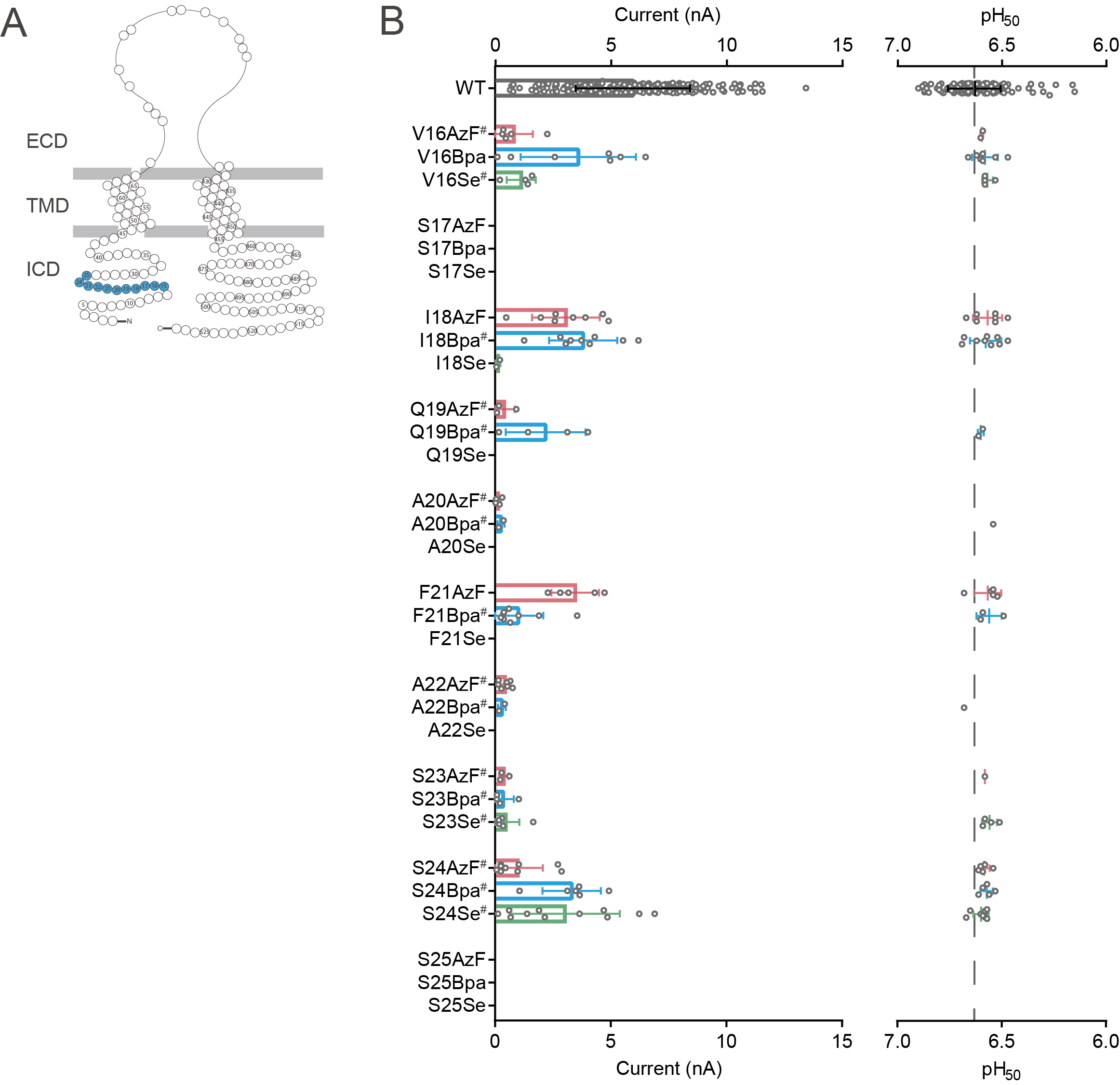


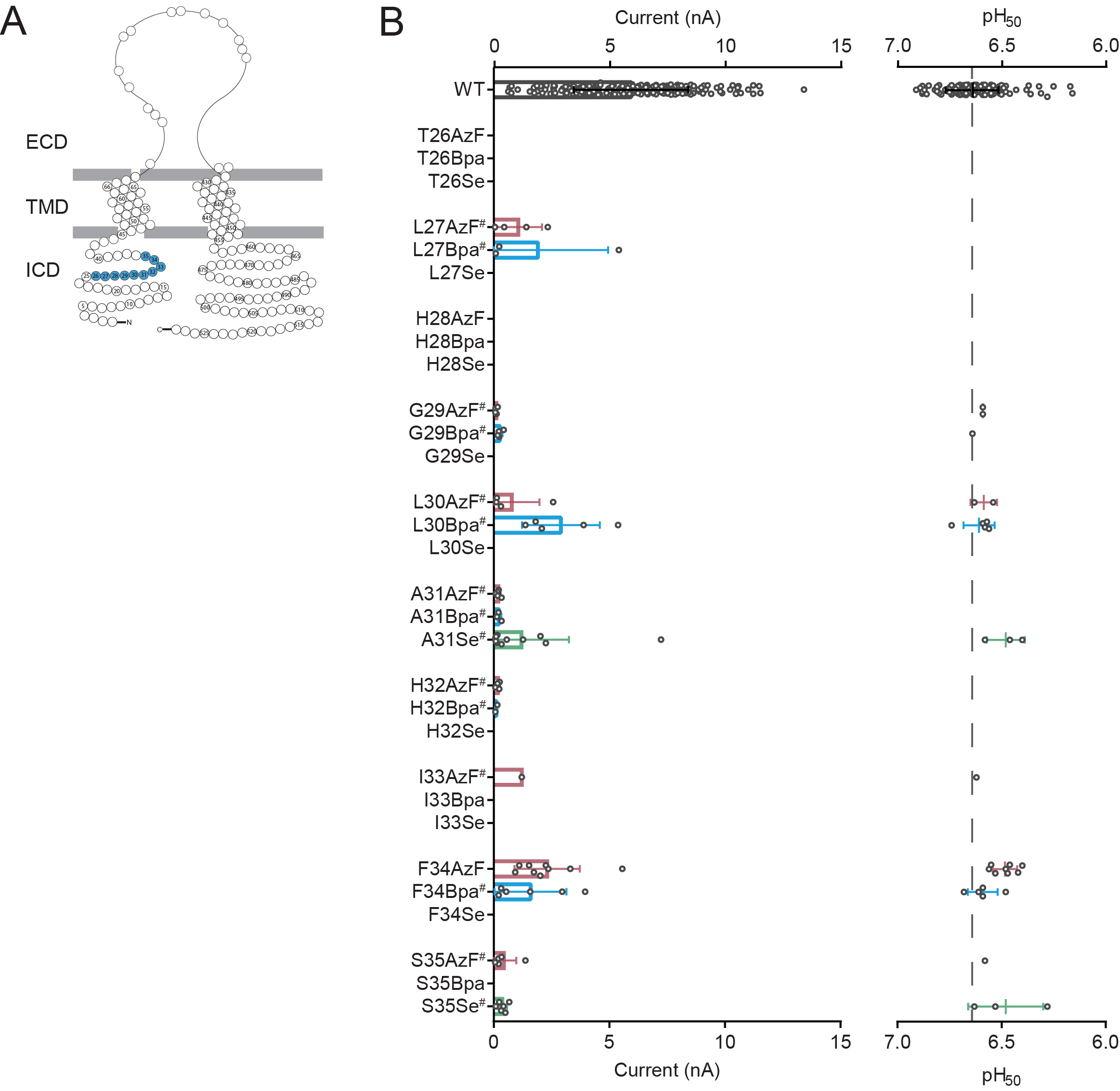


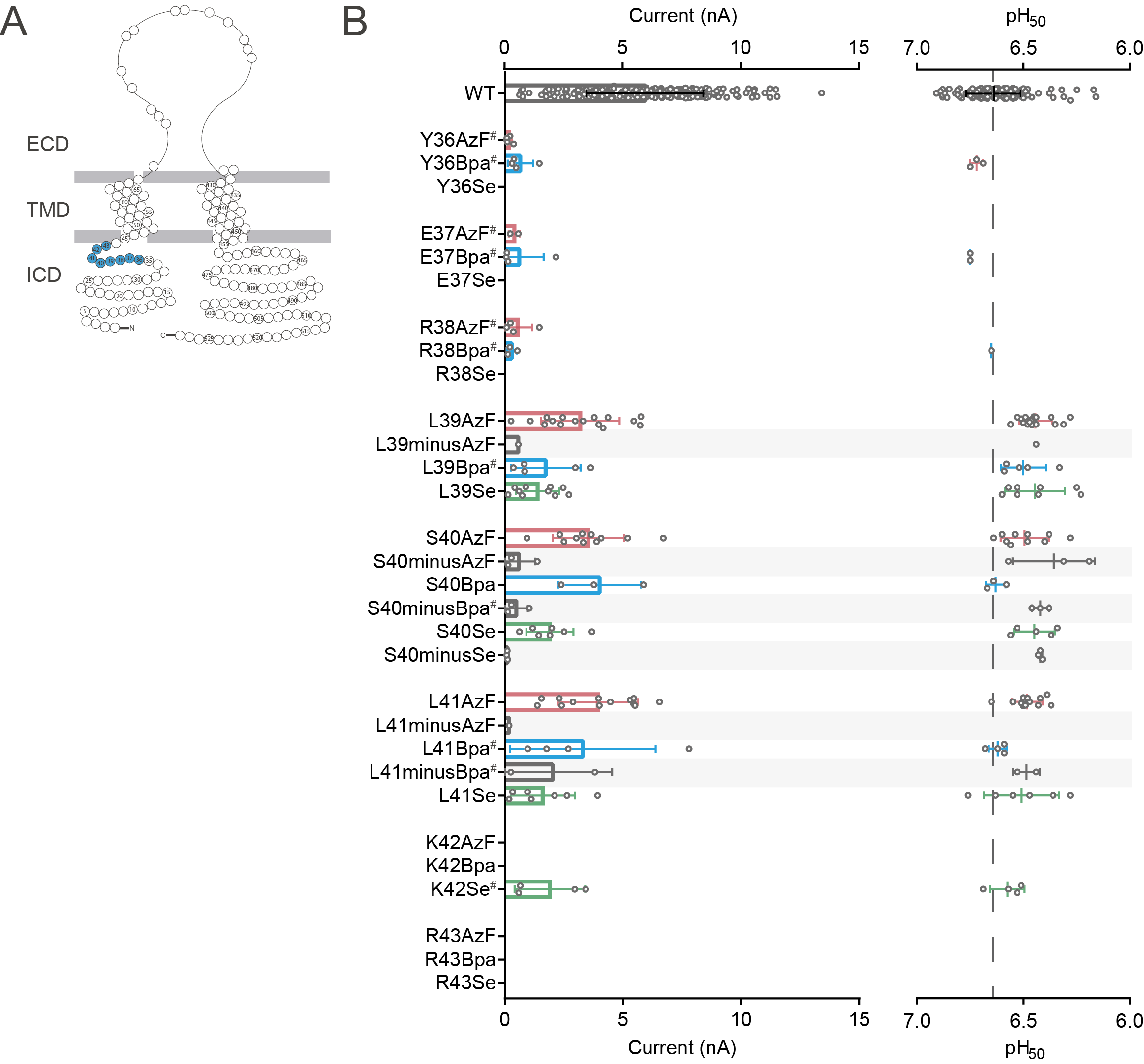

Supplement: S2 Fig — (A) Snake plots with tested positions marked in blue. (B) Dot plots comparing peak current sizes (left) and pH50 (right); bars indicate mean ± SD, and (#) marks >20% tachyphylaxis (see also S1 Table). For variants expressed in the absence of ncAAs that yielded currents, results are marked by underlying gray bars. The underlying data have been deposited at zenodo.org (https://doi.org/10.5281/zenodo.4906985; files 15–18). hASIC1a, human acid-sensing ion channel 1a; ncAA, noncanonical amino acid; SD, standard deviation; WT, wild type. (DOCX) [file pbio.3001321.s002.docx]

**S3 Fig.**

**
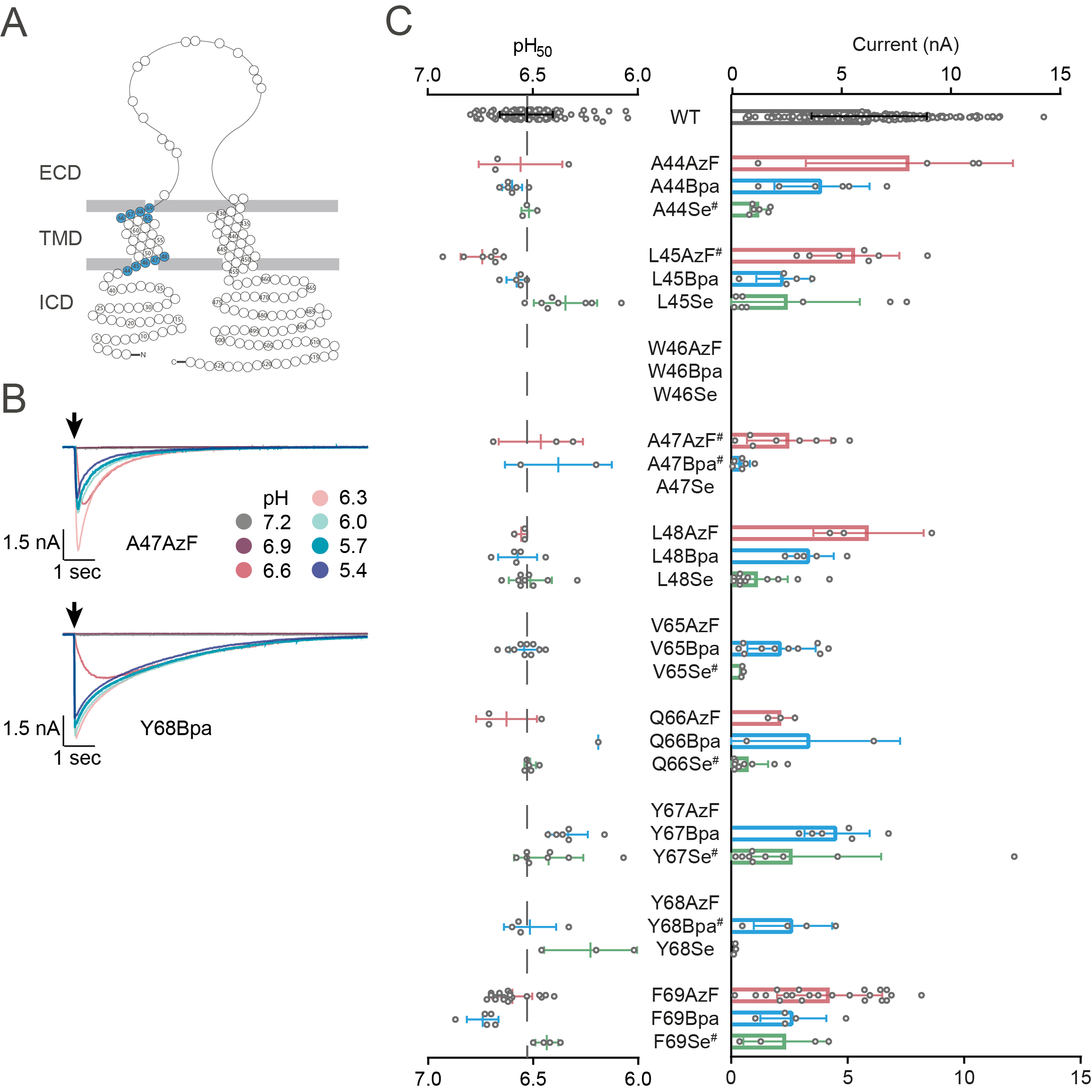
**

**
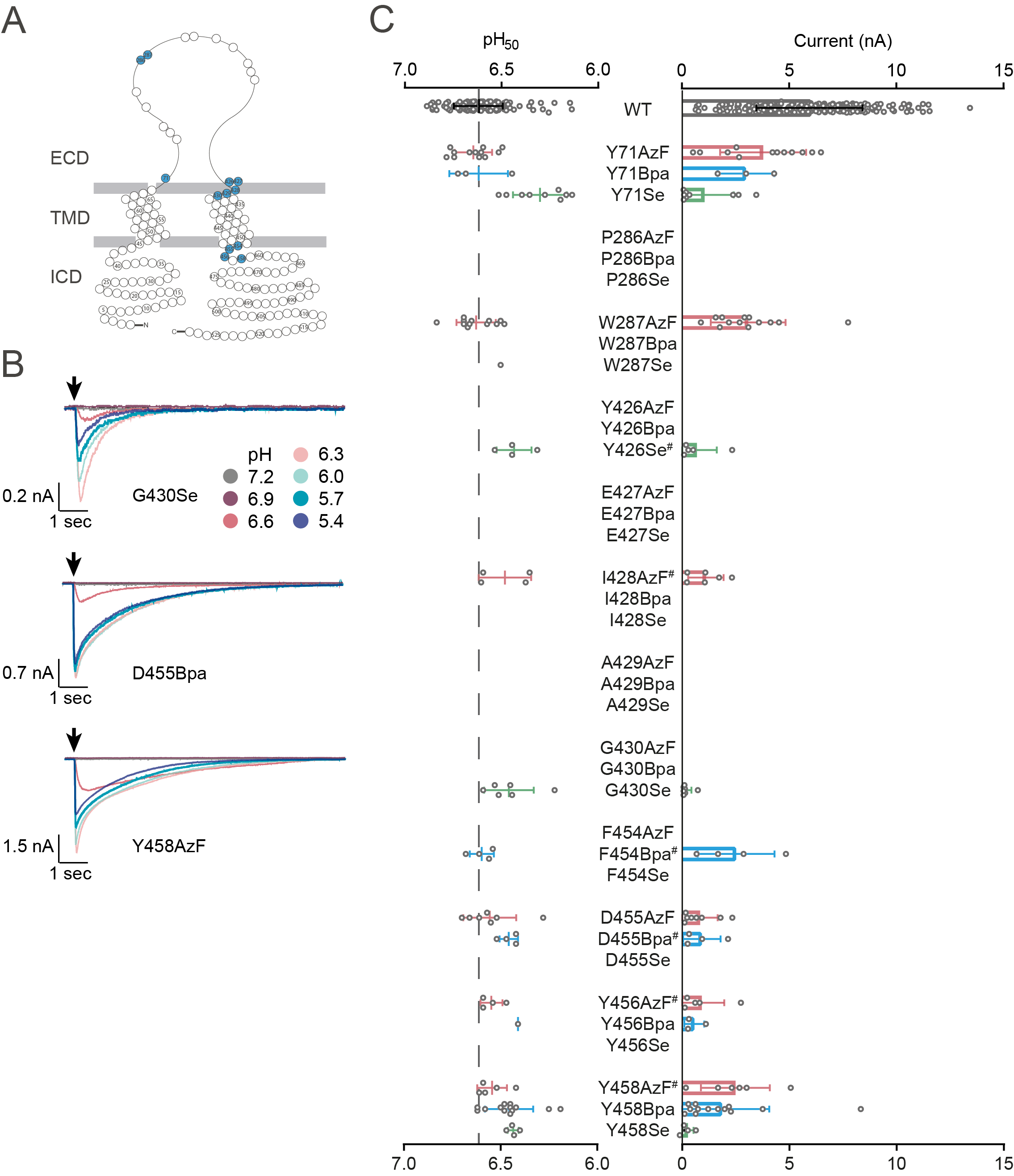
**

Supplement: S3 Fig — (A) Snake plots with tested positions marked in blue. (B) Several variants, e.g., A47AzF, Y68Bpa, G340Se, and Y458AzF, undergo tachyphylaxis after reaching the peak current. (C) Dot plots comparing pH50 (left) and peak current sizes (right); bars indicate mean ± SD, and (#) marks >20% tachyphylaxis (see also S1 Table). For variants expressed in the absence of ncAAs that yielded currents, results are marked by underlying gray bars. The underlying data have been deposited at zenodo.org (https://doi.org/10.5281/zenodo.4906985; files 19–23). hASIC1a, human acid-sensing ion channel 1a; ncAA, noncanonical amino acid; SD, standard deviation. (DOCX) [file pbio.3001321.s003.docx]

**S4 Fig.**

**
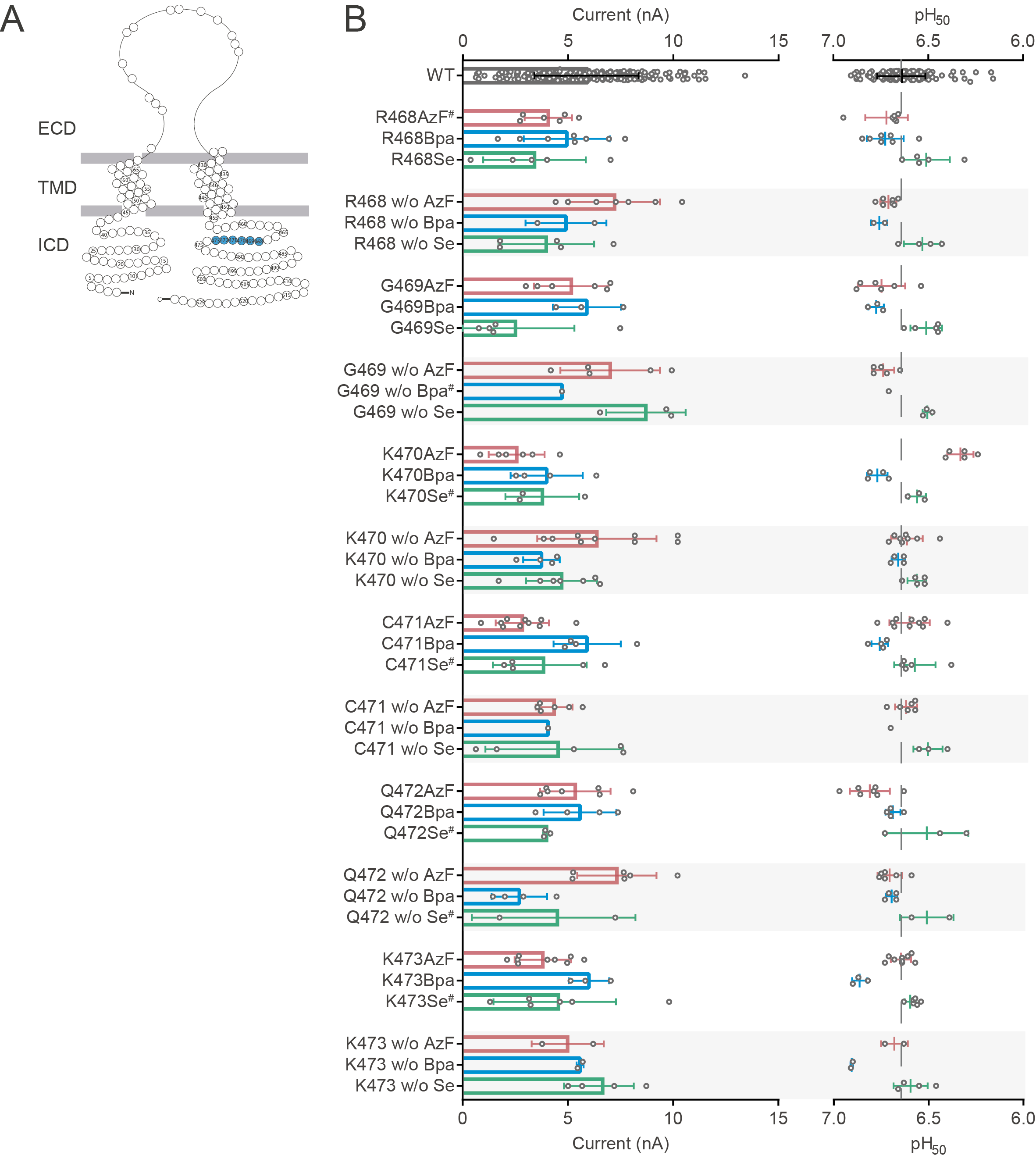
**

**
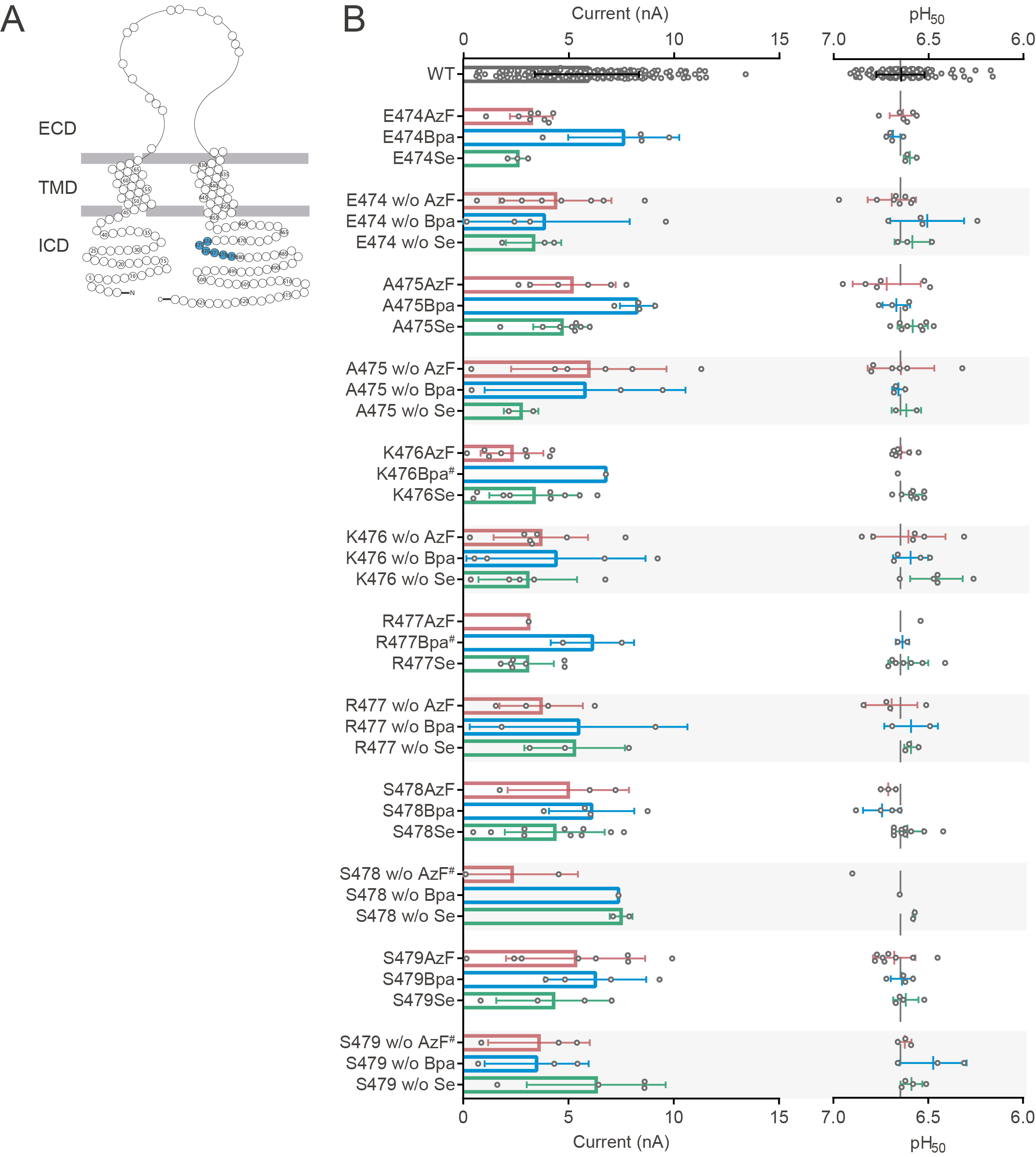
**

**
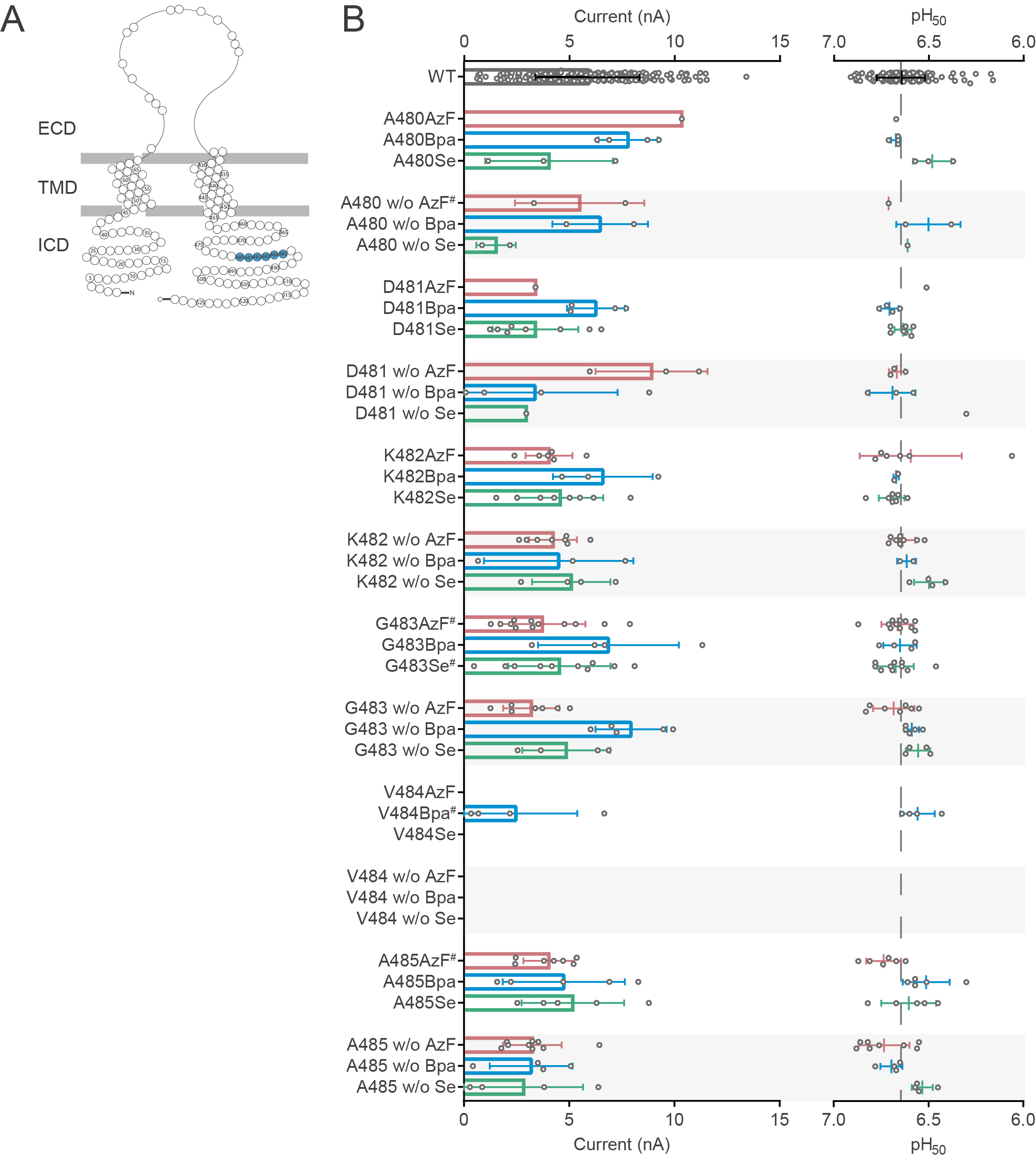
**

**
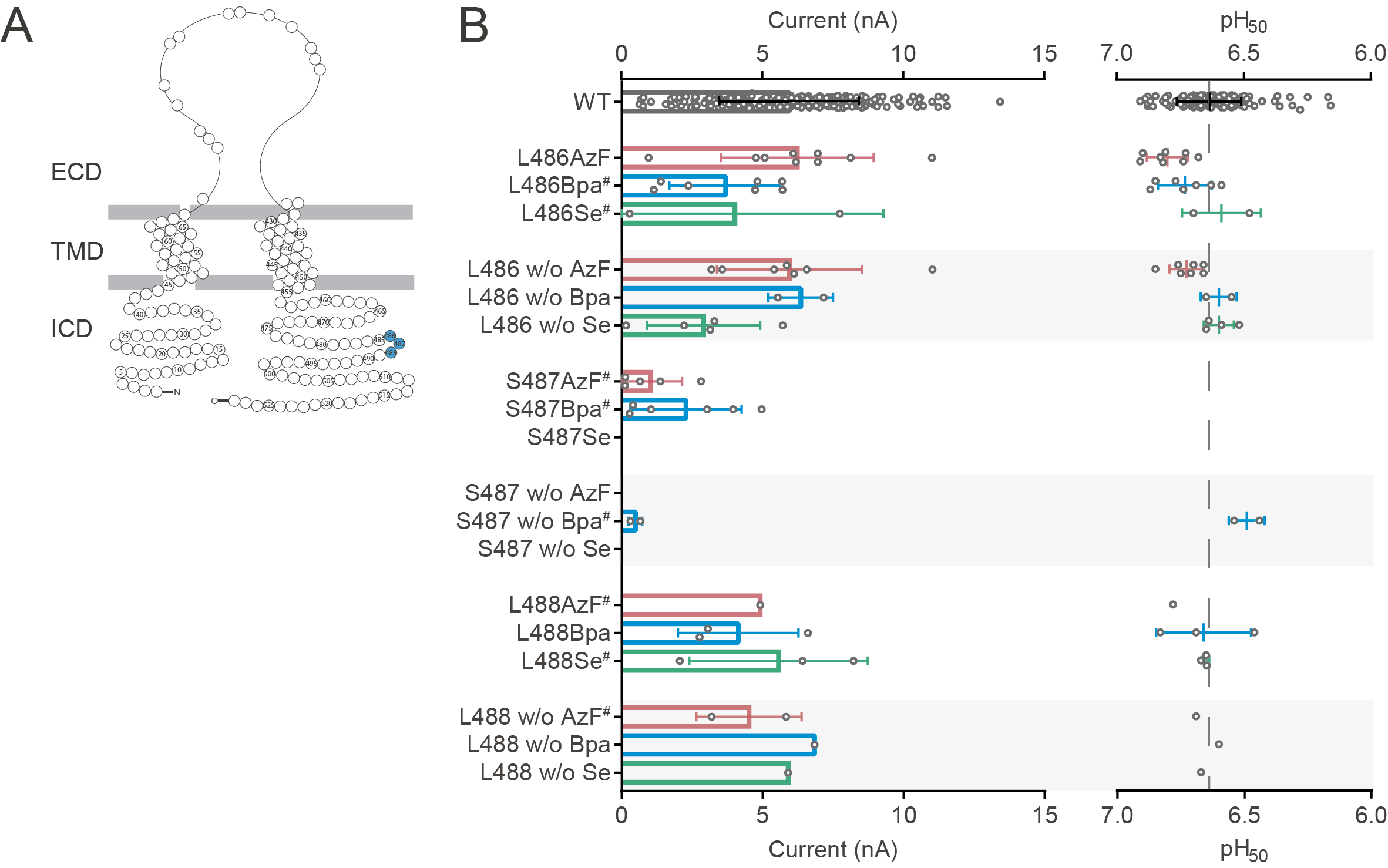
**

Supplement: S4 Fig — (A) Snake plots with tested positions marked in blue. (B) Dot plots comparing peak current sizes (left) and pH50 (right); bars indicate mean ± SD, and (#) marks >20% tachyphylaxis (see also S1 Table). For variants expressed in the absence of ncAAs that yielded currents, results are marked by underlying gray bars. The underlying data have been deposited at zenodo.org (https://doi.org/10.5281/zenodo.4906985; files 24–28). hASIC1a, human acid-sensing ion channel 1a; ncAA, noncanonical amino acid; SD, standard deviation. (DOCX) [file pbio.3001321.s004.docx]

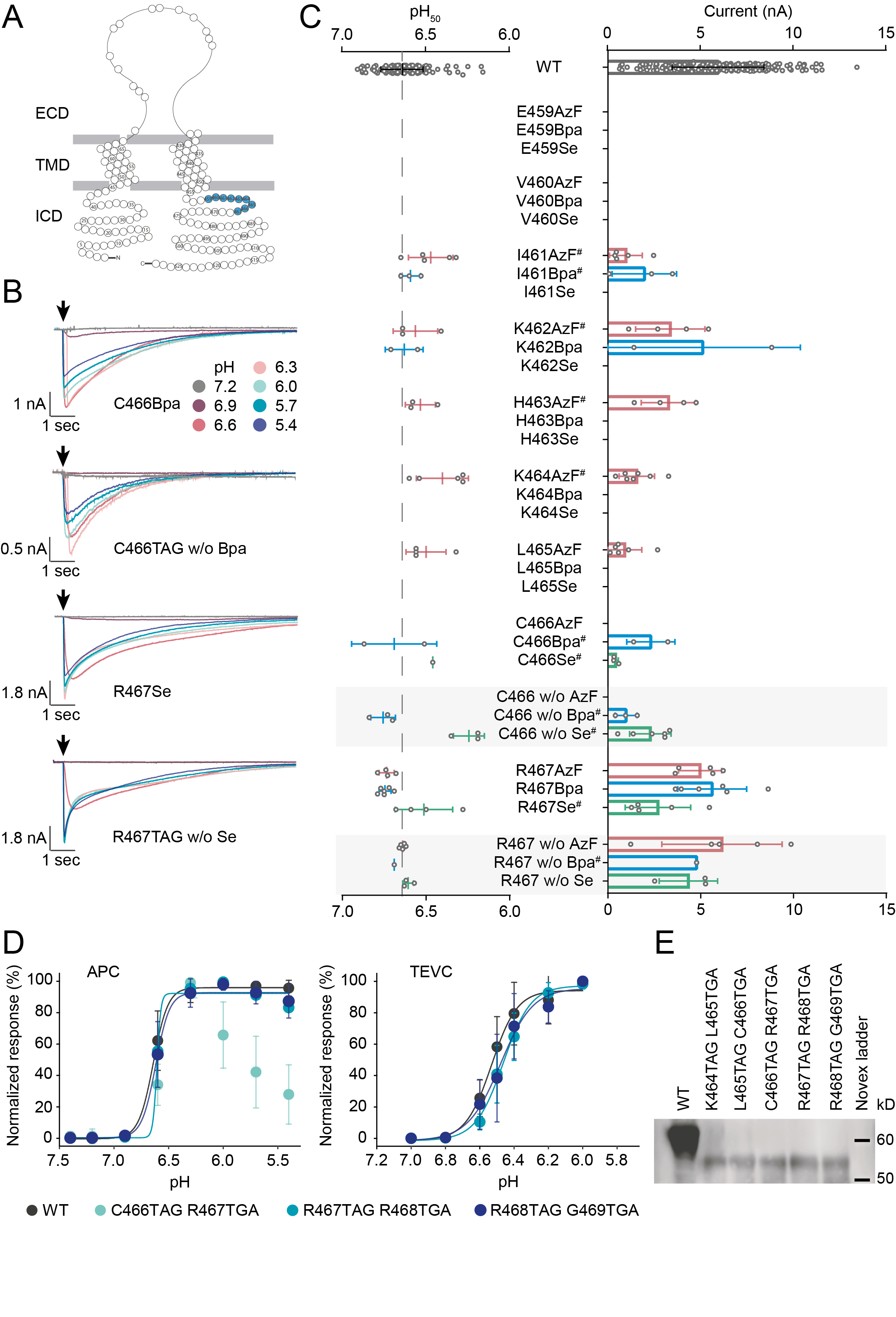

Supplement: S5 Fig — (A) Snake plot of hASIC1a highlighting carboxyl-terminal positions in blue. (B) Representative current traces of C466TAG with and without Bpa (upper panels) and R467TAG with and without Se-AbK (termed “Se,” lower panels) as recorded on the SyncroPatch 384PE. (C) Dot plots comparing pH50 (left) and peak current sizes (right); bars indicate mean ± SD and (#) marks >20% tachyphylaxis (see also S1 Table). For variants expressed in the absence of ncAAs that yielded currents, results are marked by underlying gray bars. (D) Concentration response curves of hASIC1a WT (black) and carboxyl-terminally truncated constructs recorded in HEK 293T cells (APC, left panel) and X. laevis oocytes (TEVC, right panel). (E) Western blot using an ASIC1a-antibody targeting an extracellular epitope documents truncation of the protein. The underlying data have been deposited at zenodo.org (https://doi.org/10.5281/zenodo.4906985; files 29–31). APC, automated patch clamp; Bpa, 4-Benzoyl-l-phenylalanine; hASIC1a, human acid-sensing ion channel 1a; ncAA, noncanonical amino acid; SD, standard deviation; TEVC, two-electrode voltage clamp; WT, wild type. (TIF) [file pbio.3001321.s005.tif]

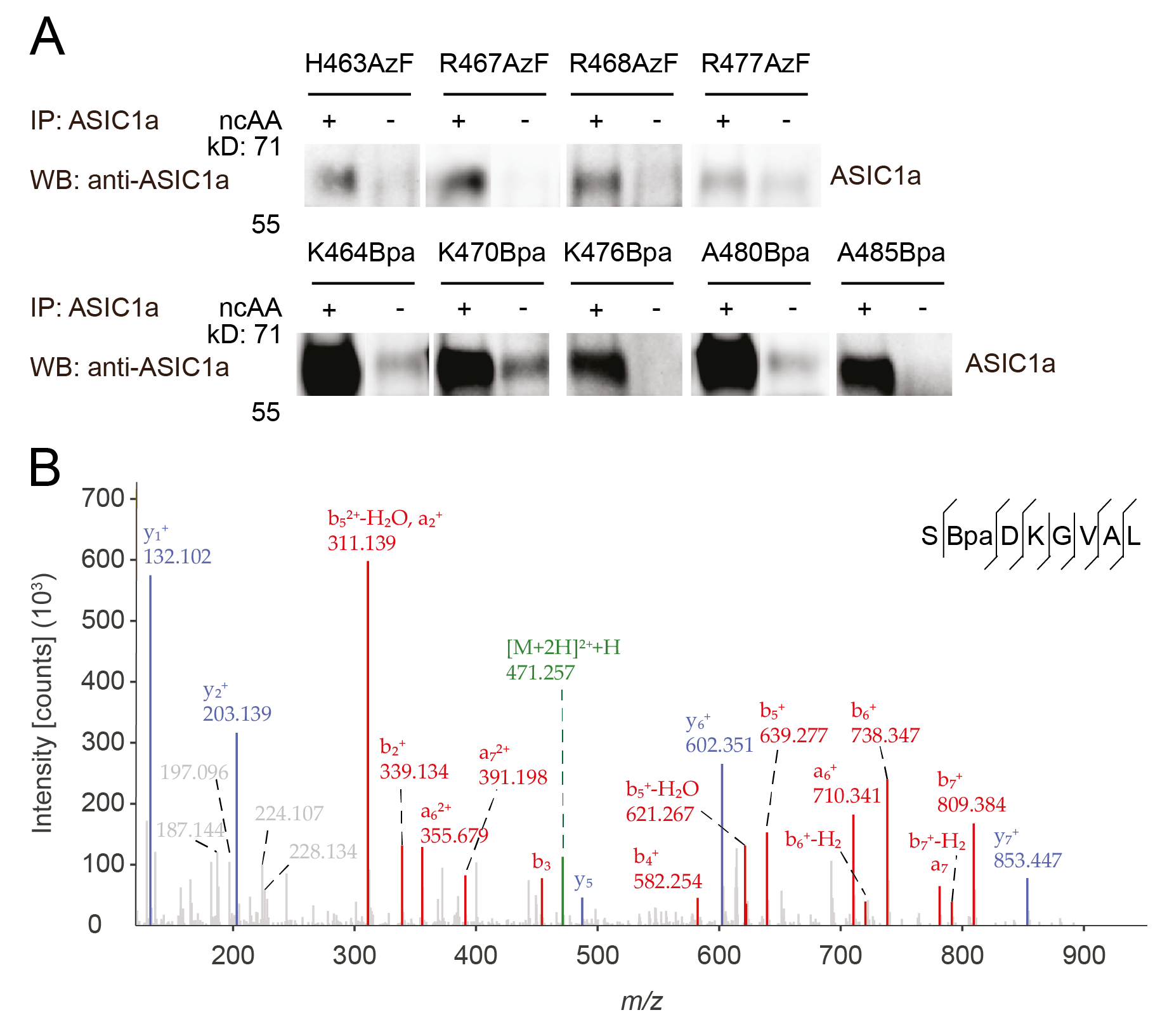

Supplement: S6 Fig — (A) Incorporation of AzF and Bpa in the carboxyl terminus is efficient with position-dependent specificity. Selected hASIC1a TAG variants were expressed in presence or absence of 10 μM AzF-ME or 1 mM Bpa in HEK 293T ASIC1a-KO cells for 48 hours; the full-length protein was purified via a carboxyl-terminal 1D4-tag and visualized by western blotting using the indicated antibody (AB). Only small amounts of full-length protein were detected in the absence of ncAA, indicating efficient incorporation. (B) Mass spectrometry confirms incorporation of Bpa at position 480. HCD fragment ion mass spectrum of the precursor peptide SBpaDKGVAL (positions 479–486, green) and the corresponding fragment ions (a- and b-ion series red, y ion series blue). Theoretical peptide mass 940.478, experimental m/z 470.743 (+0.53 ppm), charge +2. The underlying data have been deposited at zenodo.org (https://doi.org/10.5281/zenodo.4906985; file 31). AzF, 4-Azido-l-phenylalanine; Bpa, 4-Benzoyl-l-phenylalanine; hASIC1a, human acid-sensing ion channel 1a; HCD, higher-energy C-trap dissociation; ncAA, noncanonical amino acid. (TIF) [file pbio.3001321.s006.tif]

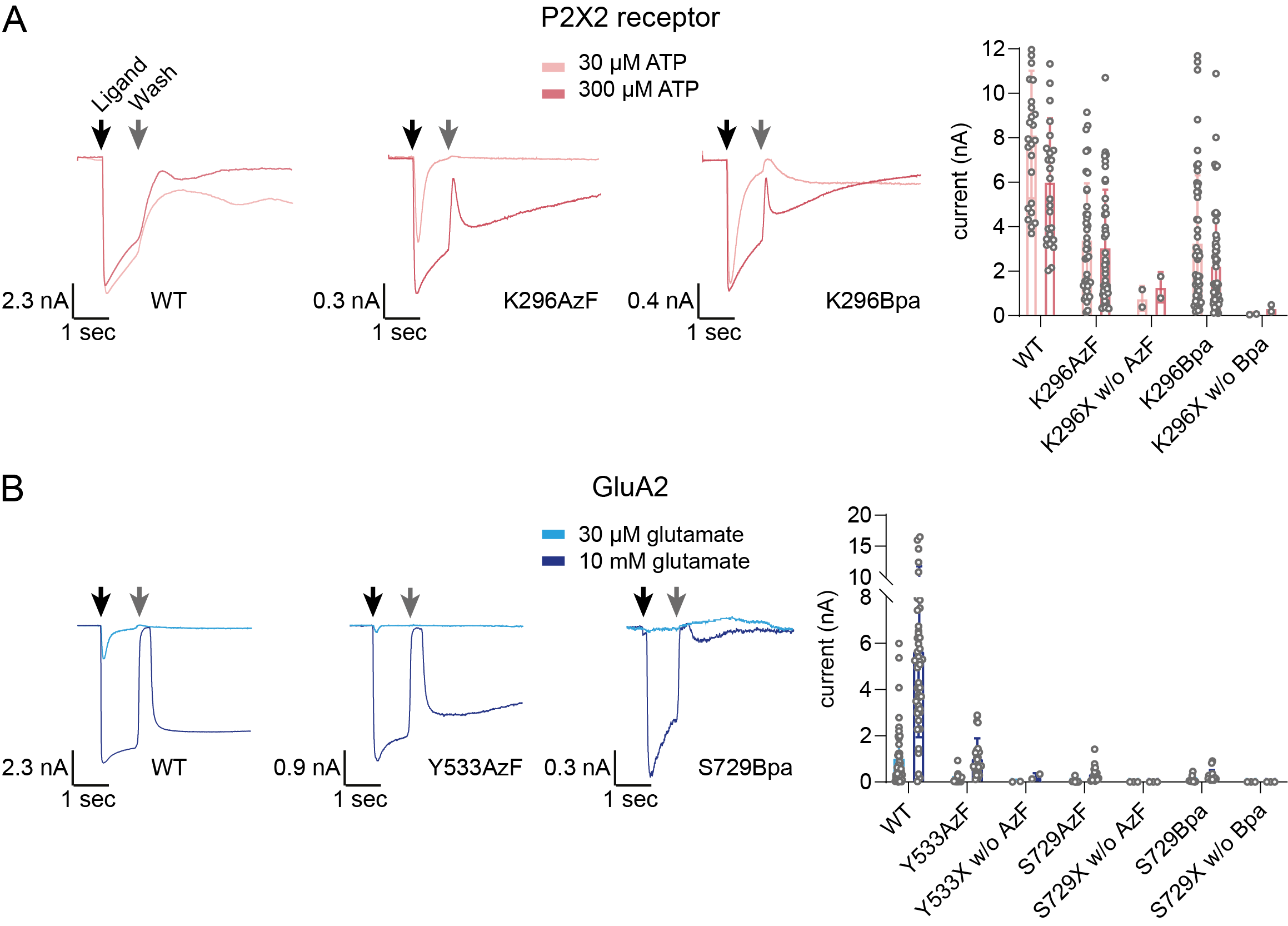

Supplement: S7 Fig — Example current traces and dot plots comparing current sizes for WT and ncAA-containing variants of the P2X2 receptor (A) and GluA2 (B) at different concentrations of ATP or glutamate, respectively. Cells expressing GluA2 were incubated with 100 μM cyclothiazide (0.8% v/v DMSO) for 1 minute before glutamate addition to reduce rapid desensitization [3]. Black arrow indicates ligand application, and gray arrow indicates addition of wash solution. As apparent from the current traces, application of the wash solution removes the ligand from the channels temporarily, but as it is still present in the well, channels can reopen, and desensitize over time. In order to enable concentration response curve measurements, all ligand has to be removed from the well. Bar graphs are mean ± SD, and values are shown in S2 Table. The underlying data have been deposited at zenodo.org (https://doi.org/10.5281/zenodo.4906985; file 32). APC, automated patch clamp; FACS, fluorescence-activated cell sorting; ncAA, noncanonical amino acid; SD, standard deviation; WT, wild type. (TIF) [file pbio.3001321.s007.tif]

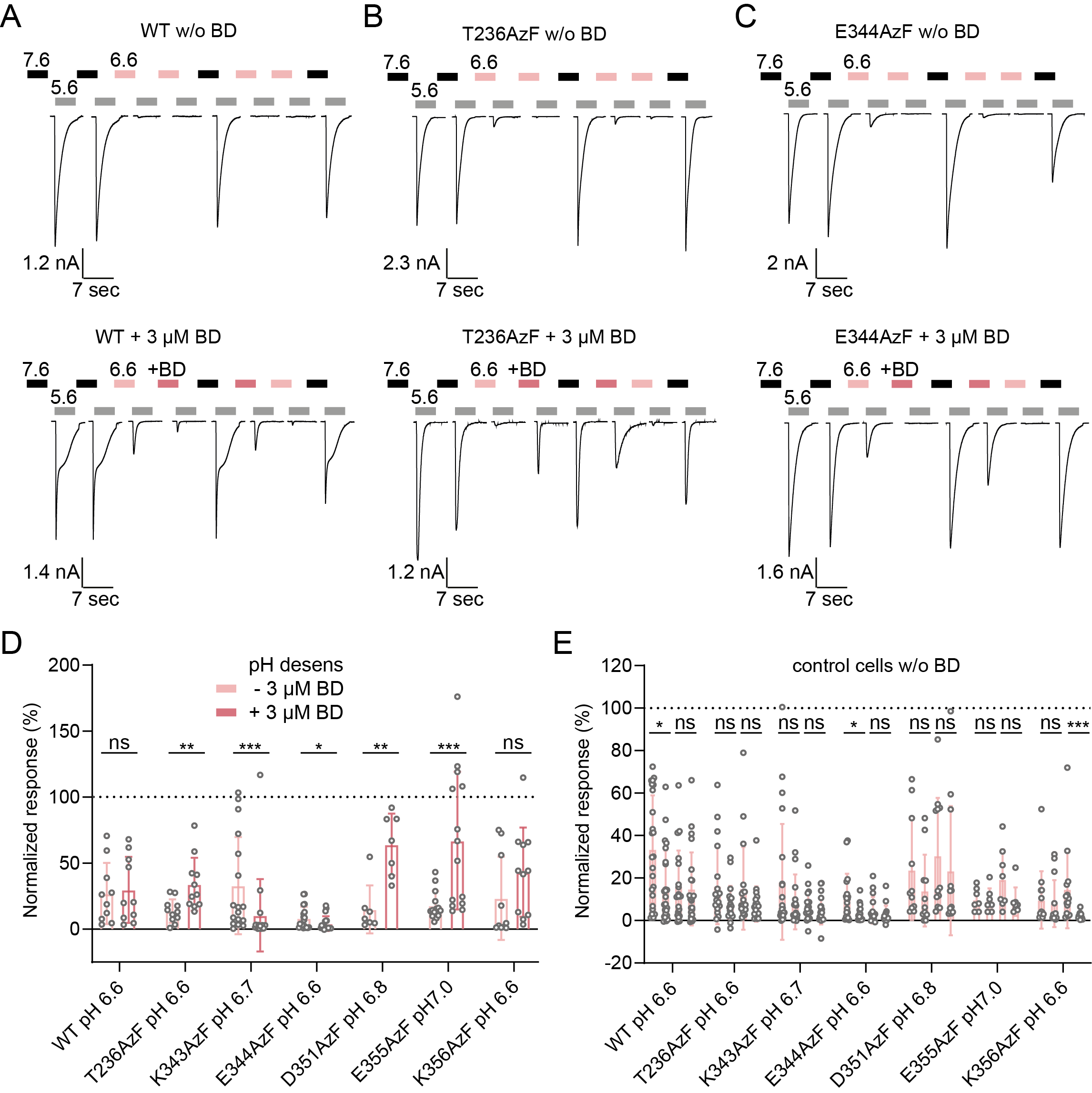

Supplement: S8 Fig — (A) Snake plot of hASIC1a highlighting assessed positions in blue. (B) Representative current traces of T239Bpa, D351AzF, and D357AzF as recorded on the SyncroPatch 384PE, with arrows indicating time of proton application. Dashed lines indicate WT current in response to pH 6.0 application. (C) Dot plots comparing pH50 (left) and peak current sizes (right); bars indicate mean ± SD, and (#) marks >20% tachyphylaxis (see also S1 Table). The underlying data have been deposited at zenodo.org (https://doi.org/10.5281/zenodo.4906985; file 33). hASIC1a, human acid-sensing ion channel 1a; PcTx1, psalmotoxin 1; SD, standard deviation; WT, wild type. (TIF) [file pbio.3001321.s008.tif]

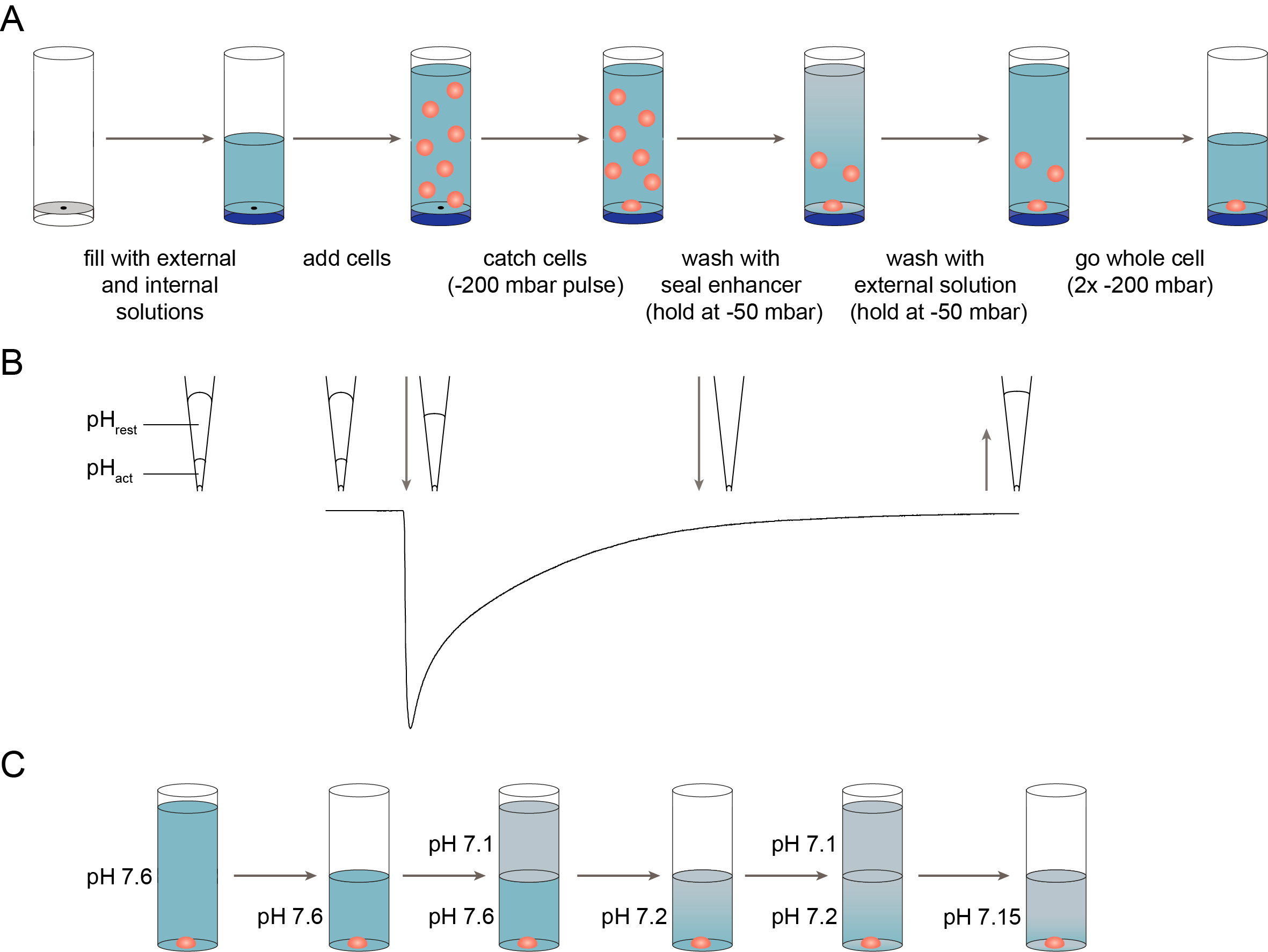

Supplement: S9 Fig — (A) After filling the wells with external and internal solution (light and dark blue), cells (orange) are added and caught on the hole by brief application of −200 mbar pressure. The cells are held in place with −50 mbar during the wash steps with seal enhancer and external solution before going into whole cell configuration via 2 pulses at −200 mbar. (B) For the stacked ligand application, pipettes are filled with 45 μl of resting pH (pHrest) followed by 5 μl solution of activating pH (pHact). Dispension of pHact leads to channel activation and desensitization in the presence of ligand, followed by dispension of pHrest with a delay of 5 seconds to wash out the ligand. Solution is slowly taken back up into the pipette at the end of each sweep, followed by a wash step with pHrest (not shown). (C) When measuring SSD curves, the open-well system of the SyncroPatch 384PE requires repeated mixing steps to approximate the target conditioning pH without disturbing the cell (orange). Moreover, 50% of the liquid (blue) are aspirated and replaced by lower pH solution (gray) twice to obtain the final conditioning pH, here pH 7.15. SSD, steady-state desensitization. (TIF) [file pbio.3001321.s009.tif]

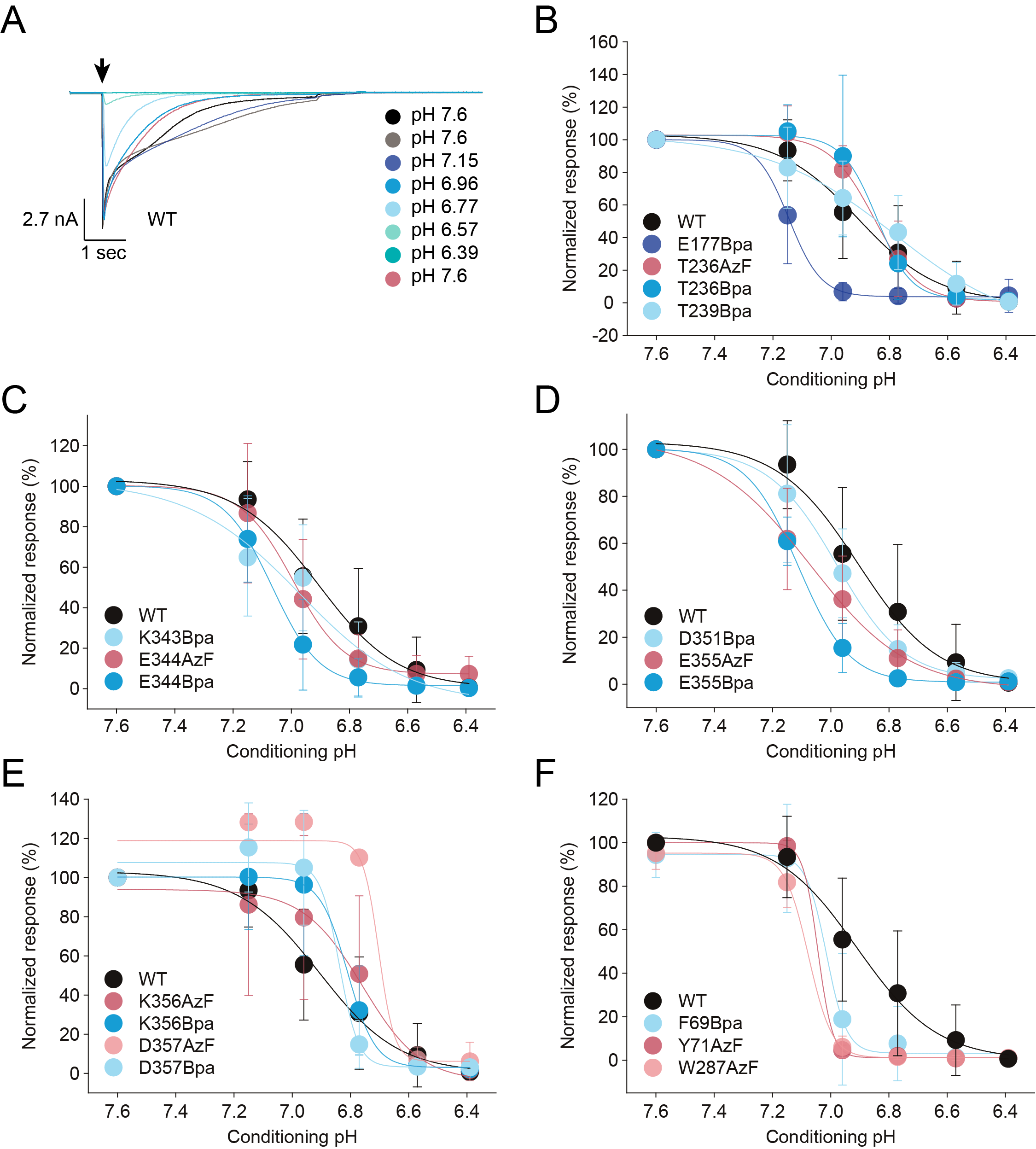

Supplement: S10 Fig — (A) Example current trace for hASIC1a WT. Currents were evoked by application of pH 5.6 after conditioning at the indicated pH values for 2 minutes. Current recovery was assessed at the end of the protocol and cells that did not regain current were excluded from the analysis. (B–F) SSD curves of WT, 14 variants carrying AzF or Bpa in the acidic pocket, and 3 control positions in the interface region. Currents were normalized to the mean of the first 2 applications. See S4 Table for pH50 SSD values, nH and n. The underlying data have been deposited at zenodo.org (https://doi.org/10.5281/zenodo.4906985; files 34 and 35). APC, automated patch clamp; AzF, 4-Azido-l-phenylalanine; Bpa, 4-Benzoyl-l-phenylalanine; hASIC1a, human acid-sensing ion channel 1a; SSD, steady-state desensitization; WT, wild type. (TIF) [file pbio.3001321.s010.tif]

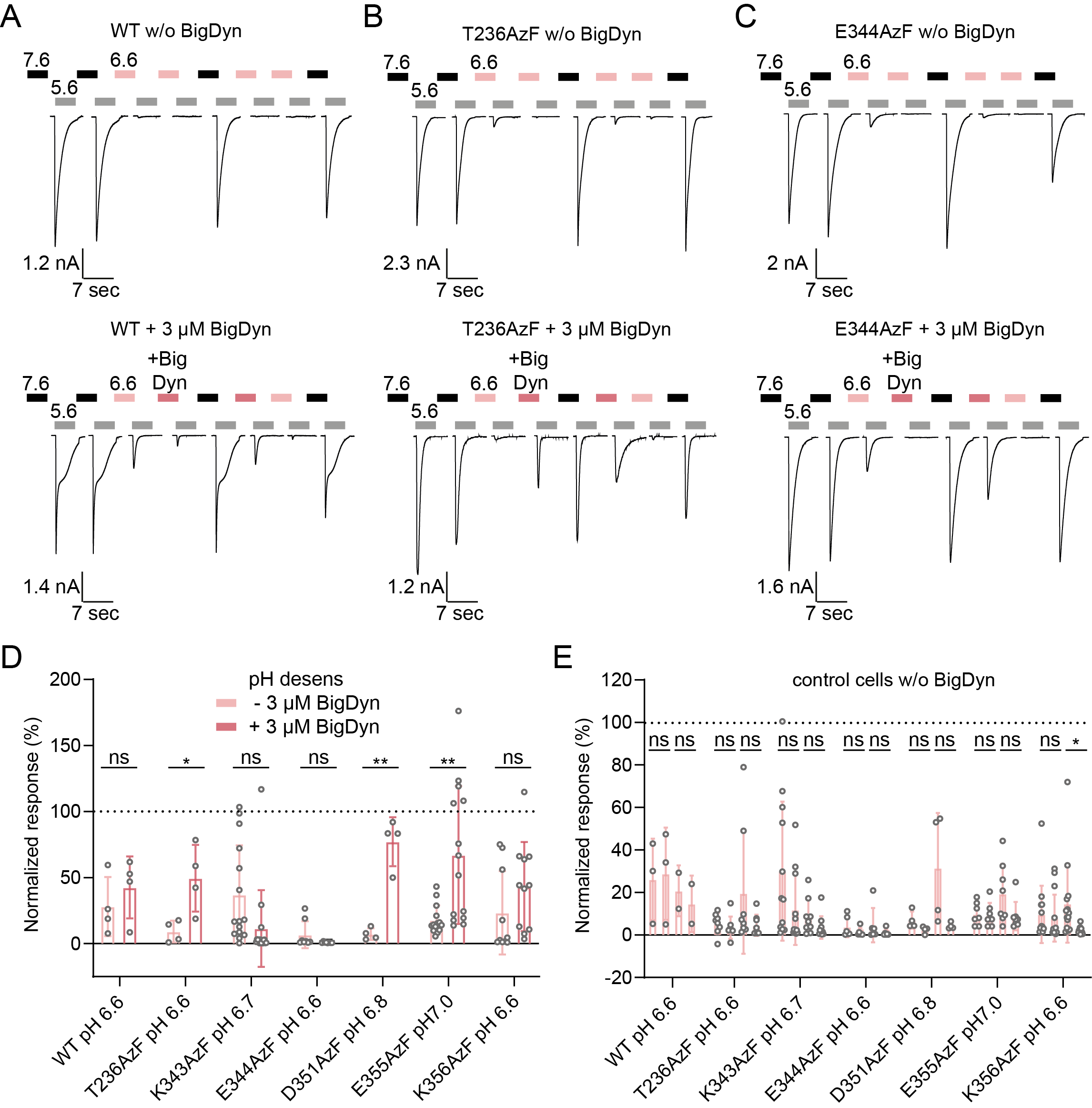

Supplement: S11 Fig — (A–C) Characteristic current traces of the full APC protocols for WT (A), T236AzF (B), and E344AzF (C) with and without 3 μM BigDyn (lower versus upper panel). Cells were first exposed to 2 activation pulses with pH 5.6 (gray bars, 5 seconds) after conditioning at pH 7.6 (black bars) to determine the control current, followed by 2 rounds of activation after 2 minutes conditioning with a pH that induces SSD (light pink bars) and a control pulse to evaluate current recovery. For half of the cell population, 3 μM BigDyn (dark pink bars) were co-applied during the second conditioning period to measure rescue from SSD. This assessment of SSD and recovery was repeated with peptide co-application during the first SSD conditioning to also evaluate peptide wash out. Currents were normalized to the average of the first 2 control pulses to compare modulation at different conditions (values in S5 Table, pink and black bars not to scale). (D) Bar graph comparing current after SSD in absence and presence of 3 μM BigDyn (traces 3+4 in A–C, lower panel). (E) Bar graph comparing current after SSD for control cells not exposed to BigDyn (traces 3+4 and 6+7 in A–C, upper panel). Bar graphs show mean ± SD, dashed line indicates 100%, and values are shown in S5 Table. (*) denotes significant difference between groups, p < 0.05; (**): p < 0.01; (***): p < 0.001; ns: not significant; Mann–Whitney test. The underlying data have been deposited at zenodo.org (https://doi.org/10.5281/zenodo.4906985; file 11). APC, automated patch clamp; AzF, 4-Azido-l-phenylalanine; hASIC1a, human acid-sensing ion channel 1a; SSD, steady-state desensitization; WT, wild type. (TIF) [file pbio.3001321.s011.tif]

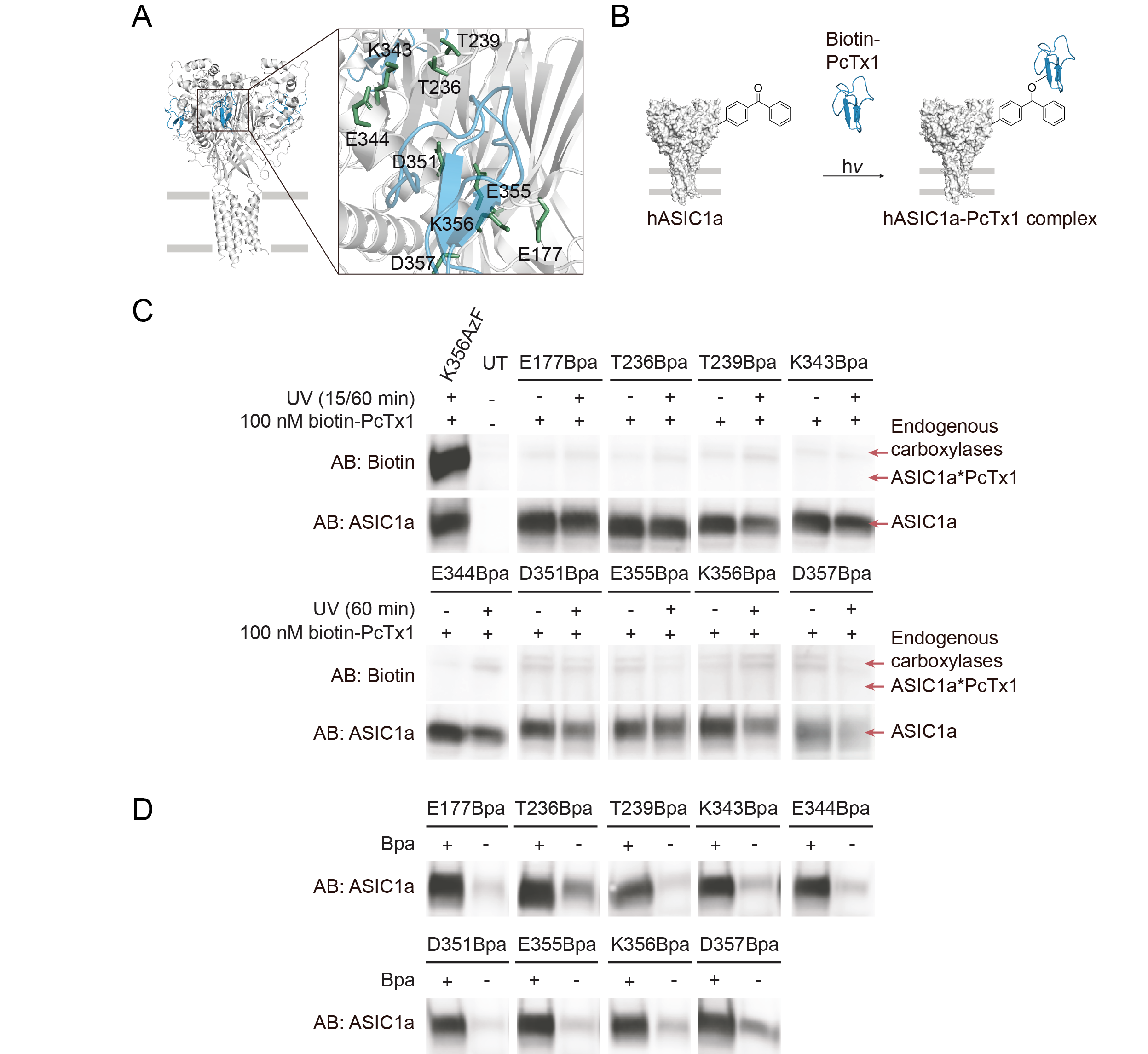

Supplement: S12 Fig — (A) Structure of cASIC1 (white) in complex with PcTx1 (blue, PDB: 4FZ0), inset shows individual side chains replaced by Bpa in the acidic pocket (green), none of which crosslinked to biotin-PcTx1. (B) Schematic workflow for Bpa crosslinking to biotin-PcTx1 (see also Fig 5). (C) Western blot of purified hASIC1a K356AzF, UT cells, and variants carrying Bpa in the ECD detected using the specified antibodies (AB). Biotin-PcTx1 is only detected in the control sample containing AzF at position 356 (15 minutes UV exposure), but not in any of the 9 positions containing Bpa (60 minutes UV exposure, positions colored green in A). The detected double band by the anti-biotin AB originates from endogenous biotin-dependent carboxylases [4, 5]. (D) Control experiments demonstrating efficient Bpa incorporation at all positions tested for crosslinking in C. Stop-codon containing hASIC1a mutants were grown in the presence or absence of 1 mM Bpa in HEK 293T cells for 48 hours, after which the resulting full-length protein was purified via a carboxyl-terminal 1D4-tag and visualized by western blotting using the indicated antibody (AB). With the exception of positions 236 and 357, only small amounts of full-length protein were detected in absence of Bpa (compared to those obtained in its presence), demonstrating efficient incorporation. The underlying data have been deposited at zenodo.org (https://doi.org/10.5281/zenodo.4906985; file 13). AzF, 4-Azido-l-phenylalanine; Bpa, 4-Benzoyl-l-phenylalanine; cASIC1, chicken acid-sensing ion channel 1; ECD, extracellular domain; hASIC1a, human acid-sensing ion channel 1a; PcTx1, psalmotoxin 1; PDB, Protein Data Bank; UT, untransfected. (TIF) [file pbio.3001321.s012.tif]

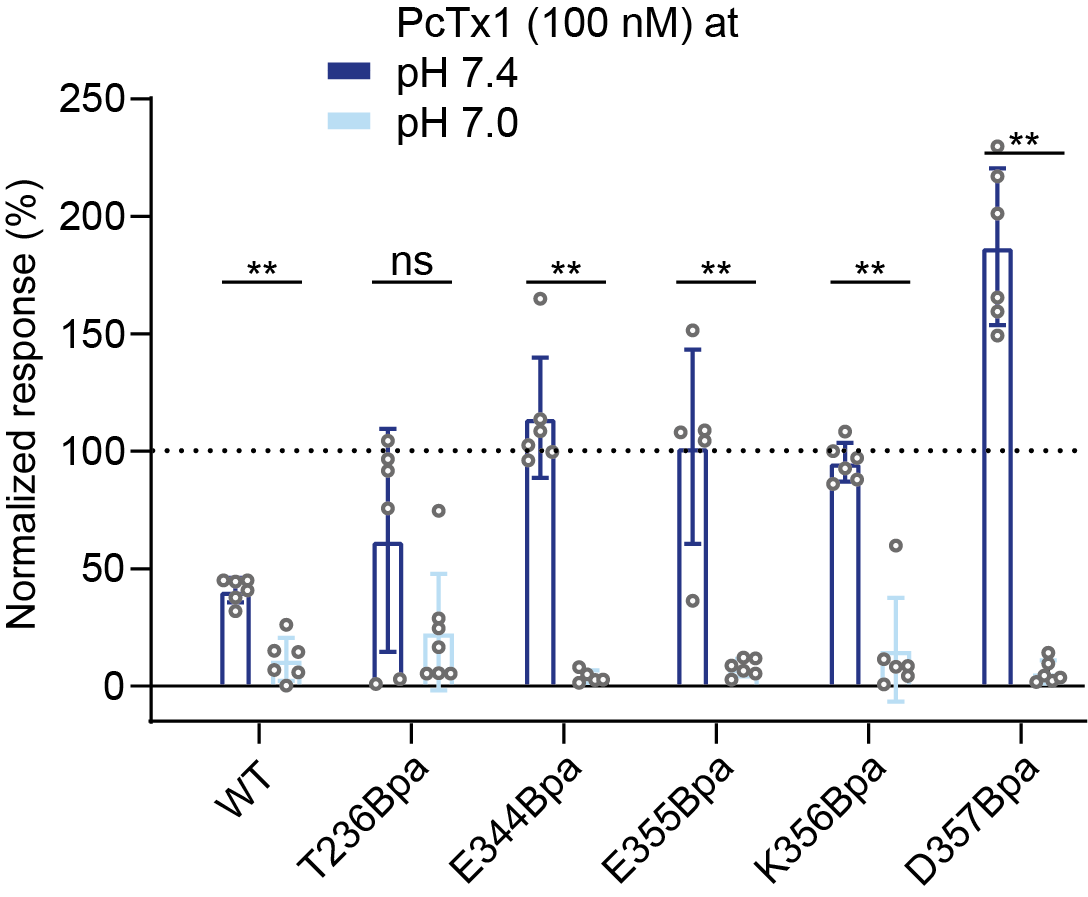

Supplement: S13 Fig — Cells were incubated with 100 nM PcTx1 at varying conditioning pH for 2 minutes before activation at pH 5.6, and the current was normalized to the average of the 4 preceding and following control currents after conditioning at pH 7.4. Bar graph shows mean ± SD, dashed line indicates 100%, and values are shown in S6 Table. (**) denotes significant difference between groups, p < 0.01; ns: not significant; Mann–Whitney test. The underlying data have been deposited at zenodo.org (https://doi.org/10.5281/zenodo.4906985; file 12). Bpa, 4-Benzoyl-l-phenylalanine; hASIC1a, human acid-sensing ion channel 1a; PcTx1, psalmotoxin 1; SD, standard deviation; WT, wild type. (TIF) [file pbio.3001321.s013.tif]

**S14 Fig.**

**
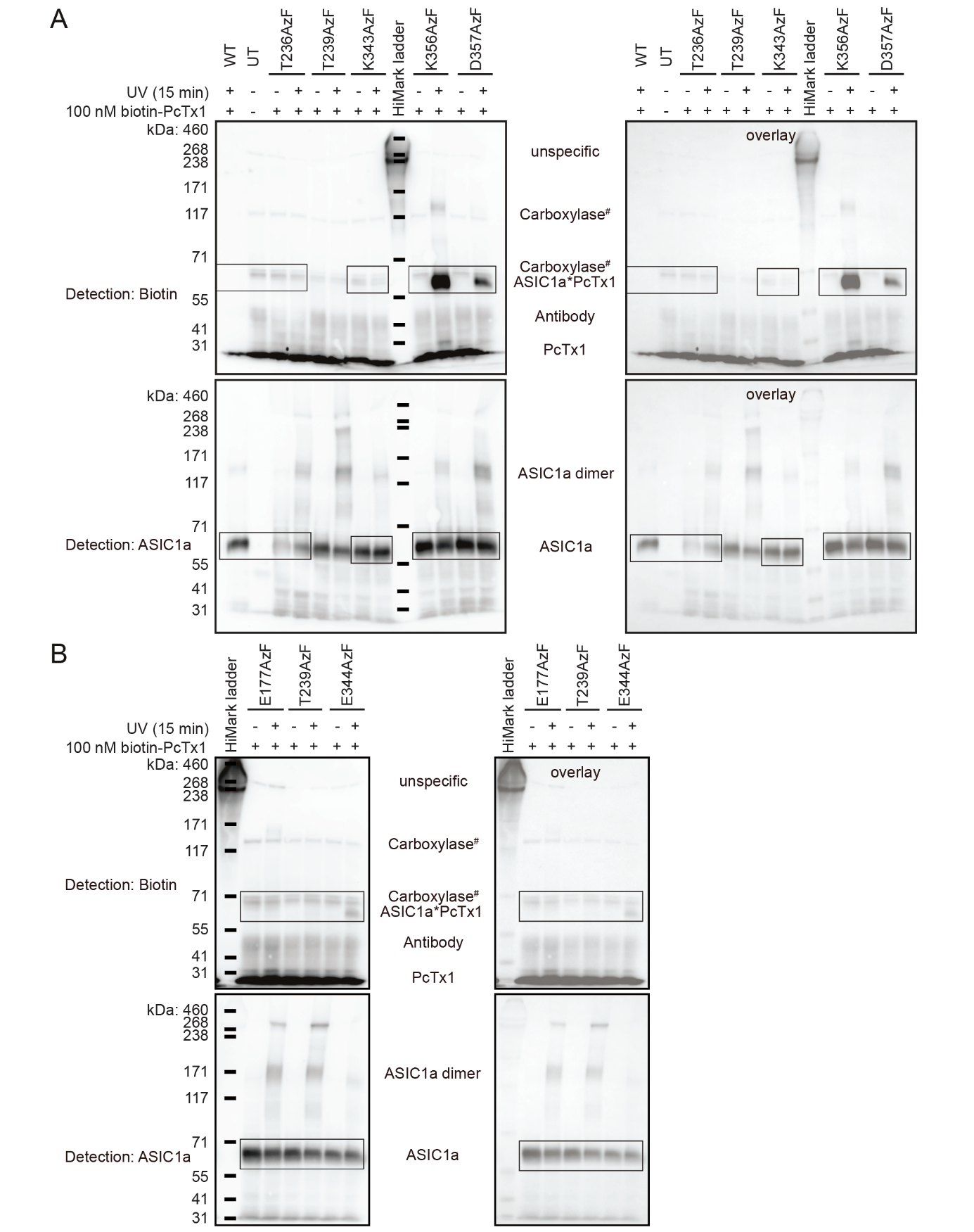
**

**
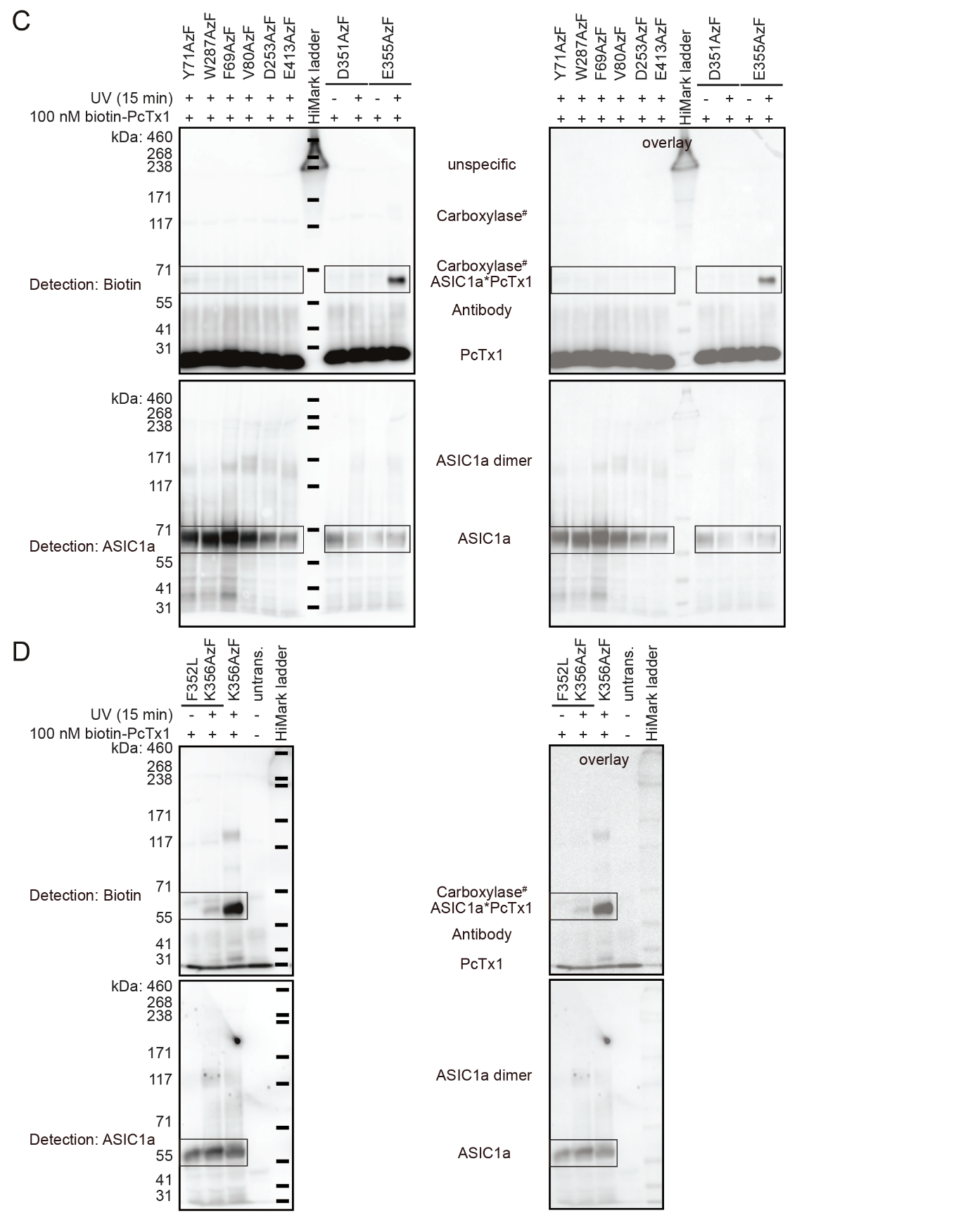
**

Supplement: S14 Fig — Black bars indicate protein ladders for clarity (left panel), and original markers (coomassie) are overlayed with the blot (chemiluminescence, right panel). Areas cropped for Fig 5 are marked with boxes. (A) Western blot for positions 236, 239, 343, 356, and 357. (B) Western blot for positions 177, 239, and 344. (C) Western blot for positions 71, 287, 69, 80, 253, 413, 351, and 355. (D) Western blot for the F352L K356AzF double mutant. Data are representative of 2–3 individual experiments. Control experiments demonstrating efficient AzF incorporation for all above positions are published in [6]. The data have also been deposited at zenodo.org (https://doi.org/10.5281/zenodo.4906985; file 13). AzF, 4-Azido-l-phenylalanine. (DOCX) [file pbio.3001321.s014.docx]

**S15 Fig.**

**
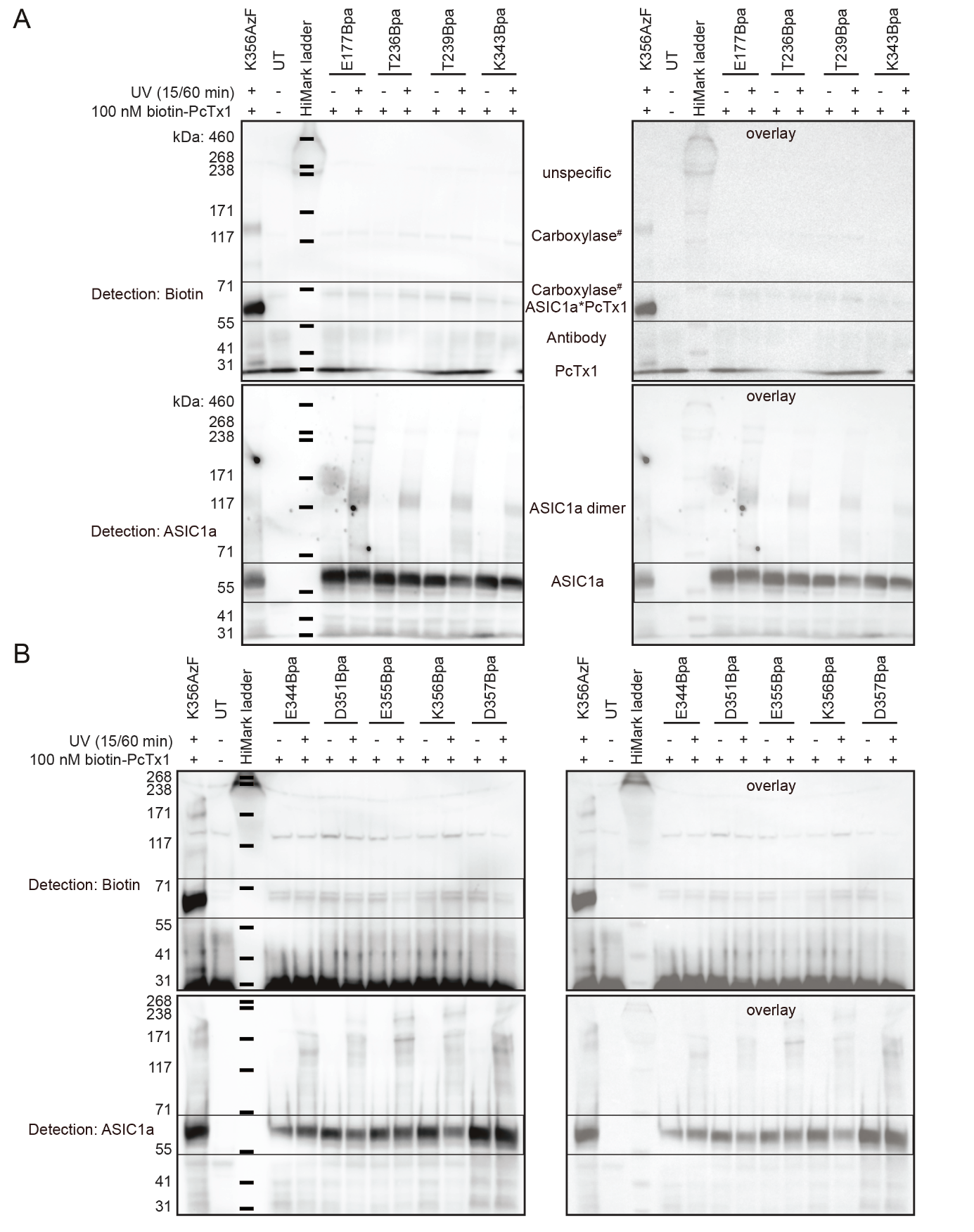
**

**
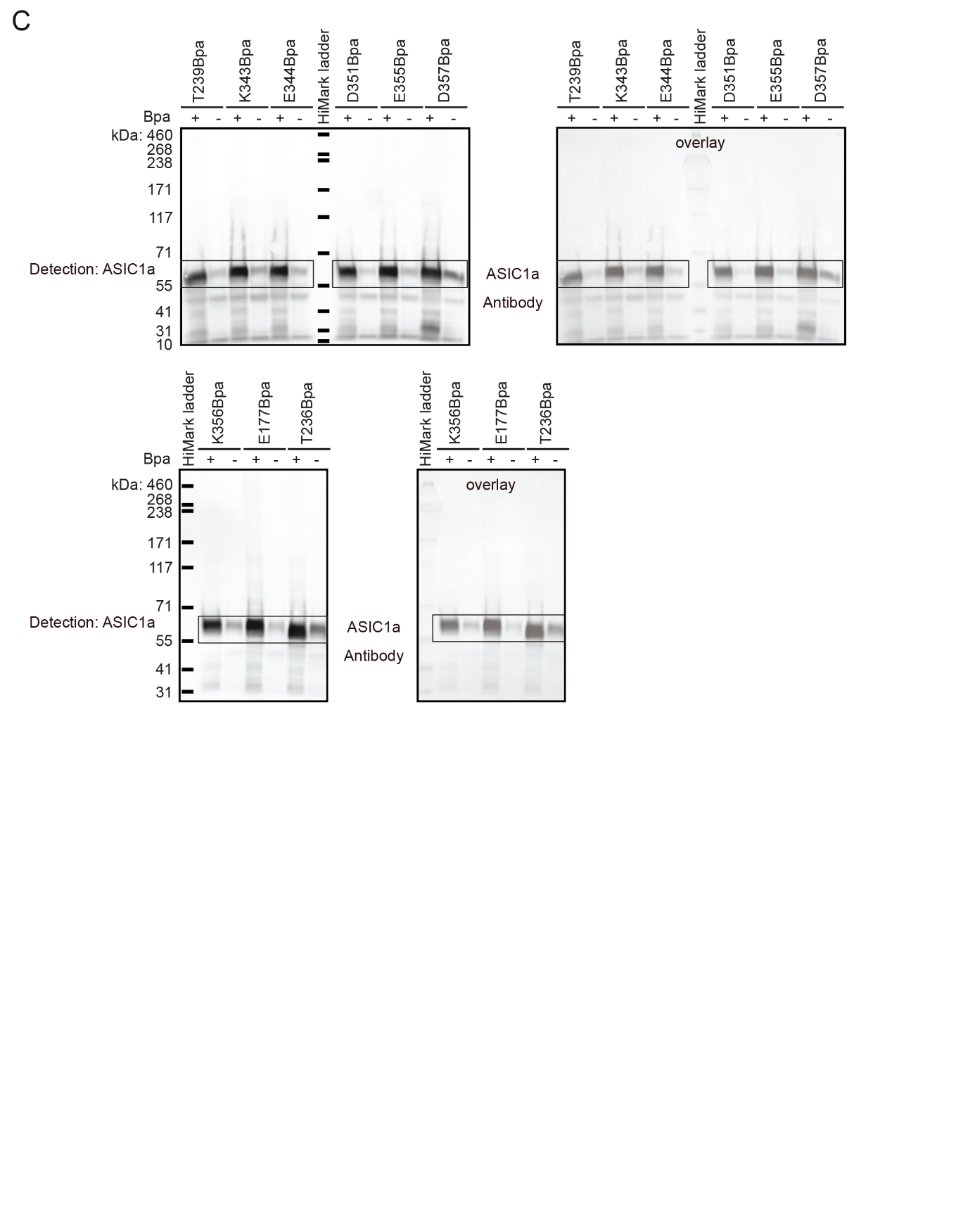
**

Supplement: S15 Fig — Black bars indicate protein ladders for clarity (left panel), and original markers (coomassie) are overlayed with the blot (chemiluminescence, right panel). Areas cropped for S12 Fig are marked with boxes. (A) Western blot for positions 177, 236, 239, and 343, including K356AzF as a positive control. (B) Western blot for positions 344, 351, 355, 356, and 357, including K356AzF as a positive control. (C) Western blot demonstrating efficient Bpa incorporation at positions 239, 343, 344, 351, 355, and 357 (upper panel) and at positions 177, 236, and 356 (lower panel). Data are representative of 2–3 individual experiments. The data have also been deposited at zenodo.org (https://doi.org/10.5281/zenodo.4906985; file 13). Bpa, 4-Benzoyl-l-phenylalanine. (DOCX) [file pbio.3001321.s015.docx]
