# Supplementary material for: High-throughput characterization of photocrosslinker-bearing ion channel variants to map residues critical for function and pharmacology
Source: PLoS Biol. 2021 Sep 7;19(9):e3001321. doi: 10.1371/journal.pbio.3001321 (PMC8448361; doi:10.1371/journal.pbio.3001321)
Supplement: S1 Table — Values for pH50, nH and Imax are shown as mean ± SD for n ≥ 3 and as averages for n = 1–2. (#) indicates pronounced tachyphylaxis (final sweep <80% of normalized peak current). Average TE is shown in (%) as measured via GFP fluorescence on the FACS instrument. The underlying data have been deposited at zenodo.org (https://doi.org/10.5281/zenodo.4906985; files 02–06, 09, 15–28, and 33). FACS, fluorescence-activated cell sorting; hASIC1a, human acid-sensing ion channel 1a; SD, standard deviation; TE, transfection efficiency. (DOCX) [file pbio.3001321.s016.docx]

| Position replaced by TAG | ncAA | mean pH_50_  ± S.D. (n) | mean n_H_  ± S.D. (n) | mean Imax (nA) ± S.D. (n) | average  TE (%) |
| --- | --- | --- | --- | --- | --- |
| E6 | AzF^#^ | 6.69 ± 0.07 (8) | 8.66 ± 2.76 (8) | 3.82 ± 1.65 (10) | 12.2 |
|  | AzF neg. | n.a. | n.a. | 0 (0) | n.a. |
|  | Bpa | 6.55 (2) | 8.76 (2) | 2.87 (2) | 6.8 |
|  | Bpa neg. | 6.44 (2) | 8.42 (2) | 1.14 (2) | n.a. |
|  | Se-AbK^#^ | 6.57 ± 0.09 (6) | 7.66 ± 3.31 (3) | 1.8 ± 2.2 (8) | 5.8 |
|  | Se-AbK neg. | n.a. | n.a. | 0 (0) | n.a. |
| E7 | AzF^#^ | 6.58 ± 0.08 (7) | 7.78 ± 6.04 (6) | 2.47 ± 1.56 (9) | 25.2 |
|  | AzF neg. | n.a. | n.a. | 1.01 (1) | n.a. |
|  | Bpa^#^ | 6.54 ± 0.07 (6) | 6.8 ± 3.45 (6) | 1.12 ± 1.1 (7) | 12.7 |
|  | Bpa neg. ^#^ | 6.45 (1) | 7.81 (1) | 0.12 (2) | n.a. |
|  | Se-AbK^#^ | 6.6 ± 0.04 (5) | 7.54 (2) | 2.41 ± 2.58 (5) | 5 |
|  | Se-AbK neg. | 6.59 (2) | 32.34 (2) | 0.99 (2) | n.a. |
| E8 | AzF | 6.51 ± 0.08 (4) | 6.29 ± 0.98 (3) | 4.7 ± 4.53 (4) | 13.5 |
|  | AzF neg. | n.a. | n.a. | 0 (0) | n.a. |
|  | Bpa | 6.44 ± 0.07 (10) | 5.61 ± 1.4 (10) | 1.86 ± 1.12 (10) | 10.5 |
|  | Bpa neg. | 6.44 (1) | 6.13 (1) | 0.23 (1) | n.a. |
|  | Se-AbK^#^ | 6.55 ± 0.06 (4) | 9.3 ± 2.55 (4) | 2.17 ± 2.22 (4) | 3.9 |
|  | Se-AbK neg. | n.a. | n.a. | 0 (0) | n.a. |
| E9 | AzF | 6.56 ± 0.06 (10) | 6.66 ± 4.37 (8) | 2.33 ± 1.37 (10) | 13.3 |
|  | AzF neg. | n.a. | n.a. | 0 (0) | n.a. |
|  | Bpa^#^ | 6.59 ± 0.04 (10) | 7.80 ± 1.78 (7) | 1.99 ± 1.96 (11) | 13.8 |
|  | Bpa neg. | 6.43 ± 0.16 (3) | 10.8 ± 3.09 (3) | 1.57 ± 1.13 (3) | n.a. |
|  | Se-AbK^#^ | 6.60 ± 0.04 (7) | 7.32 ± 2.06 (4) | 0.42 ± 0.23 (8) | 6 |
|  | Se-AbK neg.^#^ | 6.55 ± 0.06 (3) | 7.25 (2) | 0.85 ± 0.4 (3) | n.a. |
| V10 | AzF^#^ | 6.54 ± 0.07 (12) | 6.66 ± 2.98 (7) | 1.56 ± 1.12 (13) | 6.1 |
|  | AzF neg. | n.a. | n.a. | 0 (0) | n.a. |
|  | Bpa | n.a. | n.a. | 0 (0) | 20.7 |
|  | Bpa neg. | n.a. | n.a. | 0 (0) | n.a. |
|  | Se-AbK^#^ | 6.58 ± 0.01 (3) | 9.94 (1) | 0.91 ± 0.89 (4) | 7.7 |
|  | Se-AbK neg. | n.a. | n.a. | 0 (0) | n.a. |
| G11 | AzF^#^ | 6.51 ± 0.06 (6) | 8.0 ± 2.56 (5) | 0.72 ± 0.22 (7) | 6.9 |
|  | AzF neg. | n.a. | n.a. | 0 (0) | n.a. |
|  | Bpa | 6.44 ± 0.12 (9) | 6.21 ± 1.96 (9) | 2.35 ± 1.04 (10) | 18.3 |
|  | Bpa neg. | n.a. | n.a. | 0 (0) | n.a. |
|  | Se-AbK^#^ | 6.6 (1) | 18.1 (1) | 0.28 (2) | 4.1 |
|  | Se-AbK neg. | n.a. | n.a. | 0 (0) | n.a. |
| G12^#^ | AzF^#^ | 6.54 ± 0.06 (13) | 7.61 ± 2.53 (13) | 2.04 ± 1.68 (14) | 14.6 |
|  | AzF neg. | n.a. | n.a. | 0 (0) | n.a. |
|  | Bpa^#^ | 6.57 ± 0.07 (4) | 8.53 ± 5.01 (3) | 1.45 ± 1.56 (6) | 15.1 |
|  | Bpa neg. | n.a. | n.a. | 0 (0) | n.a. |
|  | Se-AbK^#^ | 6.54 ± 0.08 (5) | 3.93 (2) | 0.47 ± 0.35 (6) | 6.7 |
|  | Se-AbK neg. | n.a. | n.a. | 0 (0) | n.a. |

| Position replaced by TAG | ncAA | mean pH_50_  ± S.D. (n) | mean n_H_  ± S.D. (n) | mean Imax (nA) ± S.D. (n) | average  TE (%) |
| --- | --- | --- | --- | --- | --- |
| V13 | AzF | 6.60 ± 0.07 (8) | 7.28 ± 1.77 (6) | 3.66 ± 2.31 (8) | 6.9 |
|  | AzF neg. | n.a. | n.a. | 0 (0) | n.a. |
|  | Bpa | 6.61 ± 0.08 (10) | 5.28 ± 1.44 (10) | 4.92 ± 2.16 (10) | 12.1 |
|  | Bpa neg. | n.a. | n.a. | 0.1 (1) | n.a. |
|  | Se-AbK^#^ | 6.58 ± 0.01 (7) | 8.3 ± 1.02 (4) | 1.02 ± 1.25 (8) | 5 |
|  | Se-AbK neg. | n.a. | n.a. | 0 (0) | n.a. |
| Q14 | AzF | 6.61 ± 0.05 (13) | 7.23 ± 1.66 (13) | 3.75 ± 1.48 (15) | 8.4 |
|  | AzF neg. | n.a. | n.a. | 0 (0) | n.a. |
|  | Bpa | 6.67 ± 0.2 (3) | 4.49 (2) | 4.05 ± 0.96 (3) | 6.6 |
|  | Bpa neg. | n.a. | n.a. | 0 (0) | n.a. |
|  | Se-AbK^#^ | 6.63 ± 0.04 (8) | 6.85 ± 1.38 (6) | 1.47 ± 1.09 (10) | 8.4 |
|  | Se-AbK neg. | n.a. | n.a. | 0 (0) | n.a. |
| P15 | AzF^#^ | 6.59 ± 0.06 (11) | 6.27 ± 2.16 (11) | 3.52 ± 1.47 (12) | 20.4 |
|  | AzF neg. | n.a. | n.a. | 0 (0) | n.a. |
|  | Bpa | 6.52 ± 0.1 (4) | 6.47 ± 3.5 (4) | 2.2 ± 2.2 (5) | 14 |
|  | Bpa neg. | n.a. | n.a. | 0 (0) | n.a. |
|  | Se-AbK | n.a. | n.a. | 0 (0) | 6.2 |
|  | Se-AbK neg. | n.a. | n.a. | 0 (0) | n.a. |
| V16 | AzF^#^ | 6.60 (2) | 6.81 (2) | 0.82 ± 0.82 (5) | 5.2 |
|  | AzF neg. | n.a. | n.a. | 0 (0) | n.a. |
|  | Bpa | 6.58 ± 0.06 (7) | 5.95 ± 2.08 (7) | 3.60 ± 2.49 (7) | 12.2 |
|  | Bpa neg. | n.a. | n.a. | 0 (0) | n.a. |
|  | Se-AbK^#^ | 6.57 ± 0.03 (4) | 18.1 ± 13.38 (4) | 1.14 ± 0.63 (4) | 13.1 |
|  | Se-AbK neg. | n.a. | n.a. | 0 (0) | n.a. |
| S17 | AzF | n.a. | n.a. | 0 (0) | 4.7 |
|  | AzF neg. | n.a. | n.a. | 0 (0) | n.a. |
|  | Bpa | n.a. | n.a. | 0 (0) | 12.6 |
|  | Bpa neg. | n.a. | n.a. | 0 (0) | n.a. |
|  | Se-AbK | n.a. | n.a. | 0 (0) | 5.4 |
|  | Se-AbK neg. | n.a. | n.a. | 0 (0) | n.a. |
| I18 | AzF | 6.57 ± 0.07 (7) | 5.71 ± 1.92 (7) | 3.07 ± 1.47 (8) | 4.5 |
|  | AzF neg. | n.a. | n.a. | 0 (0) | n.a. |
|  | Bpa^#^ | 6.58 ± 0.08 (9) | 6.4 ± 1.6 (8) | 3.81 ± 1.48 (9) | 16.3 |
|  | Bpa neg. | n.a. | n.a. | 0 (0) | n.a. |
|  | Se-AbK | n.a. | n.a. | 0.14 (2) | 3.4 |
|  | Se-AbK neg. | n.a. | n.a. | 0 (0) | n.a. |
| Q19 | AzF^#^ | n.a. | n.a. | 0.39 ± 0.46 (3) | 3.6 |
|  | AzF neg. | n.a. | n.a. | 0 (0) | n.a. |
|  | Bpa^#^ | 6.60 (2) | 8.99 (2) | 2.19 ± 1.72 (4) | 10.2 |
|  | Bpa neg. | n.a. | n.a. | 0 (0) | n.a. |
|  | Se-AbK | n.a. | n.a. | 0 (0) | 5.3 |
|  | Se-AbK neg. | n.a. | n.a. | 0 (0) | n.a. |

| Position replaced by TAG | ncAA | mean pH_50_  ± S.D. (n) | mean n_H_  ± S.D. (n) | mean Imax (nA) ± S.D. (n) | average  TE (%) |
| --- | --- | --- | --- | --- | --- |
| A20 | AzF^#^ | n.a. | n.a. | 0.13 ± 0.13 (4) | 6.1 |
|  | AzF neg. | n.a. | n.a. | 0 (0) | n.a. |
|  | Bpa^#^ | 6.54 (1) | 6.54 (1) | 0.27 (2) | 17.2 |
|  | Bpa neg. | n.a. | n.a. | 0 (0) | n.a. |
|  | Se-AbK | n.a. | n.a. | 0 (0) | 6.3 |
|  | Se-AbK neg. | n.a. | n.a. | 0 (0) | n.a. |
| F21 | AzF | 6.57 ± 0.06 (5) | 7.13 ± 5.67 (5) | 3.47 ± 1.03 (5) | 15.6 |
|  | AzF neg. | n.a. | n.a. | 0 (0) | n.a. |
|  | Bpa^#^ | 6.56 ± 0.06 (3) | 4.96 (2) | 1.01 ± 1.08 (9) | 17.3 |
|  | Bpa neg. | n.a. | n.a. | 0 (0) | n.a. |
|  | Se-AbK | n.a. | n.a. | 0 (0) | 5.3 |
|  | Se-AbK neg. | n.a. | n.a. | 0 (0) | n.a. |
| A22 | AzF^#^ | n.a. | n.a. | 0.43 ± 0.25 (7) | 12.3 |
|  | AzF neg. | n.a. | n.a. | 0 (0) | n.a. |
|  | Bpa^#^ | 6.68 (1) | 7.28 (1) | 0.3 (2) | 9.6 |
|  | Bpa neg. | n.a. | n.a. | 0 (0) | n.a. |
|  | Se-AbK | n.a. | n.a. | 0 (0) | 8.1 |
|  | Se-AbK neg. | n.a. | n.a. | 0 (0) | n.a. |
| S23^#^ | AzF^#^ | 6.58 (1) | 26.2 (1) | 0.38 ± 0.21 (3) | 8.2 |
|  | AzF neg. | n.a. | n.a. | 0 (0) | n.a. |
|  | Bpa^#^ | n.a. | n.a. | 0.36 ± 0.45 (4) | 16.9 |
|  | Bpa neg. | n.a. | n.a. | 0 (0) | n.a. |
|  | Se-AbK^#^ | 6.56 ± 0.04 (4) | 16.7 ± 9.85 (4) | 0.46 ± 0.59 (6) | 15.1 |
|  | Se-AbK neg. | n.a. | n.a. | 0 (0) | n.a. |
| S24^#^ | AzF^#^ | 6.58 ± 0.03 (5) | 21.1 ± 6.56 (5) | 0.99 ± 1.08 (9) | 16 |
|  | AzF neg. | n.a. | n.a. | 0 (0) | n.a. |
|  | Bpa^#^ | 6.57 ± 0.03 (5) | 10.8 ± 8.04 (5) | 3.32 ± 1.26 (6) | 12.1 |
|  | Bpa neg. | n.a. | n.a. | 0 (0) | n.a. |
|  | Se-AbK^#^ | 6.60 ± 0.04 (9) | 19.4 ± 10.2 (9) | 3.02 ± 2.37 (11) | 13.7 |
|  | Se-AbK neg. | n.a. | n.a. | 0 (0) | n.a. |
| S25 | AzF | n.a. | n.a. | 0 (0) | 13.8 |
|  | AzF neg. | n.a. | n.a. | 0 (0) | n.a. |
|  | Bpa | n.a. | n.a. | 0 (0) | 16.6 |
|  | Bpa neg. | n.a. | n.a. | 0 (0) | n.a. |
|  | Se-AbK | n.a. | n.a. | 0 (0) | 6.2 |
|  | Se-AbK neg. | n.a. | n.a. | 0 (0) | n.a. |
| T26 | AzF | n.a. | n.a. | 0 (0) | 5.7 |
|  | AzF neg. | n.a. | n.a. | 0 (0) | n.a. |
|  | Bpa | n.a. | n.a. | 0 (0) | 12.6 |
|  | Bpa neg. | n.a. | n.a. | 0 (0) | n.a. |
|  | Se-AbK | n.a. | n.a. | 0 (0) | 6.7 |
|  | Se-AbK neg. | n.a. | n.a. | 0 (0) | n.a. |

| Position replaced by TAG | ncAA | mean pH_50_  ± S.D. (n) | mean n_H_  ± S.D. (n) | mean Imax (nA) ± S.D. (n) | average  TE (%) |
| --- | --- | --- | --- | --- | --- |
| L27 | AzF^#^ | n.a. | n.a. | 1.06 ± 1.03 (4) | 11.6 |
|  | AzF neg. | n.a. | n.a. | 0 (0) | n.a. |
|  | Bpa^#^ | n.a. | n.a. | 1.91 ± 3.03 (3) | 16.7 |
|  | Bpa neg. | n.a. | n.a. | 0.15 (1) | n.a. |
|  | Se-AbK | n.a. | n.a. | 0 (0) | 3.7 |
|  | Se-AbK neg. | n.a. | n.a. | 0 (0) | n.a. |
| H28 | AzF | n.a. | n.a. | 0 (0) | 11.6 |
|  | AzF neg. | n.a. | n.a. | 0 (0) | n.a. |
|  | Bpa | n.a. | n.a. | 0 (0) | 15.5 |
|  | Bpa neg. | n.a. | n.a. | 0 (0) | n.a. |
|  | Se-AbK | n.a. | n.a. | 0 (0) | 5.8 |
|  | Se-AbK neg. | n.a. | n.a. | 0 (0) | n.a. |
| G29 | AzF^#^ | 6.59 (2) | 19.9 (2) | 0.11 ± 0.04 (4) | 13.5 |
|  | AzF neg. | n.a. | n.a. | 0 (0) | n.a. |
|  | Bpa^#^ | 6.64 (1) | 6.14 (1) | 0.27 ± 0.11 (4) | 15.8 |
|  | Bpa neg. | n.a. | n.a. | 0 (0) | n.a. |
|  | Se-AbK | n.a. | n.a. | 0 (0) | 5.8 |
|  | Se-AbK neg. | n.a. | n.a. | 0 (0) | n.a. |
| L30 | AzF^#^ | 6.59 (2) | 6.16 (1) | 0.78 ± 1.19 (4) | 10.8 |
|  | AzF neg. | n.a. | n.a. | 0 (0) | n.a. |
|  | Bpa^#^ | 6.61 ± 0.07 (5) | 5.09 ± 1.89 (5) | 2.91 ± 1.68 (5) | 15.8 |
|  | Bpa neg. | n.a. | n.a. | 0 (0) | n.a. |
|  | Se-AbK | n.a. | n.a. | 0 (0) | 8.6 |
|  | Se-AbK neg. | n.a. | n.a. | 0 (0) | n.a. |
| A31^#^ | AzF^#^ | n.a. | n.a. | 0.19 ± 0.09 (5) | 13.1 |
|  | AzF neg. | n.a. | n.a. | 0 (0) | n.a. |
|  | Bpa^#^ | n.a. | n.a. | 0.23 ± 0.1 (3) | 17.8 |
|  | Bpa neg. | n.a. | n.a. | 0 (0) | n.a. |
|  | Se-AbK^#^ | 6.48 ± 0.09 (3) | 19.4 ± 12.2 (5) | 1.19 ± 2.06 (12) | 5.7 |
|  | Se-AbK neg. | n.a. | n.a. | 0 (0) | n.a. |
| H32 | AzF^#^ | n.a. | n.a. | 0.18 ± 0.08 (4) | 16.2 |
|  | AzF neg. | n.a. | n.a. | 0 (0) | n.a. |
|  | Bpa^#^ | n.a. | n.a. | 0.11 (2) | 17.6 |
|  | Bpa neg. | n.a. | n.a. | 0 (0) | n.a. |
|  | Se-AbK | n.a. | n.a. | 0 (0) | 11.3 |
|  | Se-AbK neg. | n.a. | n.a. | 0 (0) | n.a. |
| I33 | AzF^#^ | 6.62 (1) | 4.86 (1) | 1.21 (1) | 20.2 |
|  | AzF neg. | n.a. | n.a. | 0 (0) | n.a. |
|  | Bpa | n.a. | n.a. | 0 (0) | 17.4 |
|  | Bpa neg. | n.a. | n.a. | 0 (0) | n.a. |
|  | Se-AbK | n.a. | n.a. | 0 (0) | 9.4 |
|  | Se-AbK neg. | n.a. | n.a. | 0 (0) | n.a. |

| Position replaced by TAG | ncAA | mean pH_50_  ± S.D. (n) | mean n_H_  ± S.D. (n) | mean Imax (nA) ± S.D. (n) | average  TE (%) |
| --- | --- | --- | --- | --- | --- |
| F34 | AzF | 6.48 ± 0.06 (8) | 12.6 ± 9.94 (9) | 2.31 ± 1.41 (9) | 12.6 |
|  | AzF neg. | n.a. | n.a. | 0 (0) | n.a. |
|  | Bpa^#^ | 6.59 ± 0.07 (5) | 11.8 ± 10.4 (5) | 1.59 ± 1.55 (6) | 27.5 |
|  | Bpa neg. | n.a. | n.a. | 0 (0) | n.a. |
|  | Se-AbK | n.a. | n.a. | 0 (0) | 4.4 |
|  | Se-AbK neg. | n.a. | n.a. | 0 (0) | n.a. |
| S35 | AzF^#^ | 6.58 (1) | 29.6 (1) | 0.44 ± 0.53 (5) | 13.5 |
|  | AzF neg. | n.a. | n.a. | 0 (0) | n.a. |
|  | Bpa | n.a. | n.a. | 0 (0) | 28.9 |
|  | Bpa neg. | n.a. | n.a. | 0 (0) | n.a. |
|  | Se-AbK^#^ | 6.48 ± 0.18 (3) | 6.67 ± 4.28 (3) | 0.37 ± 0.2 (6) | 16.3 |
|  | Se-AbK neg. | n.a. | n.a. | 0 (0) | n.a. |
| Y36 | AzF^#^ | n.a. | n.a. | 0.17 ± 0.12 (5) | 11 |
|  | AzF neg. | n.a. | n.a. | 0 (0) | n.a. |
|  | Bpa^#^ | 6.72 ± 0.03 (3) | 4.32 ± 1.47 (3) | 0.66 ± 0.54 (4) | 14 |
|  | Bpa neg. | n.a. | n.a. | 0 (0) | n.a. |
|  | Se-AbK | n.a. | n.a. | 0 (0) | 7.2 |
|  | Se-AbK neg. | n.a. | n.a. | 0 (0) | n.a. |
| E37 | AzF^#^ | n.a. | n.a. | 0.4 (2) | 15.1 |
|  | AzF neg. | n.a. | n.a. | 0 (0) | n.a. |
|  | Bpa^#^ | 6.75 (2) | 37.3 (2) | 0.61 ± 1.03 (4) | 17.2 |
|  | Bpa neg. | n.a. | n.a. | 0 (0) | n.a. |
|  | Se-AbK | n.a. | n.a. | 0 (0) | 12 |
|  | Se-AbK neg. | n.a. | n.a. | 0 (0) | n.a. |
| R38 | AzF^#^ | n.a. | n.a. | 0.54 ± 0.63 (4) | 18.4 |
|  | AzF neg. | n.a. | n.a. | 0 (0) | n.a. |
|  | Bpa^#^ | 6.65 (1) | 23.7 (1) | 0.29 ± 0.21 (3) | 26.9 |
|  | Bpa neg. | n.a. | n.a. | 0 (0) | n.a. |
|  | Se-AbK | n.a. | n.a. | 0 (0) | 13.3 |
|  | Se-AbK neg. | n.a. | n.a. | 0 (0) | n.a. |
| L39 | AzF | 6.44 ± 0.08 (16) | 5.92 ± 2.96 (16) | 3.2 ± 1.66 (16) | 9.2 |
|  | AzF neg. | 6.44 (1) | 3.49 (1) | 0.57 (1) | n.a. |
|  | Bpa^#^ | 6.5 ± 0.11 (5) | 10.3 ± 12.5 (4) | 1.73 ± 1.48 (5) | 17.2 |
|  | Bpa neg. | n.a. | n.a. | 0 (0) | n.a. |
|  | Se-AbK | 6.45 ± 0.14 (8) | 7.87 ± 5.25 (10) | 1.39 ± 0.93 (10) | 11.7 |
|  | Se-AbK neg. | n.a. | n.a. | 0 (0) | n.a. |
| S40 | AzF | 6.49 ± 0.11 (10) | 7.84 ± 9.64 (11) | 3.54 ± 1.51 (11) | 10.1 |
|  | AzF neg. | 6.36 ± 0.19 (3) | 14.0 ± 16.6 (3) | 0.6 ± 0.68 (3) | n.a. |
|  | Bpa | 6.63 ± 0.05 (3) | 15.9 ± 16.7 (3) | 4.01 ± 1.75 (3) | 27.3 |
|  | Bpa neg.^#^ | 6.42 ± 0.04 (3) | 5.97 ± 0.57 (3) | 0.48 ± 0.49 (3) | n.a. |
|  | Se-AbK | 6.45 ± 0.1 (5) | 11.2 ± 8.07 (6) | 1.91 ± 0.99 (7) | 12.9 |
|  | Se-AbK neg. | 6.42 ± 0.01 (3) | 4.19 ± 1.07 (3) | 0.1 ± 0.02 (3) | n.a. |

| Position replaced by TAG | ncAA | mean pH_50_  ± S.D. (n) | mean n_H_  ± S.D. (n) | mean Imax (nA) ± S.D. (n) | average  TE (%) |
| --- | --- | --- | --- | --- | --- |
| L41 | AzF | 6.48 ± 0.07 (13) | 5.03 ± 1.61 (13) | 3.94 ± 1.69 (13) | 15.1 |
|  | AzF neg. | n.a. | n.a. | 0.18 (1) | n.a. |
|  | Bpa^#^ | 6.62 ± 0.04 (4) | 9.98 ± 6.8 (4) | 3.31 ± 3.08 (4) | 27.6 |
|  | Bpa neg. ^#^ | 6.49 (2) | 6.41 (2) | 2.03 (2) | n.a. |
|  | Se-AbK | 6.51 ± 0.18 (6) | 6.44 ± 5.76 (7) | 1.61 ± 1.36 (7) | 11.2 |
|  | Se-AbK neg. | n.a. | n.a. | 0 (0) | n.a. |
| K42 | AzF | n.a. | n.a. | 0 (0) | 13.4 |
|  | AzF neg. | n.a. | n.a. | 0 (0) | n.a. |
|  | Bpa | n.a. | n.a. | 0 (0) | 12.4 |
|  | Bpa neg. | n.a. | n.a. | 0 (0) | n.a. |
|  | Se-AbK^#^ | 6.58 ± 0.08 (4) | 8.34 ± 5.15 (4) | 1.91 ± 1.5 (4) | 2 |
|  | Se-AbK neg. | n.a. | n.a. | 0 (0) | n.a. |
| R43 | AzF | n.a. | n.a. | 0 (0) | 9.2 |
|  | AzF neg. | n.a. | n.a. | 0 (0) | n.a. |
|  | Bpa | n.a. | n.a. | 0 (0) | 7.4 |
|  | Bpa neg. | n.a. | n.a. | 0 (0) | n.a. |
|  | Se-AbK | n.a. | n.a. | 0 (0) | 7.2 |
|  | Se-AbK neg. | n.a. | n.a. | 0 (0) | n.a. |
| A44 | AzF | 6.56 ± 0.2 (3) | 10.4 ± 9.48 (4) | 7.65 ± 4.45 (4) | 15.1 |
|  | AzF neg. | n.a. | n.a. | 0 (0) | n.a. |
|  | Bpa | 6.6 ± 0.05 (6) | 5.8 ± 1.9 (6) | 3.9 ± 2.04 (6) | 14.8 |
|  | Bpa neg. | 6.26 (1) | 2.24 (1) | 3.41 (1) | n.a. |
|  | Se-AbK^#^ | 6.52 ± 0.04 (3) | 15.5 ± 9.84 (5) | 1.19 ± 0.38 (6) | 16.1 |
|  | Se-AbK neg. | n.a. | n.a. | 0 (0) | n.a. |
| L45 | AzF^#^ | 6.74 ± 0.1 (7) | 6.67 ± 2.76 (7) | 5.31 ± 1.91 (7) | 11.3 |
|  | AzF neg. | n.a. | n.a. | 0 (0) | n.a. |
|  | Bpa | 6.58 ± 0.05 (5) | 6.05 ± 4.19 (5) | 2.25 ± 0.52 (5) | 12.8 |
|  | Bpa neg. | n.a. | n.a. | 0 (0) | n.a. |
|  | Se-AbK | 6.35 ± 0.15 (8) | 4.47 ± 1.51 (8) | 2.42 ± 3.09 (8) | 16.5 |
|  | Se-AbK neg. | n.a. | n.a. | 0 (0) | n.a. |
| W46 | AzF | n.a. | n.a. | 0 (0) | 13.9 |
|  | AzF neg. | n.a. | n.a. | 0 (0) | n.a. |
|  | Bpa | n.a. | n.a. | 0 (0) | 14 |
|  | Bpa neg. | n.a. | n.a. | 0 (0) | n.a. |
|  | Se-AbK | n.a. | n.a. | 0 (0) | 15.3 |
|  | Se-AbK neg. | n.a. | n.a. | 0 (0) | n.a. |
| A47^#^ | AzF^#^ | 6.46 ± 0.2 (3) | 8.34 (1) | 2.49 ± 1.81 (8) | 15.9 |
|  | AzF neg. | n.a. | n.a. | 0 (0) | n.a. |
|  | Bpa^#^ | 6.38 (2) | 8.88 (1) | 0.46 ± 0.34 (6) | 23 |
|  | Bpa neg. | n.a. | n.a. | 0.31 (1) | n.a. |
|  | Se-AbK | n.a. | n.a. | 0 (0) | 15.2 |
|  | Se-AbK neg. | n.a. | n.a. | 0 (0) | n.a. |

| Position replaced by TAG | ncAA | mean pH_50_  ± S.D. (n) | mean n_H_  ± S.D. (n) | mean Imax (nA) ± S.D. (n) | average  TE (%) |
| --- | --- | --- | --- | --- | --- |
| L48 | AzF | 6.56 ± 0.03 (3) | 7.72 ± 7.92 (3) | 5.9 ± 2.37 (3) | 12.8 |
|  | AzF neg. | n.a. | n.a. | 0 (0) | n.a. |
|  | Bpa | 6.57 ± 0.09 (5) | 7.45 ± 2.21 (5) | 3.38 ± 1.02 (5) | 18.2 |
|  | Bpa neg. | n.a. | n.a. | 0.65 (2) | n.a. |
|  | Se-AbK | 6.51 ± 0.1 (9) | 9.95 ± 7.87 (10) | 1.14 ± 1.29 (12) | 15.5 |
|  | Se-AbK neg. | n.a. | n.a. | 0 (0) | n.a. |
| V65 | AzF | n.a. | n.a. | 0 (0) | 12.6 |
|  | AzF neg. | n.a. | n.a. | 0 (0) | n.a. |
|  | Bpa | 6.54 ± 0.07 (10) | 5.02 ± 1.46 (10) | 2.16 ± 1.46 (10) | 15.3 |
|  | Bpa neg. | n.a. | n.a. | 0 (0) | n.a. |
|  | Se-AbK^#^ | n.a. | n.a. | 0.49 ± 0.05 (3) | 15.8 |
|  | Se-AbK neg. | n.a. | n.a. | 0 (0) | n.a. |
| Q66 | AzF | 6.63 ± 0.14 (3) | 5.95 ± 3.13 (3) | 2.15 ± 0.58 (3) | 12.7 |
|  | AzF neg. | n.a. | n.a. | 0 (0) | n.a. |
|  | Bpa | 6.19 (1) | 5.79 (1) | 3.39 (2) | 11.6 |
|  | Bpa neg. | n.a. | n.a. | 0 (0) | n.a. |
|  | Se-AbK^#^ | 6.51 ± 0.03 (5) | 4.82 ± 2.79 (5) | 0.74 ± 0.85 (9) | 12.2 |
|  | Se-AbK neg. | n.a. | n.a. | 0 (0) | n.a. |
| Y67 | AzF | n.a. | n.a. | 0 (0) | 11.6 |
|  | AzF neg. | n.a. | n.a. | 0 (0) | n.a. |
|  | Bpa | 6.33 ± 0.09 (6) | 6.13 ± 1.15 (6) | 4.55 ± 1.40 (6) | 18.5 |
|  | Bpa neg. | n.a. | n.a. | 0 (0) | n.a. |
|  | Se-AbK^#^ | 6.43 ± 0.16 (8) | 15.2 ± 10.3 (9) | 2.63 ± 3.81 (9) | 5.3 |
|  | Se-AbK neg. | n.a. | n.a. | 0 (0) | n.a. |
| Y68 | AzF | n.a. | n.a. | 0 (0) | 10.0 |
|  | AzF neg. | n.a. | n.a. | 0 (0) | n.a. |
|  | Bpa^#^ | 6.52 ± 0.12 (4) | 14.2 ± 9.48 (4) | 2.66 ± 1.68 (4) | 17.8 |
|  | Bpa neg. | n.a. | n.a. | 0 (0) | n.a. |
|  | Se-AbK | 6.23 ± 0.22 (3) | 2.61 ± 1.01 (3) | 0.16 ± 0.05 (3) | 6.8 |
|  | Se-AbK neg. | n.a. | n.a. | 0 (0) | n.a. |
| F69 | AzF | 6.6 ± 0.09 (19) | 9.43 ± 8.98 (22) | 4.22 ± 2.25 (22) | 12.2 |
|  | AzF neg. | n.a. | n.a. | 0 (0) | n.a. |
|  | Bpa | 6.74 ± 0.08 (5) | 13.4 ± 13.6 (5) | 2.67 ± 1.42 (5) | 10.3 |
|  | Bpa neg. | n.a. | n.a. | 0 (0) | n.a. |
|  | Se-AbK^#^ | 6.44 ± 0.05 (4) | 7.69 ± 0.52 (4) | 2.36 ± 1.84 (4) | 5.6 |
|  | Se-AbK neg. | n.a. | n.a. | 0 (0) | n.a. |
| Y71 | AzF | 6.64 ± 0.1 (12) | 9.71 ± 8.17 (12) | 3.77 ± 2.00 (12) | 12.8 |
|  | AzF neg. | 6.47 ± 0.16 (7) | 2.49 ± 1.1 (7) | 1.12 ± 1.04 (7) | n.a. |
|  | Bpa^#^ | 6.61 ± 0.15 (3) | 6.55 (2) | 2.97 ± 1.32 (3) | 11.5 |
|  | Bpa neg. | n.a. | n.a. | 0 (0) | n.a. |
|  | Se-AbK | 6.3 ± 0.14 (9) | 2.74 ± 1.44 (9) | 1.04 ± 1.36 (9) | 5.8 |
|  | Se-AbK neg. | n.a. | n.a. | 0 (0) | n.a. |

| Position replaced by TAG | ncAA | mean pH_50_  ± S.D. (n) | mean n_H_  ± S.D. (n) | mean Imax (nA) ± S.D. (n) | average  TE (%) |
| --- | --- | --- | --- | --- | --- |
| R175 | AzF | n.a. | n.a. | 0 (0) | 9.0 |
|  | AzF neg. | n.a. | n.a. | 0 (0) | n.a. |
|  | Bpa | n.a. | n.a. | 0 (0) | 13.0 |
|  | Bpa neg. | n.a. | n.a. | 0 (0) | n.a. |
|  | Se-AbK^#^ | 6.21 ± 0.06 (12) | 6.65 ± 8.52 (13) | 0.93 ± 1.13 (15) | 13.3 |
|  | Se-AbK neg. | n.a. | n.a. | 0 (0) | n.a. |
| G176 | AzF | n.a. | n.a. | 0 (0) | 9.5 |
|  | AzF neg. | n.a. | n.a. | 0 (0) | n.a. |
|  | Bpa | n.a. | n.a. | 0 (0) | 28.9 |
|  | Bpa neg. | n.a. | n.a. | 0 (0) | n.a. |
|  | Se-AbK | n.a. | n.a. | 0 (0) | 15 |
|  | Se-AbK neg. | n.a. | n.a. | 0 (0) | n.a. |
| E177 | AzF | 6.59 ± 0.11 (3) | 6.29 ± 3.53 (3) | 1.07 ± 0.47 (3) | 8.7 |
|  | AzF neg. | n.a. | n.a. | 0 (0) | n.a. |
|  | Bpa | n.a. | n.a. | 0 (0) | 13.4 |
|  | Bpa neg. | n.a. | n.a. | 0 (0) | n.a. |
|  | Se-AbK^#^ | 6.49 ± 0.08 (5) | 15.7 ± 11.2 (5) | 1.26 ± 1.23 (5) | 7.7 |
|  | Se-AbK neg. | n.a. | n.a. | 0 (0) | n.a. |
| T236 | AzF | 6.17 ± 0.14 (10) | 15.7 ± 14.8 (10) | 1.47 ± 1.1 (10) | 12.0 |
|  | AzF neg. | n.a. | n.a. | 0 (0) | n.a. |
|  | Bpa | 6.17 ± 0.08 (12) | 9.85 ± 10.7 (12) | 1.24 ± 0.89 (14) | 18.3 |
|  | Bpa neg. | n.a. | n.a. | 0 (0) | n.a. |
|  | Se-AbK | 6.25 ± 0.07 (8) | 13.5 ± 13.2 (8) | 1.60 ± 1.10 (8) | 11.8 |
|  | Se-AbK neg. | n.a. | n.a. | 0 (0) | n.a. |
| T239 | AzF | 6.05 ± 0.12 (12) | 3.82 ± 1.38 (13) | 0.83 ± 0.84 (14) | 10.7 |
|  | AzF neg. | n.a. | n.a. | 0 (0) | n.a. |
|  | Bpa | 5.49 ± 0.13 (6) | 2.61 ± 0.62 (6) | 1.16 ± 1.17 (6) | 16.3 |
|  | Bpa neg. | n.a. | n.a. | 0 (0) | n.a. |
|  | Se-AbK | n.a. | n.a. | 0 (0) | 13.6 |
|  | Se-AbK neg. | n.a. | n.a. | 0 (0) | n.a. |
| P286 | AzF | n.a. | n.a. | 0 (0) | 8.8 |
|  | AzF neg. | n.a. | n.a. | 0 (0) | n.a. |
|  | Bpa | n.a. | n.a. | 0 (0) | 19.8 |
|  | Bpa neg. | n.a. | n.a. | 0 (0) | n.a. |
|  | Se-AbK | n.a. | n.a. | 0 (0) | 12.4 |
|  | Se-AbK neg. | n.a. | n.a. | 0 (0) | n.a. |
| W287 | AzF | 6.63 ± 0.1 (12) | 8.54 ± 7.35 (12) | 3.07 ± 1.75 (13) | 11.5 |
|  | AzF neg. | n.a. | n.a. | 0 (0) | n.a. |
|  | Bpa | n.a. | n.a. | 0 (0) | 14.9 |
|  | Bpa neg. | n.a. | n.a. | 0 (0) | n.a. |
|  | Se-AbK | n.a. | n.a. | 0 (0) | 12.9 |
|  | Se-AbK neg. | n.a. | n.a. | 0 (0) | n.a. |

| Position replaced by TAG | ncAA | mean pH_50_  ± S.D. (n) | mean n_H_  ± S.D. (n) | mean Imax (nA) ± S.D. (n) | average  TE (%) |
| --- | --- | --- | --- | --- | --- |
| K343^#^ | AzF^#^ | 6.46 ± 0.09 (6) | 12.0 ± 12.9 (6) | 0.63 ± 0.43 (7) | 11.9 |
|  | AzF neg. | n.a. | n.a. | 0 (0) | n.a. |
|  | Bpa^#^ | 6.19 ± 0.04 (6) | 4.65 ± 0.96 (6) | 1.89 ± 1.26 (6) | 20.4 |
|  | Bpa neg. | n.a. | n.a. | 0 (0) | n.a. |
|  | Se-AbK^#^ | 6.32 (2) | 29.7 (2) | 0.51 (2) | 12 |
|  | Se-AbK neg. | n.a. | n.a. | 0 (0) | n.a. |
| E344 | AzF | 6.6 ± 0.05 (5) | 17.3 ± 8.73 (5) | 1.18 ± 1.55 (5) | 9.2 |
|  | AzF neg. | n.a. | n.a. | 0 (0) | n.a. |
|  | Bpa | 6.42 ± 0.05 (4) | 8.67 ± 6.19 (5) | 1.62 ± 1.34 (5) | 20.8 |
|  | Bpa neg. | n.a. | n.a. | 0.5 ± 0.35 (3) | n.a. |
|  | Se-AbK | 6.43 ± 0.14 (6) | 13.2 ± 11.7 (7) | 0.20 ± 0.09 (7) | 8.2 |
|  | Se-AbK neg. | n.a. | n.a. | 0 (0) | n.a. |
| P348 | AzF | n.a. | n.a. | 0 (0) | 10.8 |
|  | AzF neg. | n.a. | n.a. | 0 (0) | n.a. |
|  | Bpa | n.a. | n.a. | 0 (0) | 20.1 |
|  | Bpa neg. | n.a. | n.a. | 0 (0) | n.a. |
|  | Se-AbK | n.a. | n.a. | 0 (0) | 7.0 |
|  | Se-AbK neg. | n.a. | n.a. | 0 (0) | n.a. |
| D351 | AzF^#^ | 6.23 ± 0.06 (7) | 10.1 ± 10.4 (7) | 0.84 ± 0.77 (8) | 10.9 |
|  | AzF neg. | n.a. | n.a. | 0 (0) | n.a. |
|  | Bpa | 6.17 ± 0.12 (3) | 5.52 ± 1.46 (3) | 0.24 ± 0.24 (3) | 17.8 |
|  | Bpa neg. | n.a. | n.a. | 0 (0) | n.a. |
|  | Se-AbK | 6.33 ± 0.06 (9) | 6.72 ± 7.76 (10) | 0.14 ± 0.15 (10) | 12.0 |
|  | Se-AbK neg. | n.a. | n.a. | 0 (0) | n.a. |
| E355^#^ | AzF^#^ | 6.46 ± 0.11 (8) | 4.24 ± 1.69 (8) | 1.5 ± 1.91 (10) | 12.6 |
|  | AzF neg. | n.a. | n.a. | 0 (0) | n.a. |
|  | Bpa^#^ | 6.53 ± 0.1 (11) | 6.51 ± 8.43 (11) | 1.33 ± 1.52 (14) | 17.0 |
|  | Bpa neg. | n.a. | n.a. | 0 (0) | n.a. |
|  | Se-AbK^#^ | 6.87 ± 0.22 (3) | 21.5 (2) | 0.77 ± 0.73 (4) | 11.9 |
|  | Se-AbK neg. | n.a. | n.a. | 0 (0) | n.a. |
| K356 | AzF | 6.18 ± 0.22 (8) | 9.18 ± 9.19 (8) | 2.37 ± 2.23 (8) | 10.4 |
|  | AzF neg. | n.a. | n.a. | 0 (0) | n.a. |
|  | Bpa | 6.07 ± 0.28 (8) | 11.2 ± 13.4 (8) | 1.45 ± 1.42 (8) | 16.8 |
|  | Bpa neg. | n.a. | n.a. | 0.46 (1) | n.a. |
|  | Se-AbK^#^ | 6.26 ± 0.22 (6) | 10.2 ± 12.7 (6) | 0.73 ± 0.84 (7) | 9.8 |
|  | Se-AbK neg. | n.a. | n.a. | 0 (0) | n.a. |
| D357 | AzF | 5.66 ± 0.26 (10) | 2.51 ± 0.57 (10) | 1.28 ± 1.3 (11) | 13.0 |
|  | AzF neg. | n.a. | n.a. | 0 (0) | n.a. |
|  | Bpa | 5.91 ± 0.1 (8) | 2.85 ± 0.65 (8) | 1.84 ± 2.02 (10) | 16.8 |
|  | Bpa neg. | n.a. | n.a. | 0 (0) | n.a. |
|  | Se-AbK | 5.81 ± 0.17 (6) | 2.19 ± 0.38 (6) | 0.35 ± 0.43 (6) | 11.4 |
|  | Se-AbK neg. | n.a. | n.a. | 0 (0) | n.a. |

| Position replaced by TAG | ncAA | mean pH_50_  ± S.D. (n) | mean n_H_  ± S.D. (n) | mean Imax (nA) ± S.D. (n) | average  TE (%) |
| --- | --- | --- | --- | --- | --- |
| Y426 | AzF | n.a. | n.a. | 0 (0) | 8.2 |
|  | AzF neg. | n.a. | n.a. | 0 (0) | n.a. |
|  | Bpa | n.a. | n.a. | 0 (0) | 33.5 |
|  | Bpa neg. | n.a. | n.a. | 0 (0) | n.a. |
|  | Se-AbK^#^ | 6.43 ± 0.09 (4) | 6.37 ± 4.49 (4) | 0.68 ± 0.94 (5) | 9.5 |
|  | Se-AbK neg. | n.a. | n.a. | 0 (0) | n.a. |
| E427 | AzF | n.a. | n.a. | 0 (0) | 10.9 |
|  | AzF neg. | n.a. | n.a. | 0 (0) | n.a. |
|  | Bpa | n.a. | n.a. | 0 (0) | 20.1 |
|  | Bpa neg. | n.a. | n.a. | 0 (0) | n.a. |
|  | Se-AbK | n.a. | n.a. | 0 (0) | 7.6 |
|  | Se-AbK neg. | n.a. | n.a. | 0 (0) | n.a. |
| I428 | AzF^#^ | 6.48 ± 0.14 (4) | 18.3 ± 15.1 (3) | 1.11 ± 0.82 (6) | 10.5 |
|  | AzF neg. | n.a. | n.a. | 0 (0) | n.a. |
|  | Bpa | n.a. | n.a. | 0 (0) | 25.3 |
|  | Bpa neg. | n.a. | n.a. | 0 (0) | n.a. |
|  | Se-AbK | n.a. | n.a. | 0 (0) | 6.4 |
|  | Se-AbK neg. | n.a. | n.a. | 0 (0) | n.a. |
| A429 | AzF | n.a. | n.a. | 0 (0) | 12.3 |
|  | AzF neg. | n.a. | n.a. | 0 (0) | n.a. |
|  | Bpa | n.a. | n.a. | 0 (0) | 7.8 |
|  | Bpa neg. | n.a. | n.a. | 0 (0) | n.a. |
|  | Se-AbK | n.a. | n.a. | 0 (0) | 6.7 |
|  | Se-AbK neg. | n.a. | n.a. | 0 (0) | n.a. |
| G430 | AzF | n.a. | n.a. | 0 (0) | 11.6 |
|  | AzF neg. | n.a. | n.a. | 0 (0) | n.a. |
|  | Bpa | n.a. | n.a. | 0 (0) | 7.7 |
|  | Bpa neg. | n.a. | n.a. | 0 (0) | n.a. |
|  | Se-AbK | 6.46 ± 0.13 (6) | 10.6 ± 13.3 (6) | 0.18 ± 0.24 (7) | 5.7 |
|  | Se-AbK neg. | n.a. | n.a. | 0 (0) | n.a. |
| F442 | AzF^#^ | 6.62 ± 0.06 (7) | 10.9 ± 6.8 (7) | 3.05 ± 2.5 (7) | 7.5 |
|  | AzF neg. | n.a. | n.a. | 0 (0) | n.a. |
|  | Bpa | 6.64 ± 0.03 (4) | 1.73 ± 6.19 (4) | 1.32 ± 1.4 (4) | 11.9 |
|  | Bpa neg. | n.a. | n.a. | 0 (0) | n.a. |
|  | Se-AbK | n.a. | n.a. | 0 (0) | 4.8 |
|  | Se-AbK neg. | n.a. | n.a. | 0 (0) | n.a. |
| F454 | AzF | n.a. | n.a. | 0 (0) | 9.4 |
|  | AzF neg. | n.a. | n.a. | 0 (0) | n.a. |
|  | Bpa^#^ | 6.59 ± 0.06 (4) | 7.97 ± 1.72 (4) | 2.51 ± 1.79 (4) | 12.7 |
|  | Bpa neg. | n.a. | n.a. | 0 (0) | n.a. |
|  | Se-AbK | n.a. | n.a. | 0 (0) | 7.4 |
|  | Se-AbK neg. | n.a. | n.a. | 0 (0) | n.a. |

| Position replaced by TAG | ncAA | mean pH_50_  ± S.D. (n) | mean n_H_  ± S.D. (n) | mean Imax (nA) ± S.D. (n) | average  TE (%) |
| --- | --- | --- | --- | --- | --- |
| D455 | AzF | 6.56 ± 0.14 (7) | 6.81 ± 2.44 (7) | 0.83 ± 0.82 (8) | 9.7 |
|  | AzF neg. | n.a. | n.a. | 0 (0) | n.a. |
|  | Bpa^#^ | 6.46 ± 0.05 (4) | 5.82 ± 2.38 (4) | 0.91 ± 0.88 (4) | 13.9 |
|  | Bpa neg. | n.a. | n.a. | 0 (0) | n.a. |
|  | Se-AbK | n.a. | n.a. | 0 (0) | 9.4 |
|  | Se-AbK neg. | n.a. | n.a. | 0 (0) | n.a. |
| Y456 | AzF^#^ | 6.55 ± 0.06 (4) | 13.8 ± 8.49 (4) | 0.9 ± 1.07 (5) | 7.1 |
|  | AzF neg. | n.a. | n.a. | 0 (0) | n.a. |
|  | Bpa^#^ | 6.41 (1) | 7.66 (1) | 0.56 ± 0.47 (3) | 14.8 |
|  | Bpa neg. | n.a. | n.a. | 0 (0) | n.a. |
|  | Se-AbK | n.a. | n.a. | 0 (0) | 9.8 |
|  | Se-AbK neg. | n.a. | n.a. | 0 (0) | n.a. |
| Y458 | AzF^#^ | 6.54 ± 0.08 (5) | 17.5 ± 12.3 (5) | 2.48 ± 1.61 (6) | 9.9 |
|  | AzF neg. | n.a. | n.a. | 0 (0) | n.a. |
|  | Bpa | 6.46 ± 0.12 (13) | 6.22 ± 1.52 (13) | 1.86 ± 2.2 (13) | 10.1 |
|  | Bpa neg. | n.a. | n.a. | 0 (0) | n.a. |
|  | Se-AbK | 6.44 ± 0.03 (4) | 4.63 ± 2.28 (4) | 0.26 ± 0.27 (4) | 10.0 |
|  | Se-AbK neg. | n.a. | n.a. | 0 (0) | n.a. |
| E459 | AzF | n.a. | n.a. | 0 (0) | 6 |
|  | AzF neg. | n.a. | n.a. | 0 (0) | n.a. |
|  | Bpa | n.a. | n.a. | 0 (0) | 8 |
|  | Bpa neg. | n.a. | n.a. | 0 (0) | n.a. |
|  | Se-AbK | n.a. | n.a. | 0 (0) | 5.5 |
|  | Se-AbK neg. | n.a. | n.a. | 0 (0) | n.a. |
| V460 | AzF | n.a. | n.a. | 0 (0) | 5.3 |
|  | AzF neg. | n.a. | n.a. | 0 (0) | n.a. |
|  | Bpa | n.a. | n.a. | 0 (0) | 10.4 |
|  | Bpa neg. | n.a. | n.a. | 0 (0) | n.a. |
|  | Se-AbK | n.a. | n.a. | 0 (0) | 3.7 |
|  | Se-AbK neg. | n.a. | n.a. | 0 (0) | n.a. |
| I461 | AzF^#^ | 6.47 ± 0.13 (5) | 3.93 ± 1.53 (4) | 0.97 ± 0.88 (5) | 6.9 |
|  | AzF neg. | n.a. | n.a. | 0 (0) | n.a. |
|  | Bpa^#^ | 6.59 ± 0.06 (3) | 11.8 ± 6.01 (3) | 1.97 ± 1.74 (3) | 14.0 |
|  | Bpa neg. | n.a. | n.a. | 0 (0) | n.a. |
|  | Se-AbK | n.a. | n.a. | 0 (0) | 5.0 |
|  | Se-AbK neg. | n.a. | n.a. | 0 (0) | n.a. |
| K462 | AzF^#^ | 6.56 ± 0.13 (3) | 6.13 (2) | 3.37 ± 1.87 (4) | 7.6 |
|  | AzF neg. | n.a. | n.a. | 0 (0) | n.a. |
|  | Bpa | 6.63 (2) | 6.4 (2) | 5.12 (2) | 9.6 |
|  | Bpa neg. | n.a. | n.a. | 0 (0) | n.a. |
|  | Se-AbK | n.a. | n.a. | 0 (0) | 4.8 |
|  | Se-AbK neg. | n.a. | n.a. | 0 (0) | n.a. |

| Position replaced by TAG | ncAA | mean pH_50_  ± S.D. (n) | mean n_H_  ± S.D. (n) | mean Imax (nA) ± S.D. (n) | average  TE (%) |
| --- | --- | --- | --- | --- | --- |
| H463 | AzF^#^ | 6.53 ± 0.09 (3) | 15.8 ± 17.0 (3) | 3.27 ± 1.47 (4) | 8.0 |
|  | AzF neg. | n.a. | n.a. | 0 (0) | n.a. |
|  | Bpa | n.a. | n.a. | 0 (0) | 7.6 |
|  | Bpa neg. | n.a. | n.a. | 0 (0) | n.a. |
|  | Se-AbK | n.a. | n.a. | 0 (0) | 13.0 |
|  | Se-AbK neg. | n.a. | n.a. | 0 (0) | n.a. |
| K464 | AzF^#^ | 6.40 ± 0.16 (5) | 12.6 ± 5.74 (3) | 1.56 ± 0.96 (7) | 8.4 |
|  | AzF neg. | n.a. | n.a. | 0 (0) | n.a. |
|  | Bpa | n.a. | n.a. | 0 (0) | 7.8 |
|  | Bpa neg. | n.a. | n.a. | 0 (0) | n.a. |
|  | Se-AbK | n.a. | n.a. | 0 (0) | 11.9 |
|  | Se-AbK neg. | n.a. | n.a. | 0 (0) | n.a. |
| L465 | AzF^#^ | 6.5 ± 0.12 (4) | 23.3 ± 6.24 (4) | 0.91 ± 0.93 (6) | 6.5 |
|  | AzF neg. | n.a. | n.a. | 0 (0) | n.a. |
|  | Bpa | n.a. | n.a. | 0 (0) | 5.6 |
|  | Bpa neg. | n.a. | n.a. | 0 (0) | n.a. |
|  | Se-AbK | n.a. | n.a. | 0 (0) | 16.3 |
|  | Se-AbK neg. | n.a. | n.a. | 0 (0) | n.a. |
| C466 | AzF | n.a. | n.a. | 0 (0) | 5.8 |
|  | AzF neg. | n.a. | n.a. | 0 (0) | n.a. |
|  | Bpa^#^ | 6.69 (2) | 29.7 (1) | 2.31 (2) | 6.6 |
|  | Bpa neg. ^#^ | 6.76 ± 0.07 (3) | 11.2 ± 11.4 (3) | 0.98 ± 0.59 (3) | n.a. |
|  | Se-AbK^#^ | 6.46 (1) | 4.05 (1) | 0.41 ± 0.16 (3) | 12.4 |
|  | Se-AbK neg.^#^ | 6.24 ± 0.09 (3) | 10.5 ± 9.44 (3) | 2.29 ± 1.12 (6) | n.a. |
| R467 | AzF | 6.74 ± 0.05 (4) | 4.9 ± 1.55 (5) | 4.97 ± 1.16 (5) | 8.2 |
|  | AzF neg. | 6.64 ± 0.02 (5) | 5.7 ± 2.76 (5) | 6.15 ± 3.24 (5) | n.a. |
|  | Bpa | 6.75 ± 0.04 (6) | 6.26 ± 0.82 (6) | 5.62 ± 1.86 (6) | 6.4 |
|  | Bpa neg. ^#^ | 6.69 (1) | 6.35 (1) | 4.78 (1) | n.a. |
|  | Se-AbK^#^ | 6.51 ± 0.17 (4) | 6.46 ± 0.76 (3) | 2.69 ± 1.77 (5) | 8.1 |
|  | Se-AbK neg. | 6.61 ± 0.03 (3) | 5.68 ± 2.69 (3) | 4.34 ± 1.58 (3) | n.a. |
| R468 | AzF^#^ | 6.72 ± 0.11 (6) | 7.01 ± 0.54 (6) | 4.07 ± 1.13 (6) | 10.0 |
|  | AzF neg. | 6.71 ± 0.04 (7) | 7.71 ± 0.9 (7) | 7.22 ± 2.16 (7) | n.a. |
|  | Bpa | 6.73 ± 0.1 (7) | 7.87 ± 6.96 (7) | 4.95 ± 2.05 (8) | 6.8 |
|  | Bpa neg. | 6.76 (2) | 6.11 (2) | 4.91 (2) | n.a. |
|  | Se-AbK | 6.51 ± 0.12 (5) | 9.53 ± 6.54 (5) | 3.42 ± 2.44 (5) | 10.2 |
|  | Se-AbK neg. | 6.53 ± 0.1 (4) | 12.4 ± 12.8 (5) | 3.97 ± 2.27 (5) | n.a. |
| G469 | AzF | 6.75 ± 0.13 (6) | 8.51 ± 4.49 (6) | 5.16 ± 1.77 (6) | 7.3 |
|  | AzF neg. | 6.74 ± 0.06 (5) | 6.77 ± 2.16 (5) | 7.01 ± 2.36 (5) | n.a. |
|  | Bpa | 6.78 ± 0.04 (3) | 6.04 ± 0.8 (3) | 5.91 ± 1.62 (3) | 7.4 |
|  | Bpa neg. ^#^ | 6.71 (1) | 7.63 (1) | 4.72 (1) | n.a. |
|  | Se-AbK | 6.51 ± 0.08 (5) | 12.4 ± 12.1 (5) | 2.51 ± 2.8 (5) | 6.5 |
|  | Se-AbK neg. | 6.51 ± 0.03 (3) | 5.79 ± 1.88 (3) | 8.71 ± 1.89 (3) | n.a. |

| Position replaced by TAG | ncAA | mean pH_50_  ± S.D. (n) | mean n_H_  ± S.D. (n) | mean Imax (nA) ± S.D. (n) | average  TE (%) |
| --- | --- | --- | --- | --- | --- |
| K470 | AzF | 6.33 ± 0.07 (5) | 6.16 ± 1.7 (5) | 2.57 ± 1.33 (6) | 6.1 |
|  | AzF neg. | 6.61 ± 0.08 (8) | 9.58 ± 10.46 (8) | 6.38 ± 2.83 (10) | n.a. |
|  | Bpa | 6.77 ± 0.05 (4) | 5.10 ± 1.68 (4) | 4 ± 1.71 (4) | 6.3 |
|  | Bpa neg. | 6.66 ± 0.04 (4) | 7.62 ± 2.08 (4) | 3.75 ± 0.86 (4) | n.a. |
|  | Se-AbK^#^ | 6.56 ± 0.05 (3) | 5.08 ± 2.05 (3) | 3.79 ± 1.75 (3) | 6.7 |
|  | Se-AbK neg. | 6.56 ± 0.05 (5) | 10.6 ± 8.66 (6) | 4.70 ± 1.69 (7) | n.a. |
| C471 | AzF | 6.60 ± 0.11 (10) | 12.4 ± 12.1 (10) | 2.84 ± 1.27 (10) | 8.4 |
|  | AzF neg. | 6.62 ± 0.06 (6) | 9.42 ± 2.33 (6) | 4.34 ± 0.88 (6) | n.a. |
|  | Bpa | 6.76 ± 0.04 (4) | 7.15 ± 3.55 (4) | 5.92 ± 1.60 (4) | 6.1 |
|  | Bpa neg. | 6.7 (1) | 2.35 (1) | 4.06 (1) | n.a. |
|  | Se-AbK^#^ | 6.57 ± 0.11 (5) | 8.08 ± 0.69 (5) | 3.84 ± 2.23 (5) | 7.9 |
|  | Se-AbK neg. | 6.48 ± 0.08 (3) | 13.4 ± 14.2 (4) | 4.54 (5) | n.a. |
| Q472 | AzF | 6.81 ± 0.11 (7) | 15.1 ± 11.2 (7) | 5.35 ± 1.68 (7) | 8.9 |
|  | AzF neg. | 6.71 ± 0.06 (6) | 6.64 ± 2.3 (6) | 7.33 ± 1.88 (6) | n.a. |
|  | Bpa | 6.69 ± 0.04 (4) | 6.25 ± 2.01 (4) | 5.58 ± 1.73 (4) | 8.0 |
|  | Bpa neg. | 6.70 ± 0.03 (4) | 6.38 ± 1.61 (4) | 2.70 ± 1.32 (4) | n.a. |
|  | Se-AbK^#^ | 6.49 ± 0.22 (3) | 6.59 (2) | 3.99 ± 0.16 (3) | 8.1 |
|  | Se-AbK neg.^#^ | 6.49 (2) | 15.4 (2) | 4.5 (2) | n.a. |
| K473 | AzF | 6.65 ± 0.05 (9) | 6.58 ± 2.14 (9) | 3.81 ± 1.34 (9) | 7.4 |
|  | AzF neg. | 6.68 (2) | 7.94 (2) | 4.99 (2) | n.a. |
|  | Bpa | 6.86 ± 0.04 (3) | 10.6 ± 8.35 (3) | 6 ± 0.97 (3) | 8.9 |
|  | Bpa neg. | 6.91 (2) | 13.1 (2) | 5.58 (2) | n.a. |
|  | Se-AbK^#^ | 6.58 ± 0.03 (6) | 17.6 ± 13.1 (6) | 4.55 ± 2.91 (6) | 14.0 |
|  | Se-AbK neg. | 6.58 ± 0.09 (4) | 7.24 ± 1.81 (4) | 6.66 ± 1.66 (4) | n.a. |
| E474 | AzF | 6.63 ± 0.07 (6) | 6.88 ± 3.5 (7) | 3.24 ± 1.02 (8) | 8.5 |
|  | AzF neg. | 6.69 ± 0.13 (8) | 7.1 ± 3.61 (8) | 4.38 ± 2.66 (8) | n.a. |
|  | Bpa | 6.69 ± 0.04 (4) | 5.94 ± 1.3 (4) | 7.62 ± 2.64 (4) | 9.5 |
|  | Bpa neg. | 6.51 ± 0.19 (4) | 11.4 ± 9.67 (4) | 3.86 ± 4.06 (4) | n.a. |
|  | Se-AbK | 6.60 ± 0.03 (3) | 5.05 ± 1.59 (3) | 2.59 ± 0.49 (3) | 9.5 |
|  | Se-AbK neg. | 6.58 ± 0.09 (3) | 6.23 ± 1.66 (3) | 3.35 ± 1.32 (3) | n.a. |
| A475 | AzF | 6.72 ± 0.18 (6) | 4.95 ± 1.94 (6) | 5.17 ± 2.08 (6) | 10.2 |
|  | AzF neg. | 6.64 ± 0.18 (6) | 8.66 ± 7.95 (6) | 5.97 ± 3.69 (6) | n.a. |
|  | Bpa | 6.67 ± 0.07 (4) | 7.41 ± 1.77 (4) | 8.25 ± 0.8 (4) | 13.7 |
|  | Bpa neg. | 6.66 ± 0.03 (3) | 6.82 ± 2.09 (3) | 5.79 ± 4.77 (3) | n.a. |
|  | Se-AbK | 6.58 ± 0.08 (8) | 5.14 ± 2.32 (8) | 4.70 ± 1.36 (8) | 7.6 |
|  | Se-AbK neg. | 6.62 (2) | 7.02 (2) | 2.75 (2) | n.a. |
| K476 | AzF | 6.64 ± 0.05 (7) | 8.22 ± 6.76 (7) | 2.32 ± 1.49 (8) | 12.7 |
|  | AzF neg. | 6.60 ± 0.20 (6) | 14.4 ± 13.4 (7) | 3.69 ± 2.24 (7) | n.a. |
|  | Bpa^#^ | 6.66 (1) | 9.13 (1) | 6.78 (1) | 10.3 |
|  | Bpa neg. | 6.59 ± 0.09 (4) | 9.38 ± 5.83 (4) | 4.41 ± 4.26 (4) | n.a. |
|  | Se-AbK | 6.59 ± 0.05 (9) | 6.83 ± 2.77 (9) | 3.37 ± 2.13 (9) | 9.6 |
|  | Se-AbK neg. | 6.46 ± 0.14 (5) | 8.82 ± 0.81 (5) | 3.07 ± 2.34 (5) | n.a. |

| Position replaced by TAG | ncAA | mean pH_50_  ± S.D. (n) | mean n_H_  ± S.D. (n) | mean Imax (nA) ± S.D. (n) | average  TE (%) |
| --- | --- | --- | --- | --- | --- |
| R477 | AzF | 6.54 (1) | 3.45 (1) | 3.11 (1) | 6.2 |
|  | AzF neg. | 6.69 ± 0.14 (4) | 5.98 ± 1.1 (4) | 3.71 ± 1.98 (4) | n.a. |
|  | Bpa^#^ | 6.64 (2) | 7.72 (2) | 6.14 (2) | 10.2 |
|  | Bpa neg. | 6.59 (2) | 7.86 (2) | 5.49 (2) | n.a. |
|  | Se-AbK | 6.60 ± 0.11 (7) | 5.32 ± 2.41 (7) | 3.05 ± 1.25 (7) | 9.3 |
|  | Se-AbK neg. | 6.59 ± 0.04 (3) | 6.36 ± 2.51 (3) | 5.29 ± 2.39 (3) | n.a. |
| S478 | AzF | 6.71 ± 0.04 (3) | 5.17 ± 3.15 (3) | 5.00 ± 2.89 (3) | 6.0 |
|  | AzF neg. ^#^ | 6.9 (1) | 13.5 (1) | 2.33 (2) | n.a. |
|  | Bpa | 6.74 ± 0.1 (4) | 11.9 ± 10.8 (4) | 6.11 ± 2.03 (4) | 6.9 |
|  | Bpa neg. | 6.65 (1) | 7.3 (1) | 7.38 (1) | n.a. |
|  | Se-AbK | 6.61 ± 0.08 (10) | 7.34 ± 1.39 (9) | 4.35 ± 2.37 (10) | 8.7 |
|  | Se-AbK neg. | 6.58 (2) | 5.22 (2) | 7.51 (2) | n.a. |
| S479 | AzF | 6.68 ± 0.11 (8) | 5.55 ± 1.70 (7) | 5.34 ± 3.30 (8) | 4.9 |
|  | AzF neg. ^#^ | 6.62 ± 0.04 (3) | 13.6 ± 9.99 (3) | 3.6 ± 2.41 (3) | n.a. |
|  | Bpa | 6.64 ± 0.06 (4) | 7.29 ± 2.05 (4) | 6.28 ± 2.42 (4) | 8.8 |
|  | Bpa neg. | 6.47 ± 0.18 (3) | 5.11 ± 2.55 (3) | 3.49 ± 2.47 (3) | n.a. |
|  | Se-AbK | 6.62 ± 0.07 (4) | 7.11 ± 1.99 (4) | 4.3 ± 2.73 (4) | 11.9 |
|  | Se-AbK neg. | 6.59 ± 0.06 (4) | 4.39 ± 0.83 (4) | 6.32 ± 3.3 (4) | n.a. |
| A480 | AzF | 6.67 (1) | 4.58 (1) | 10.4 (1) | 4.3 |
|  | AzF neg. ^#^ | 6.71 (1) | 5.73 (1) | 5.5 (2) | n.a. |
|  | Bpa | 6.67 ± 0.03 (4) | 6.01 ± 0.78 (4) | 7.80 ± 1.41 (4) | 13.1 |
|  | Bpa neg. | 6.5 (2) | 7.94 (2) | 6.48 (2) | n.a. |
|  | Se-AbK | 6.48 ± 0.1 (3) | 5.25 ± 4.47 (3) | 4.05 ± 3.03 (3) | 8.8 |
|  | Se-AbK neg. | 6.61 (1) | 18.8 (2) | 1.53 (2) | n.a. |
| D481 | AzF | 6.51 (1) | 5.52 (1) | 3.4 (1) | 4.1 |
|  | AzF neg. | 6.67 ± 0.04 (3) | 5.88 ± 0.42 (3) | 8.92 ± 2.66 (3) | n.a. |
|  | Bpa | 6.71 ± 0.05 (4) | 6.83 ± 0.86 (4) | 6.27 ± 1.38 (4) | 8.2 |
|  | Bpa neg. | 6.69 ± 0.12 (3) | 17.6 ± 15.4 (4) | 3.38 ± 3.92 (4) | n.a. |
|  | Se-AbK | 6.63 ± 0.04 (8) | 9.97 ± 9.09 (8) | 3.4 ± 2.04 (8) | 12.9 |
|  | Se-AbK neg. | 6.3 (1) | 6.06 (1) | 2.96 (1) | n.a. |
| K482 | AzF | 6.59 ± 0.27 (6) | 5.74 ± 2.9 (6) | 4.04 ± 1.11 (6) | 5.6 |
|  | AzF neg. | 6.64 ± 0.07 (8) | 7.65 ± 2.16 (8) | 4.24 ± 1.14 (8) | n.a. |
|  | Bpa | 6.67 (2) | 6.32 (2) | 6.6 ± 2.37 (3) | 7.7 |
|  | Bpa neg. | 6.62 (2) | 15.8 ± 15.6 (3) | 4.50 ± 3.55 (3) | n.a. |
|  | Se-AbK | 6.69 ± 0.07 (7) | 8.34 ± 7.09 (8) | 4.58 ± 2.04 (8) | 7.7 |
|  | Se-AbK neg. | 6.5 ± 0.08 (4) | 5.52 ± 0.87 (4) | 5.1 ± 1.86 (4) | n.a. |
| G483 | AzF^#^ | 6.66 ± 0.08 (11) | 8.24 ± 7.35 (11) | 3.73 ± 2.04 (12) | 5.3 |
|  | AzF neg. | 6.68 ± 0.11 (7) | 10.1 ± 8.32 (7) | 3.2 ± 1.34 (7) | n.a. |
|  | Bpa | 6.65 ± 0.09 (4) | 7.42 ± 2.44 (4) | 6.87 ± 3.35 (4) | 8.4 |
|  | Bpa neg. | 6.59 ± 0.04 (5) | 13.7 ± 12.2 (5) | 7.94 ± 1.69 (5) | n.a. |
|  | Se-AbK^#^ | 6.67 ± 0.1 (10) | 8.0 ± 2.33 (10) | 4.54 ± 2.43 (10) | 7.8 |
|  | Se-AbK neg. | 6.56 ± 0.06 (4) | 5.96 ± 1.34 (4) | 4.86 ± 2.09 (4) | n.a. |

| Position replaced by TAG | ncAA | mean pH_50_  ± S.D. (n) | mean n_H_  ± S.D. (n) | mean Imax (nA) ± S.D. (n) | average  TE (%) |
| --- | --- | --- | --- | --- | --- |
| V484 | AzF | n.a. | n.a. | 0 (0) | 8.1 |
|  | AzF neg. | n.a. | n.a. | 0 (0) | n.a. |
|  | Bpa^#^ | 6.56 ± 0.09 (4) | 7.39 ± 1.6 (4) | 2.47 ± 2.91 (4) | 9.8 |
|  | Bpa neg. | n.a. | n.a. | 0 (0) | n.a. |
|  | Se-AbK | n.a. | n.a. | 0 (0) | 10.8 |
|  | Se-AbK neg. | n.a. | n.a. | 0 (0) | n.a. |
| A485 | AzF^#^ | 6.74 ± 0.09 (6) | 9.34 ± 8.72 (6) | 4.04 ± 1.21 (7) | 6.1 |
|  | AzF neg. | 6.73 ± 0.13 (8) | 14.5 ± 11.5 (8) | 3.25 ± 1.4 (9) | n.a. |
|  | Bpa | 6.51 ± 0.12 (5) | 14.7 ± 11.0 (5) | 4.75 ± 2.9 (5) | 14.7 |
|  | Bpa neg. | 6.7 ± 0.06 (4) | 6.55 ± 1.32 (4) | 3.2 ± 1.97 (4) | n.a. |
|  | Se-AbK | 6.6 ± 0.14 (5) | 11.9 ± 8.85 (5) | 5.17 ± 2.44 (5) | 11.1 |
|  | Se-AbK neg. | 6.53 ± 0.06 (4) | 6.69 ± 3.22 (4) | 2.84 ± 2.82 (4) | n.a. |
| L486 | AzF | 6.8 ± 0.08 (8) | 7.68 ± 5.14 (8) | 6.23 ± 2.71 (9) | 6.8 |
|  | AzF neg. | 6.73 ± 0.07 (7) | 7.42 ± 0.92 (7) | 5.95 ± 2.58 (7) | n.a. |
|  | Bpa^#^ | 6.73 ± 0.11 (7) | 14.2 ± 9.56 (7) | 3.69 ± 2 (7) | 14.4 |
|  | Bpa neg. | 6.6 (2) | 6.31 (2) | 6.35 (2) | n.a. |
|  | Se-AbK^#^ | 6.59 (2) | 7.05 (2) | 4.01 (2) | 10.2 |
|  | Se-AbK neg. | 6.6 ± 0.06 (4) | 5.14 ± 1.04 (4) | 2.9 ± 2.01 (5) | n.a. |
| S487 | AzF^#^ | n.a. | n.a. | 1.01 ± 1.13 (5) | 9.3 |
|  | AzF neg. | n.a. | n.a. | 0 (0) | n.a. |
|  | Bpa^#^ | n.a. | n.a. | 2.28 ± 1.98 (6) | 18.1 |
|  | Bpa neg. ^#^ | 6.49 (2) | 11.6 (2) | 0.49 (2) | n.a. |
|  | Se-AbK | n.a. | n.a. | 0 (0) | 9.4 |
|  | Se-AbK neg. | n.a. | n.a. | 0 (0) | n.a. |
| L488 | AzF^#^ | 6.78 (1) | 6.25 (1) | 4.91 (1) | 6.0 |
|  | AzF neg. ^#^ | 6.69 (1) | 7.85 (1) | 4.51 (2) | n.a. |
|  | Bpa | 6.66 ± 0.19 (3) | 12.0 ± 12.1 (3) | 4.14 ± 2.14 (3) | 10.7 |
|  | Bpa neg. | 6.6 (1) | 5.31 (1) | 6.84 (1) | n.a. |
|  | Se-AbK^#^ | 6.65 ± 0.02 (3) | 13.4 ± 10.3 (3) | 5.56 ± 3.17 (3) | 8.0 |
|  | Se-AbK neg. | 6.67 (1) | 11.6 (1) | 5.9 (1) | n.a. |
