# Supplementary material for: High-throughput characterization of photocrosslinker-bearing ion channel variants to map residues critical for function and pharmacology
Source: PLoS Biol. 2021 Sep 7;19(9):e3001321. doi: 10.1371/journal.pbio.3001321 (PMC8448361; doi:10.1371/journal.pbio.3001321)
Supplement: S2 Table — Values are depicted as mean ± SD, and number in brackets indicates number of cells. The underlying data have been deposited at zenodo.org (https://doi.org/10.5281/zenodo.4906985; file 32). APC, automated patch clamp; SD, standard deviation. (DOCX) [file pbio.3001321.s017.docx]

| Clone | Current (nA) at 30 µM agonist | Current (nA) at saturating agonist |
| --- | --- | --- |
| P2X2 WT | 8.14 ± 2.87 (26) | 6.01 ± 2.86 (26) |
| P2X2 K296AzF | 3.79 ± 3.44 (40) | 3.07 ± 2.60 (40) |
| P2X2 K296X w/o AzF | 0.78 ± 0.56 (2) | 1.28 ± 0.69 (2) |
| P2X2 K296Bpa | 3.27 ± 3.04 (51) | 2.25 ± 2.31 (51) |
| P2X2 K296X w/o Bpa | 0.06 ± 0.03 (2) | 0.34 ± 0.21 (2) |
| GluA2 WT | 1.04 ± 1.31 (46) | 5.69 ± 3.76 (47) |
| GluA2 Y533AzF | 0.09 ± 0.22 (27) | 1.21 ± 0.96 (30) |
| GluA2 Y533X w/o AzF | 0.01 ± 0.01 (2) | 0.23 ± 0.15 (2) |
| GluA2 S729AzF | 0.03 ± 0.08 (15) | 0.39 ± 0.33 (17) |
| GluA2 S729X w/o AzF | No current | No current |
| GluA2 S729Bpa | 0.07 ± 0.13 (13) | 0.28 ± 0.24 (16) |
| GluA2 S729X w/o Bpa | No current | No current |
