# Supplementary material for: High-throughput characterization of photocrosslinker-bearing ion channel variants to map residues critical for function and pharmacology
Source: PLoS Biol. 2021 Sep 7;19(9):e3001321. doi: 10.1371/journal.pbio.3001321 (PMC8448361; doi:10.1371/journal.pbio.3001321)
Supplement: S3 Table — Displayed are mean and SD of t½; (n) equals number of cells. (*) denotes significant difference between t½ of current decay compared to WT, p < 0.05; (***): p < 0.001; Mann–Whitney test. The underlying data have been deposited at zenodo.org (https://doi.org/10.5281/zenodo.4906985; file 10). hASIC1a, human acid-sensing ion channel 1a; ncAA, noncanonical amino acid; SD, standard deviation; WT, wild type. (DOCX) [file pbio.3001321.s018.docx]

| Clone | t½ ± S.D. (ms) | P value | n |
| --- | --- | --- | --- |
| hASIC1a WT | 818 ± 750 | - | 9 |
| T239Bpa | 224 ± 176* | 0.036 | 6 |
| D357AzF | 93.8 ± 40.9*** | 0.0002 | 10 |
