# Supplementary material for: High-throughput characterization of photocrosslinker-bearing ion channel variants to map residues critical for function and pharmacology
Source: PLoS Biol. 2021 Sep 7;19(9):e3001321. doi: 10.1371/journal.pbio.3001321 (PMC8448361; doi:10.1371/journal.pbio.3001321)
Supplement: S4 Table — Displayed are mean and SD of half-maximal inactivation (pH50 SSD) and Hill slope (nH) as well as number of experiments (n). (*) denotes significant difference from WT, p < 0.05, (**): p < 0.005, (***): p < 0.001, (****): p < 0.0001, Mann–Whitney test. The underlying data have been deposited at zenodo.org (https://doi.org/10.5281/zenodo.4906985; files 34 and 35). APC, automated patch clamp; hASIC1a, human acid-sensing ion channel 1a; SD, standard deviation; SSD, steady-state desensitization; WT, wild type. (DOCX) [file pbio.3001321.s019.docx]

| Clone | pH_50_ SSD ± S.D. | P value | n_H_ SSD ± S.D. | P value | n |
| --- | --- | --- | --- | --- | --- |
| WT | 6.91 ± 0.02 | - | 3.16 ± 0.42 | - | 40 |
| E177Bpa | 7.15 ± 0.01**** | <0.0001 | 7.95 ± 3.3** | 0.0035 | 12 |
| T236AzF | 6.85 ± 0.02 | 0.5541 | 5.68 ± 1.35 | 0.1134 | 4 |
| T236Bpa | 6.85 ± 0.02 | 0.3506 | 7.37 ± 1.67** | 0.0092 | 16 |
| T239Bpa | 6.79 ± 0.07 | 0.3447 | 2.02 ± 0.61 | 0.1587 | 12 |
| K343Bpa | 6.97 ± 0.04 | 0.2135 | 2.31 ± 0.59 | 0.3279 | 14 |
| E344AzF | 7.0 ± 0.04 | 0.1558 | 4.87 ± 1.9 | 0.4739 | 5 |
| E344Bpa | 7.07 ± 0.02*** | 0.0004 | 5.31 ± 0.76* | 0.0876 | 12 |
| D351Bpa | 6.98 ± 0.03 | 0.7032 | 3.73 ± 0.87 | 0.7474 | 7 |
| E355AzF | 7.07 ± 0.03* | 0.0459 | 2.56 ± 0.6 | 0.1640 | 8 |
| E355Bpa | 7.11 ± 0.01**** | <0.0001 | 5.0 ± 0.52 | 0.4647 | 7 |
| K356AzF | 6.76 ± 0.06 | 0.2881 | 4.32 ± 2.5 | 0.3639 | 7 |
| K356Bpa | 6.81 ± 0.02 | 0.1406 | 9.07 ± 4.45 | 0.4252 | 12 |
| D357AzF | 6.70 ± 0.50 | 0.2394 | 16.2 ± 11.9 | 0.7819 | 6 |
| D357Bpa | 6.84 ± 0.03 | 0.3937 | 12.9 ± 4.8**** | <0.0001 | 13 |
|  |  |  |  |  |  |
| F69Bpa | 7.01 ± 0.06* | 0.0175 | 13.3 ± 13.9*** | 0.0002 | 8 |
| Y71AzF | 7.05 ± 0.02* | 0.0175 | 17.1 ± 2.93** | 0.0018 | 3 |
| W287AzF | 7.08 ± 0.01*** | 0.0010 | 10.9 ± 1.5** | 0.0019 | 6 |
