# Supplementary material for: High-throughput characterization of photocrosslinker-bearing ion channel variants to map residues critical for function and pharmacology
Source: PLoS Biol. 2021 Sep 7;19(9):e3001321. doi: 10.1371/journal.pbio.3001321 (PMC8448361; doi:10.1371/journal.pbio.3001321)
Supplement: S5 Table — Cells were incubated at SSD-inducing pH for 2 minutes with or without 3 μM BigDyn before activation at pH 5.6, and the currents were normalized to the average of 2 preceding control pulses after conditioning at pH 7.6. Values are indicated as mean ± SD; (n) equals number of cells. (*) denotes significant difference between currents with and without 3 μM BigDyn, p < 0.05; (**): p < 0.01; (***): p < 0.001; ns: not significant; Mann–Whitney test. The underlying data have been deposited at zenodo.org (https://doi.org/10.5281/zenodo.4906985; file 11). AzF, 4-Azido-l-phenylalanine; hASIC1a, human acid-sensing ion channel 1a; SD, standard deviation; SSD, steady-state desensitization; WT, wild type. (DOCX) [file pbio.3001321.s020.docx]

| Clone | pH | 3 µM BigDyn | Current after pH_SSD_ 1 (%) | Current after pH_SSD_ 2  (±BigDyn, %) | P value | Current after pH_SSD_ 3  (±BigDyn, %) | Current after pH_SSD_ 4 (%) | P value | n |
| --- | --- | --- | --- | --- | --- | --- | --- | --- | --- |
| WT | 6.6 | No | 33.6 ± 25.3 | 15.4 ± 17.1* | 0.0106 | 16.0 ± 17.0 | 14.9 ± 17.2^ns^ | 0.7114 | 24 |
| WT | 6.6 | Yes | 27.0 ± 23.2 | 29.7 ± 25.0^ns^ | 0.9118 | 38.0 ± 34.8 | 20.7 ± 39.3^ns^ | 0.0939 | 10 |
| T236AzF | 6.6 | No | 16.3 ± 18.1 | 7.65 ± 8.63^ns^ | 0.07 | 15.1 ± 19.5 | 7.79 ± 8.33^ns^ | 0.1479 | 19 |
| T236AzF | 6.6 | Yes | 13.7 ± 8.85 | 34.0 ± 20.0** | 0.0032 | 46.0 ± 31.2 | 17.3 ± 15.5^**^ | 0.0039 | 11 |
| K343AzF | 6.7 | No | 18.2 ± 27.3 | 8.75 ± 12.9^ns^ | 0.2480 | 15.9 ± 41.7 | 4.49 ± 6.36^ns^ | 0.1738 | 22 |
| K343AzF | 6.7 | Yes | 54.1 ± 96.5 | 10.5 ± 27.4^***^ | 0.0006 | 42.2 ± 75.9 | 7.61 ± 12.2^**^ | 0.0013 | 18 |
| E344AzF | 6.6 | No | 9.65 ± 12.0 | 2.23 ± 6.39^*^ | 0.0253 | 1.50 ± 11.9 | 0.78 ± 11.1^ns^ | 0.3888 | 19 |
| E344AzF | 6.6 | Yes | 8.29 ± 8.69 | 4.21 ± 5.56^*^ | 0.0326 | 18.0 ± 19.0 | 5.18 ± 7.36^**^ | 0.0077 | 19 |
| D351AzF | 6.8 | No | 23.8 ± 23.7 | 14.1 ± 16.9^ns^ | 0.1371 | 30.5 ± 27.2 | 23.5 ± 30.4^ns^ | 0.2982 | 12 |
| D351AzF | 6.8 | Yes | 14.9 ± 18.2 | 64.1 ± 23.4** | 0.0041 | 80.9 ± 47.0 | 26.8 ± 15.8^***^ | 0.0006 | 7 |
| E355AzF | 7.0 | No | 9.85 ± 5.36 | 9.38 ± 5.73^ns^ | 0.7366 | 19.4 ± 14.1 | 8.87 ± 6.69^ns^ | 0.0516 | 8 |
| E355AzF | 7.0 | Yes | 17.9 ± 11.0 | 67.1 ± 52.2*** | 0.0008 | 125 ± 90.7 | 80.4 ± 99.3* | 0.0241 | 14 |
| K356AzF | 6.6 | No | 9.70 ± 13.5 | 7.94 ± 11.1^ns^ | 0.1465 | 14.8 ± 18.4 | 2.20 ± 1.57*** | 0.0003 | 14 |
| K356AzF | 6.6 | Yes | 23.4 ± 31.5 | 43.2 ± 33.8^ns^ | 0.1145 | 70.2 ± 46.8 | 10.6 ± 12.1*** | 0.0003 | 11 |
