# Supplementary material for: High-throughput characterization of photocrosslinker-bearing ion channel variants to map residues critical for function and pharmacology
Source: PLoS Biol. 2021 Sep 7;19(9):e3001321. doi: 10.1371/journal.pbio.3001321 (PMC8448361; doi:10.1371/journal.pbio.3001321)
Supplement: S6 Table — Cells were incubated with 100 nM PcTx1 for 2 minutes before activation at pH 5.6, and the current was normalized to the average of the 4 preceding and following control pulses after conditioning at pH 7.4. Values are indicated as mean ± SD; (n) equals number of cells. (*) denotes significant difference between currents at different pH, p < 0.05; (**): p < 0.01; ns: not significant; 1-way ANOVA with Tukey multiple comparisons test (AzF variants) or Mann–Whitney test (Bpa variants). The underlying data have been deposited at zenodo.org (https://doi.org/10.5281/zenodo.4906985; file 12). AzF, 4-Azido-l-phenylalanine; Bpa, 4-Benzoyl-l-phenylalanine; hASIC1a, human acid-sensing ion channel 1a; PcTx1, psalmotoxin 1; SD, standard deviation; WT, wild type. (DOCX) [file pbio.3001321.s021.docx]

|  | pH 1 (7.4) | | | pH 2 (7.3) | | | pH 3 (7.0;  7.2 for WT) | | | | P value | | | | |
| --- | --- | --- | --- | --- | --- | --- | --- | --- | --- | --- | --- | --- | --- | --- | --- |
| Clone | Normalized response (%) ± S.D. | | n | Normalized response (%) ± S.D. | | n | Normalized response (%) ± S.D. | | | n | pH 1 vs  pH 2 | pH 2 vs  pH 3 | | | pH 1 vs  pH 3 |
| WT | 38.2 ± 31.7 | | 8 | 23.7 ± 15.4 | | 11 | 2.06 ± 2.50 | | | 7 | 0.2907^ns^ | 0.0907^ns^ | | | 0.0060** |
| T236AzF | 188 ± 82.1 | | 5 | 64.0 ± 31.5 | | 4 | 46.5 ± 46.4 | | | 8 | 0.0147* | 0.8708^ns^ | | | 0.0017** |
| E344AzF | 58.0 ± 37.4 | | 3 | 23.9 ± 17.2 | | 11 | 4.14 ± 4.41 | | | 6 | 0.0303* | 0.1191^ns^ | | | 0.0020** |
| E355AzF | 66.9 ± 55.5 | | 13 | 48.8 ± 63.4 | | 8 | 5.03 ± 5.60 | | | 8 | 0.7051^ns^ | 0.2071^ns^ | | | 0.0281* |
| K356AzF | 92.2 ± 40.1 | | 12 | 51.7 ± 33.2 | | 6 | 43.6 ± 51.6 | | | 11 | 0.1737^ns^ | 0.9298^ns^ | | | 0.0343* |
| D357AzF | 902 ± 926 | | 17 | 331 ± 441 | | 6 | 260 ± 383 | | | 9 | 0.6407^ns^ | >0.9999^ns^ | | | 0.3072^ns^ |
|  | | | | | | | | | | | | | | | |
| D357AzF | pH 4 (6.8) | | | pH 5 (6.7) | | | pH 6 (6.6) | | | | pH 7 (6.5) | | | | |
|  | 1.80 ± 1.78 | | 6 | 0.72 ± 1.68 | | 5 | 3.58 ± 6.22 | | | 7 | 2.20 ± 2.16 | | | 7 | |
| P values D357AzF | | | | | | | | | | | | | | | |
| pH 1 vs pH 4 | | pH 1 vs pH 5 | | | pH 1 vs pH 6 | | | pH 1 vs pH 7 | pH 2 vs pH 4 | | | | pH 2 vs pH 5 | | |
| 0.0187* | | 0.0186* | | | 0.0191* | | | 0.0188* | 0.7327^ns^ | | | | 0.7298^ns^ | | |
| pH 2 vs pH 6 | | pH 2 vs pH 7 | | | pH 3 vs pH 4 | | | pH 3 vs pH 5 | pH 3 vs pH 6 | | | | pH 3 vs pH 7 | | |
| 0.7376^ns^ | | 0.7338^ns^ | | | 0.6245^ns^ | | | 0.6196^ns^ | 0.6326^ns^ | | | | 0.6263^ns^ | | |
| pH 4 vs pH 5 | | pH 4 vs pH 6 | | | pH 4 vs pH 7 | | | pH 5 vs pH 6 | pH 5 vs pH 7 | | | | pH 6 vs pH 7 | | |
| 0.9943^ns^ | | 0.9998^ns^ | | | >0.9999^ns^ | | | 0.9797^ns^ | 0.9567^ns^ | | | | >0.9999^ns^ | | |
| Bpa variants | | | | | | | | | | | | | | | |
| Clone | pH 1 (7.4) | | | pH 2 (7.0) | | | P value | | | | | | | | |
|  | Normalized response (%) ± S.D. | | n | Normalized response (%) ± S.D. | | n | pH 1 vs pH 2 | | | | | | | | |
| WT | 40.9 ± 5.25 | | 6 | 11.4 ± 9.15 | | 6 | 0.0022** | | | | | | | | |
| T236Bpa | 62.1 ± 47.5 | | 6 | 23.0 ± 24.8 | | 7 | 0.3660^ns^ | | | | | | | | |
| E344Bpa | 114 ± 25.6 | | 6 | 4.00 ± 2.64 | | 5 | 0.0043** | | | | | | | | |
| E355Bpa | 102 ± 41.4 | | 5 | 7.89 ± 3.72 | | 6 | 0.0043** | | | | | | | | |
| K356Bpa | 95.4 ± 8.24 | | 6 | 15.6 ± 22.1 | | 6 | 0.0022** | | | | | | | | |
| D357Bpa | 187 ± 33.4 | | 6 | 6.01 ± 4.92 | | 6 | 0.0022** | | | | | | | | |
