# Supplementary material for: High-throughput characterization of photocrosslinker-bearing ion channel variants to map residues critical for function and pharmacology
Source: PLoS Biol. 2021 Sep 7;19(9):e3001321. doi: 10.1371/journal.pbio.3001321 (PMC8448361; doi:10.1371/journal.pbio.3001321)
Supplement: S1 Text — (DOCX) [file pbio.3001321.s022.docx]

### Supplementary methods

Synthesis of ncAA-MEs. AzF (4-Azido-l-phenylalanine) and Bpa (4-Benzoyl-l-phenylalanine) were purchased from Chem Impex (IL, USA) and Bachem Bio (Switzerland), respectively. For the synthesis of ncAA-methylesters [1], TMSCl (chlorotrimethylsilane, 2 equivalents) was added to the amino acid in a round-bottom flask. Anhydrous methanol was added to dissolve the starting material and the reaction mixture was stirred at room temperature over night under nitrogen atmosphere. TMSCl (1 equiv.) was added every 24h until complete conversion of starting material (reaction monitored by LC-MS (liquid-chromatography - mass spectrometry)). The solvents were evaporated yielding an off-white solid, which was purified by preparative HPLC (high-performance liquid chromatography) when necessary. ncAA-methylesters were dissolved in DMSO (dimethyl sulfoxide) and stock solutions were stored at ‑20 ˚C.

**Mass spectrometry.** For filter-assisted in-solution digestion, eluted protein samples (25 µl) were incubated with 250 µl UA solution (8 M Urea in 0.1 M Tris/HCl (pH 8.5), 10 mM TCEP) for 10 min and passed through a 0.5 ml Amicon Ultra centrifugal filter (30 kD cutoff, Sigma Aldrich, Germany) by centrifugation at 14000 g for 10 min. Samples were washed with 200 µl UA by centrifugation and incubated with 100 µl iodoacetamide solution (50 mM iodoacetamide in UB) at RT for 20 min in the dark. After centrifugation, samples were washed twice with 200 µl UB (8 M Urea in 0.1 M Tris/HCl (pH 8.5)) and twice with 200 µl of 50 mM ammonium bicarbonate before incubation with 40 µl of chymotrypsin (0.5 µg) in 25 mM ammonium bicarbonate at 37 °C over night. The next morning, 20 µl trypsin (0.25 µg) in 25 mM ammonium bicarbonate were added and the samples were incubated at 37 °C for 4 hours. Using a new collection tube, samples were centrifuged and the columns washed with 50 µl 0.5 M NaCl. After addition of 10 µl 10 % (v/v) trifluoroacetic acid, peptide solutions were concentrated to 40 µl on a Savant SPD1010 SpeedVac Concentrator (Thermo Fisher Scientific, Germany) and loaded onto the LC-MS system.

Peptide solutions were analyzed by liquid chromatography followed by tandem mass spectrometry (LC-MS/MS) on Ultimate 3000 RSLC nano-HPLC systems coupled to an Orbitrap Fusion mass spectrometer (all from Thermo Fisher Scientific)[2]. The mass spectrometer was equipped with a nano-ESI source (Nanospray Flex or EASY-Spray™ ion source, Thermo Fisher Scientific) and external column heater (Phoenix S&T, PA, USA) to enable the use of self-packed emitter columns (PicoFrit, New Objective, MA, USA). Samples were loaded onto an RP (reverse phase) C18 pre-column (Acclaim PepMap, 300 μm × 5 mm, 5 μm, 100 Å, Thermo Fisher Scientific) at a flow rate of 30 μl/min and washed with 0.1 % (v/v) TFA for 15 min at 30 μL/min before elution and separation on a self-packed RP C18 separation column (PicoFrit, 75 μM × 250–500 mm, 15 µm tip diameter, packed with *ReproSil-Pur* C18*-*AQ, 1.9 μm, 120 Å, Dr. Maisch, Germany) equilibrated with 3% solvent B (solvent A: 0.1 % (v/v) FA (formic acid), solvent B: ACN, 0.08 % (v/v) FA). A gradient from 3–40 % solvent B within 90 min at 300 nl/min was used to elute peptides from the separation column. A voltage of 1.9 kV was applied between the column and the mass spectrometer entrance for positive ionization. Data were acquired in data-dependent MS/MS mode using HCD (high energy collisional dissociation, stepped normalized collision energies (NCE): 28 %) for fragmentation. For data acquisition, each high-resolution full scan (*m/z* 300 to 1500, R = 120000) in the Orbitrap was followed by high-resolution product ion scans (R = 15000, minimum charge states 2+ to 6+) within 5 seconds, starting with the most intense signal in the full scan mass spectrum (isolation window 2 Th); the target value and maximum accumulation time were 50000 and 200 ms. Dynamic exclusion (duration 60 s, window ± 2 ppm) was enabled. The Xcalibur software (version 4.1, Thermo Fisher Scientific) was used for data acquisition and raw data inspection.

Raw data generated by LC-MS/MS was processed using the Thermo Proteome Discoverer (version 2.0.0.802, Thermo Fisher Scientific) by matching MS and MS/MS data to the human reference proteome downloaded from the UniProt database (http://www.uniprot.org, FASTA file) or an *in-house* database containing the amino acid sequence of hASIC1a as well as common contaminants. For identification of proteins and evaluation of the sequence coverage, the Sequest search engine was used (maximum mass differences of 10 ppm and 0.02 Da for MS and MS/MS data). Further parameters were unspecific enzymatic cleavage, alkylation of cysteines as a static modification and oxidation of methionines as well as replacement of alanine by Bpa as variable modifications. Mass spectra were exported from Thermo Proteome Discoverer and further annotated in Illustrator CC 2019.

### Supplementary references

1. Li J, Sha Y. A convenient synthesis of amino acid methyl esters. Molecules. 2008;13(5):1111-9. PubMed PMID: 18560331; PubMed Central PMCID: PMCPMC6245331.

2. Haupl B, Ihling CH, Sinz A. Protein Interaction Network of Human Protein Kinase D2 Revealed by Chemical Cross-Linking/Mass Spectrometry. J Proteome Res. 2016;15(10):3686-99. doi: 10.1021/acs.jproteome.6b00513. PubMed PMID: 27559607.

3. Yamada KA, Tang CM. Benzothiadiazides inhibit rapid glutamate receptor desensitization and enhance glutamatergic synaptic currents. J Neurosci. 1993;13(9):3904-15. PubMed PMID: 8103555; PubMed Central PMCID: PMCPMC6576449.

4. Ahmed R, Spikings E, Zhou S, Thompsett A, Zhang T. Pre-hybridisation: an efficient way of suppressing endogenous biotin-binding activity inherent to biotin-streptavidin detection system. J Immunol Methods. 2014;406:143-7. doi: 10.1016/j.jim.2014.03.010. PubMed PMID: 24657589.

5. Praul CA, Brubaker KD, Leach RM, Gay CV. Detection of endogenous biotin-containing proteins in bone and cartilage cells with streptavidin systems. Biochem Biophys Res Commun. 1998;247(2):312-4. doi: 10.1006/bbrc.1998.8757. PubMed PMID: 9642122.

6. Borg CB, Braun N, Heusser SA, Bay Y, Weis D, Galleano I, et al. Mechanism and site of action of big dynorphin on ASIC1a. Proc Natl Acad Sci U S A. 2020. doi: 10.1073/pnas.1919323117. PubMed PMID: 32165542.
