# Supplementary material for: High-throughput characterization of photocrosslinker-bearing ion channel variants to map residues critical for function and pharmacology
Source: PLoS Biol. 2021 Sep 7;19(9):e3001321. doi: 10.1371/journal.pbio.3001321 (PMC8448361; doi:10.1371/journal.pbio.3001321)
Supplement: Attachment — Submitted filename: Response to reviewers.pdf [file pbio.3001321.s023.pdf]

(1) Reviewer 1 and 2's comments should be thoroughly addressed, with additional experiments where requested.

We thank the reviewers for their positive feedback and constructive criticism. We have now addressed all comments and conducted a number of additional experiments. Specifically, we have collected more data on the SSD curves and provide new results on peptide modulation. We also demonstrate that our approach can be used for different types of ion channels. Lastly, we have assembled further FACS sorting controls to address the concerns raised by the reviewers. Note that we have made changes to all figures in the manuscript and have added new supplementary figures and tables where necessary.

(2) Reviewer 3 makes two major comments, one about the effects of Pctx1 on the D357AzF variant, in terms of proton sensitivity and channel desensitization, and another one about testing more substitutions at position D357. We think point 1 (the effects of Pctx1 on the D357AzF variant) is a valuable suggestion and should be addressed. Regarding the more open-ended recommendation ("it would be more informative if the authors could perform more detailed studies on the D357 position using photocrosslinkers that would introduce amino acid residues with very distinct properties."), we do not think addressing this point experimentally is necessary for publication in our journal, assuming you will validate your method with at least another ion channel, as we requested before. However, we invite you to consider the point, as this could potentially address reviewer 4's concern about lack of enough significant biological insights (see below). But again, this will be up to you.

We would like to thank the editor for clarifying the expectations with regards to these concerns. To address the first point of the reviewer #3, we now include a significantly expanded data set on D357AzF, as well as new experimental results on the corresponding D357Bpa variant. The results and implications are addressed in detail below, as well as in the revised manuscript. We hope this additional work also serves to alleviate the concerns of reviewer #4 regarding the need for added biological insight.

(3) Reviewer 4 is critical of the work because of lack of sufficient novel biological insights and lack of transferability of the method, due to its cost. We should note that offering novel biological insights is not a condition for publication for our Methods papers. However, as mentioned above, addressing reviewer 3's comments regarding the substitutions at position D357 could potentially address this reviewer's concerns about lack of biological insights. Therefore, we invite you to consider this point, and if you wish to add more data, we will welcome them, but will not press for additional experiments here. Regarding the concern about cost of the equipment, we think you should add a paragraph in the Discussion to discuss transferability of the technology.

Again, we are grateful for the clarifications regarding the expectations and the requirements for publication. We now provide new data on additional substitutions and pH conditions tested at position D357 and have added a paragraph in the discussion to elaborate on the accessibility of the technology in academia in response to the comment by reviewer #4.

(4) I would also like to remind you that we expect you to validate the method with at least another ion channel.

We have now validated our APC screening approach on selected variants of the glutamate-gated rat GluA2 receptors and ATP-gated rat P2X2 receptors to show that the method can successfully be applied to other ion channels (and is similarly efficient when compared with previous work on corresponding ncAA-containing channels using manual patch-clamp). This is shown in the new Figure S7 and Table S2 and described at the end of the first results section.

(5) Finally, it is important that you update your competing interest statement to make it clear that one of the co-authors work for the APC manufacturer.
We apologise for this oversight and have now updated the competing interest statement accordingly.

REVIEWS:

Reviewer #1: The manuscript by Braun and colleagues report an extensive investigation of site-specific incorporation of non-canonical amino acids (ncaa) into the acid-sensing ion channel ASIC1a. A key point of emphasis of the paper is the development of a high throughput strategy using automated patch clamp recording. The data inform about regions of the channel protein that are involved in pH sensing and interaction with peptide ligands. The scale and scope of the study are unprecedented, and the data quality is high. I particularly like the use of 'snake plots' to summarize results. There are a few methodological and interpretational concerns that should be addressable.

Major concerns

1. Transfection efficiency (TE) is very low for the mutants compared with WT-ASIC, but the experimental conditions for assessing this are different. According to the Methods, TE for WT-ASIC was assessed using WT-GFP, whereas TE for mutants was assessed using GFP with a nonsense mutation. Therefore, the TE value reported for mutants is a proxy for at least two phenomena (transfection efficiency and efficiency of nonsense suppression). There is value in evaluating a subset of mutants with WT-GFP (or evaluate TE for WT-ASIC with GFP-TAG) to help dissect the relative weighting of these two factors.
We thank the reviewer for this suggestion and refer to the new Figure S1, where we compare TE and number of wells with current for hASIC1a WT transfected with GFP WT, Y151AzF and Y40Bpa. While TE is roughly halved when using a TAG-containing GFP (61.6% for GFP WT, 29.9% for GFP Y151AzF and 33.1% for GFP Y40Bpa, Figure S1A), it is still considerably higher than the mean of 11% we found for hASIC1a variants (Figure S1B), indicating that both TE and non-sense suppression contribute to the TE value reported via FACS. However, given the overall similar success rates when considering the number of wells showing proton-gated currents (48% for GFP WT vs. 39% or 42% for GFP Y151AzF and Y40Bpa), we recognize using a TAG-containing GFP for hASIC1a WT would have provided a better control.

2. Also related to transfection, which involved 4 separate plasmids, some additional controls may prove valuable. The main concern is that GFP fluorescence will not correlate 100% with expression of the channel. This makes it difficult to interpret the meaning of absent current in cell transfected with mutants, and therefore no conclusions can be reliable drawn based on absent

current. As the authors discussed in the limitations section (page 22) a better experimental strategy would have placed both ASIC and GFP (or GFP-TAG) in the same plasmid vector.
We thank the reviewer for this comment and agree that, in retrospect, putting ASIC1a and GFP into the same vector would have been a better strategy. To make this more clear, we now also refer to a study by the MacLean lab that uses a C-terminal GFP tag to follow TAG suppression (see also below). We hope this will help guide future experimental designs.

3. There is a great risk for sampling bias in experiments on non-sorted cells. On page 6, the authors defined specific vs unspecific incorporation based on current amplitude determined in the presence vs absence of ncaa, but the recordings on cells grown in the absence of ncaa were not done on FACS sorted cells. Although the authors addressed this on page 22, I don't think this is reliable because of the very low average TE for mutants. The authors need to consider, and probably report, the total number of recorded cells and the % of recorded cells with current vs no current for experiments investigating non-sorted populations, then describe criteria for how these data were interpreted.
We have re-analyzed a data subset from hASIC1a variants that were assessed for ncAA incorporation in the N-terminus, ECD and C-terminus in order to compare the number of recorded cells and cells with current in presence and absence of ncAA (please see new Figure S1C). The data shows that while the percentage of cells with current is generally lower for cells grown in the absence of ncAA, we do observe currents for those positions where incorporation tends to be unspecific, e.g. throughout the C-terminus and in some positions in the N-terminus. For the control samples an average of 9.8 out of 16 possible wells had a cell with >100 MΩ seal, and we observed currents in 1.6 wells on average. We therefore conclude that despite some shortcomings, the employed strategy using non-sorted controls allows us to detect at least those positions with >15% unspecific incorporation. This is now explicitly outlined in the discussion.

4. The criteria for excluding cells need further discussion. On page 5, a typical experiment is described in which 12 conditions across a 384-well plate are interrogated. This translates to 32 replicates per variant/condition. However, the number of replicates reported in Table S1 is 3-10 with most being less than 6. Additional information is needed to clarify the number of attempted replicates vs number of final data, and the criteria used to exclude recordings.
Point well taken. We agree this could have been made more clear. We have adjusted the sentence on page 5 to better reflect that the number of attempted replicates per condition is 16:
"The 384-well system of the SyncroPatch 384PE allows for parallel concentration response curve measurements on 24 different samples, enabling us to test 11 different channel variants with corresponding incorporation controls (cells grown in the absence of ncAA), as well as hASIC1a WT and untransfected cells in less than one hour, with up to 16 replicates per sample."
As for the well exclusion criteria, please refer to the paragraph on data analysis in the method section.

Minor concerns

a. A pH titration curve should be included in Fig. 2
We have added a pH response curve for hASIC1a WT and T236AzF in Figure 2A.

b. The first section of Results should have at least one paragraph break to improve readability.
We thank the reviewer for this comment and have added paragraph breaks to improve readability.

c. Throughout the text and tables, too many significant digits are presented.
We have adjusted the number of significant digits throughout the manuscript.

d. Page 24, 3rd line from bottom, the abbreviation 'mio.' may be a typo or a non-standard abbreviation.
We have changed the wording to 'million'.

Reviewer #2, David MacLean: The work by Braun et al. represents a promising direction for the field of non-canonical amino acid incorporation in ion channels, and channel biophysics in general. By combining large scale mutagenesis with FACS and automated patch clamp (APC), the authors screen just over 100 positions in human ASIC1a for incorporation of three distinct ncAAs. The approach is of interest to the many who use (or wish to use) ncAA incorporation in membrane proteins in general, and ion channels more specifically. Further, their data set on hASIC1a sites which support incorporation is gives a generous 'head start' to labs wishing to embark on this method. In addition, they use this methodology to obtain two novel scientific findings. First, that incorporation of bulky ncAAs within the acidic pocket tend to accelerate current decay and lower apparent affinity of protons (Figure 3). Second, they provide evidence that PcTx1 still binds the previously published F352L mutation but just fails to modulate it. I have one principal criticism which should be addressed and a number of minor ones, all aimed at improving this report.

The capability of APC to probe changes in SSD (steady-state desensitization) is mentioned a few other times in the manuscript (lines 37, 92, 267, 422) yet the present data do not quite support that assertion. For example, line 265: "despite the reduced control over the conditioning pH compared to using a perfusion system with continuous flow, it was still possible to determine if BigDyn modulates hASIC1a SSD". But just a few lines above this, around line 260: "BigDyn increased rescue from pH-induced SSD in WT and all tested AzF-containing variants, although the effect was only significant for E355AzF and K356AzF (Figure 4B)". One cannot claim to have detected or evaluated an effect which is not statistically significant. I suggest the authors simply repeat the run one or two more times, to get another 3-6 data points per condition and then re-analyze the data. Also, the authors use a t-test with Welch's correction for unequal variance in this case (line 282). This is appropriate given the difference in variance (Figure 4B) but still assumes a normal distribution of samples. A better test would be a Mann Whitney U test which assumes neither normal distribution nor equal variance.
We thank the reviewer for alerting us to this shortcoming and for suggesting a different statistical analysis. We have repeated both the SSD recordings and the BigDyn modulation assay to be able to report data from a higher number of cells (Figures 4, S10, S11; Tables S4 and S5). We now use the suggested Mann Whitney test instead of the t-test with Welch's correction and report updated significance values. Accordingly, we have changed the corresponding sentence in the manuscript to:

"BigDyn increased rescue from pH-induced SSD in all tested AzF-containing variants, but did not do so in WT, despite a similar trend (Figure 4B)."

Minor

Why was GFP Y151TAG used for Azf but Y40TAG used for the others? (line 105)

During initial construct testing using microscopy, we observed qualitatively brighter fluorescence for GFP Y40TAG compared to Y151TAG when using Bpa and Se-AbK. For AzF, there was a substantial degree of unspecific incorporation in the Y40TAG construct and fluorescence was bright enough in the Y151TAG variant, so we chose the latter for AzF incorporation. We have now specified this in the text:

"To this end, we co-transfected 103 different hASIC1a variants containing individual TAG stop codons throughout the protein together with the suppressor tRNA/ncAA-RS pair for either AzF, Bpa or Se-AbK and a GFP-reporter carrying a TAG at Y40 (for Bpa and Se-AbK) or Y151 (for AzF, as we observed a higher degree of unspecific incorporation in the Y40TAG variant with AzF) into custom-made ASIC1a-KO HEK 293T cells."

The authors defined a site as having 'specific incorporation' if the peak current with ncAA > 1nA and without ncAA was < 500 pA (line 136). Unspecific incorporation was defined as currents which exceeded 1 nA in both cases. What about the case where currents were > 1 nA with ncAA but between 500 and 1000 pA without ncAA?

We agree that the sentence was potentially misleading. We defined incorporation as unspecific if currents in cells grown in the absence of ncAA exceeded 500 pA. We have changed the wording in the manuscript accordingly:

"If currents >500 pA were observed in the absence of ncAA, incorporation was considered unspecific (circles with lighter colour shade), while positions labelled in grey did not yield substantial currents in either condition (<1 nA)."

Also, rooting the definition in current magnitude ignores potential changes in open probability or conductance produced by the ncAA (ie. 500 pA of current when Popen has been reduced by 80% is still a lot of channels with specific incorporation). The authors should include this caveat in their definition and consider defining it initially as apparent specific incorporation or something like that.

We concur that by evaluating ncAA incorporation by current magnitude, we did not consider possible changes in open probability or conductance, primarily because our strategy focused on selecting variants with robust incorporation and WT-like function. However, we have now added a sentence to point out the caveat that we might be excluding variants with specific incorporation resulting in reduced Popen or conductance.

"On the other hand, by defining incorporation as not successful for currents <1 nA, we are aware that we may have potentially excluded variants in which specific ncAA incorporation resulted in reduced open probability or lower conductance."

Regarding the unspecific incorporation control (ie current in the absence of ncAA), the authors did not use FACS in this case because the number of GFP+ cells in the absence of ncAA is so low that downstream APC is not practical (lines 142, 489-495). Rather they just recorded a large number of transfected but unsorted cells (containing cells which were successfully transfected and cells which

were not). The authors should consider in future experiments beyond this paper to transfect wild type GFP for these controls followed by FACS. At least this way they are recording from cells which are more likely to contain all plasmids while dumping those that are less likely to have been successfully transfected with all components. This might give a better estimate of unspecific incorporation.
We thank the reviewer for this helpful remark and will implement this strategy in future experiments.

Figure 2E, how was relative incorporation calculated? Compared to currents in wild-type? This should be made clear.
We have now clarified in the text and in the legend for Figure 2E that relative ncAA incorporation was calculated by dividing the number of positions successfully replaced with an ncAA by the total number of positions at which incorporation was attempted.

Lines 216-218 and Figure 3B, the authors note that certain mutants in the acidic pocket accelerate current decay. This is also reported in the abstract and a point in the discussion however no numbers are given nor statistical test performed. I appreciate the heterogeneity of the time course (line 403) but some quantification is needed to support these statements beyond the clear difference in APC traces.
We thank the reviewer for suggesting to quantify this phenomenon, which we now address by calculating the t½ of current decay for the variants shown in Figure 3B compared to WT. We have added a bar graph in Figure 3B and refer to the new Table S3 for quantification and statistical analysis in the legend and in the manuscript text:
"For example, current decay rates were increased for T239Bpa (t½ 224 ± 176 ms, n=6) and D357AzF (t½ 93.8 ± 40.9 ms, n=10) compared to WT (t½ 818 ± 750 ms, n=9), indicating possible effects of the photocrosslinkers on channel gating (rates of desensitization or closure, Figure 3B and Table S3)."

Line 287, the authors mention past reports of PcTx1 modulating the channel in a pH-dependent way. Please consider citing PMID:16284080 where the binding of radio-labelled PcTx1 is measured over a range of pHs.
We thank the reviewer for alerting us to this relevant publication, which we now cite.

Lines 480-482, on the use of different expression plasmids, Rook et al., 2020 also used a plasmid with both tRNA and RS, as well as a C terminal GFP on the protein of interest. Please consider mentioning this and/or discussing.
We thank the reviewer for pointing this out and have added the strategies employed by Rook et al. to the discussion on the use of optimized expression plasmids:
"For example, the Plested group achieved co-expression of TAG-containing AMPAR and GFP with an internal ribosome entry site (IRES) [5, 7], while Rook et al. used ASIC1a with a C-terminally fused GFP-tag [8]. Furthermore, both Zhu and co-workers and Rook et al. created bidirectional plasmids to encode both AzF-RS or Bpa-RS and tRNA, respectively [6, 8]."

Lines 497-505, the authors discuss their use of solution exchange and stacking to measure activation and SSD in ASICs. They may wish to note that ASICs are an exceptionally challenging case to apply solution switching with APC, not only because so many processes are pH-dependent but because

they are so steeply pH-dependent (Hill coeffs 2-10). Most other ligand-gated channels have Hill co-effs around 1 for many of their processes. Using this HTS approach with 'better behaved' channels ought to be much easier!
We agree with the reviewer that ASICs are an exceptionally challenging case in terms of solution exchange. However, while our new proof-of-concept data on the P2X2 and GluA2 receptors show that the method is generally applicable to other ion channels, it is apparent that solution exchange needs to be optimized also for channels with lower Hill co-efficient (please see results section, Figure S7 and Table S2). But we would also like to point out that recent application notes by APC providers/users have indicated significant progress in this matter, especially regarding ligand dispension speed.

Line 551, why was the ncAA used in 'acid' form for biochemistry but methyl ester for APC?
For the more sensitive APC recordings, we used the ncAA methyl esters, allowing us to use lower concentrations of ncAA and profit from improved cell viability for patch-clamp recordings. For the biochemistry experiments, which require significantly more material for visualization, we resorted to the higher concentration of the 'acid' form to increase protein amounts. We have added a sentence in the method section to clarify this.

Line 590, please specify the timing of conditioning pHs used i. e. how long to equilibrate before activating pH, duration of interval between stimuli etc.
We thank the reviewer for alerting us to these missing details and have specified the timing of stimuli for the different APC protocols in the method section.

Line 612, was filtering really done at 45 Hz? Was it necessary to do this?
We can confirm we filtered the traces at 45 Hz, which is a standard option in the data control software of the APC platform. This was mainly done to remove solution artefacts arising from addition and removal of the pipette tips from the well. We have now specified this in the method section:
"Current traces were acquired at 2 kHz and filtered in the DataControl384 software using a Butterworth 4th order low pass filter at 45 Hz to remove solution artefacts."

Line 621 versus 627, authors use Hill coefficients in the text but Hillslope in the equation. May wish to keep it consistent.
We thank the reviewer for noticing this and have changed the wording to Hill slope throughout the text.

Line 637, please provide more detail on UV illumination. What was the source(s)? Intensity, distance from source, on ice, etc.
We thank the reviewer for alerting us to this shortcoming and have now added more details regarding the UV illumination used for crosslinking.

Reviewer #3, Haoxing Xu: This is an important technical advance that probed the structural-functional relationship on the acid-activated cation channel, ASIC1, using a high-throughput protocol and incorporation of non-canonical amino acids (ncAAs). Consistent with previous studies,

it was found that the acid pocket domain was crucial for proton activation and channel-toxin interaction. However, the study fell in short in revealing the global trends and patterns that regulate site-specific proton activation and toxin modulation. Although three photo-crosslinkers were used, no obvious differences in terms of channel electrophysiology were found between them at most sites/positions. Hence, the molecular mechanisms underlying proton activation, channel desensitization/ tachyphylaxis, and toxin binding/modulation have remained unclear. There are some interesting observations in the study. For instance, the effects of Pctx1 on the D357AzF variant, in terms of proton sensitivity and channel desensitization, were very interesting, but the data were somehow buried in the presentation. It would be more informative if the authors could perform more detailed studies on the D357 position using photocrosslinkers that would introduce amino acid residues with very distinct properties. The three photocrosslinkers used in the study all introduce bulky groups in the sites whose actions are presumed to be charged in nature. So it was not surprising that the information revealed was somewhat limited. Overall, it is an impressive technical breakthrough, which would yield more insights into channel physiology with thoughtful experimental design.

We thank the reviewer for his comments and agree that the effects of PcTx1 on the D357AzF variant are of particular interest. We have now added new experimental data at lower conditioning pH (pH 6.8-6.5), as well as data on Bpa-containing channels, to provide a more comprehensive analysis of D357 variants. In these experiments we observe a similar overall trend as compared to the other AzF variants tested: low proton concentrations during conditioning favor potentiation, while high proton concentrations during conditioning lead to inhibition (see Figure 4D). What is notable for this mutant is the exceptionally steep pH dependence and the degree of potentiation, both of which are more pronounced than in the other AzF-containing variants. For the corresponding D357Bpa variant, we observe a somewhat similar pattern with strong potentiation at a conditioning pH of 7.4, although inhibition already occurs at pH 7.0 (see new Figure S13). This suggests that the different sizes of the crosslinkers have an effect on both the pH dependence and the extent of toxin modulation.

Regarding the more general concern about limited biological insight, we would like to thank the reviewer for pointing out the "impressive technical breakthrough" – which in fact was the primary objective of this work. This aspect is also elaborated on in our reply to comment 3) of reviewer #4, see below.

New text in results section (page 16):

"We therefore chose to evaluate PcTx1 modulation of D357AzF in more detail. Specifically, we used lower pH during conditioning and observed that at pH 6.8 and below, the variant is inhibited. In light of the strong potentiation at pH 7.0, this highlights that PcTx1 modulation of D357AzF exhibits a striking pH dependence, which far exceeds that of WT and the other mutants examined here (Figure 4D, Table S6) [39]."

New text in results section (page 19):

"We therefore tested PcTx1 modulation of selected Bpa variants on the SyncroPatch 384PE to assess if the toxin binds to the acidic pocket when Bpa is present (Figure S13, Table S6). We observed robust inhibition at pH 7.0, indicating the interaction persists despite incorporating a bulky ncAA within the acidic pocket. However, most of the variants showed only weak modulation at the pH used during the UV exposure (7.4), which might partly explain the lack of crosslinking with Bpa."

Reviewer #4: In this manuscript, Braun and colleagues generated 309 variants of acid-sensing ion channel 1a that contained one of three different unnatural amino acids (or non-canonical amino acids; ncAAs). Channel variants were functionally characterized via automated patch clamp (APC) and several informative mutants were further characterized by photocrosslinking via the ncAAs. Generating such a vast amount of individual variants, functionally characterizing them with high-throughput methodology and selected variants also by state-of-the-art biochemistry represents a tour-de-force of ion channel structure-function analysis. In addition, the experiments were carefully performed and results have been well documented. As such, the work clearly "demonstrates a high standard of scientific rigor in its methodology and reporting" as required for publication in PLOS Biology. My main concern rests with the importance of the study for the field.

Main comments:
1) The main aim of the study was to "establish a high-throughput protocol to conduct functional and pharmacological investigations of ncAA-containing" ion channel variants (abstract, page 2). So far, this has been limited by "a combination of low efficiency of transient transfection and limited ncAA incorporation rates" (Introduction, page 3). The principal solution to this problem was enriching by FACS the population of transiently transfected cells expressing ncAA-containing channels. Although this was a clever idea, it is not really innovative and original and does not represent a major break-through per se.
We agree with the reviewer that the principal solution to achieve our goal was to combine two existing technologies. However, the same is true for a number of other technologies, such as single molecule FRET, Hydrogen Deuterium Exchange-Mass Spectrometry (HDX-MS) or patch-clamp fluorometry, all of which resulted from a merger of existing techniques. The key is that each of these approaches allowed to tackle questions that were previously difficult to address or even inaccessible. And while we do not claim our present work will have the same impact as some of the above examples, we still think it constitutes a valuable tool, as also pointed out by the other reviewers.

2) Related to my first comment, in my opinion, high-throughput analysis of ion channel variants is currently not limited mainly by a lack of appropriate protocols, but rather by the fact that APC platforms are not widely used, in particular in academic environments. I suppose this is mainly due to relatively high investment and running costs. The relative scarcity of APC platforms in academia will further limit the impact of this work.
As a side-note: since one of the authors (SF) is affiliated with the company that sells the APC platform, which was used in this study, I was wondering whether there is really no conflict of interest to declare.
We thank the reviewer for this comment and have added a section on accessibility of the method to better describe the impact of the study:
"Lastly, limitations arise from the accessibility and running costs of APC platforms compared to conventional patch-clamp set ups. But we anticipate that the establishment of academic core facilities for high-throughput electrophysiology (e.g. Northwestern University, Il, US; University of Nantes, France; Illawarra Health and Medical Research Institute, Wollongong, Australia) and collaborations between academia and industry (this study, [73-76]) will likely contribute to a

broader accessibility. This is also evident from the rising number of publications involving APC (currently >80 publications according to vendor information). "

Also, we would like to point out that issues with accessibility and cost are by no means rare. It similarly applies to a number of other widely used methods, such as cryo-EM microscopes, high-resolution NMR or MS facilities etc.

Finally, we fully agree that SFs affiliation with Nanion Technology should have been made more transparent. We apologize for this oversight and have added this to the conflict of interest section.

3) Given that establishing a high-throughput protocol per se is not sufficient to justify publication in PLOS Biology, a journal that "features works of exceptional significance, originality, and relevance", the authors need to show that their methodology can be used to perform such works. Although they used the ASIC1a variants to analyze the interaction of ASIC1a with the opioid peptide big dynorphin and the spider toxin PcTx1, highlighting that the approach is suitable for high-throughput analysis of ion channels variants, no really new insights in the interaction of ASIC1a with big dynorphin or PcTx1 have been obtained.

One can certainly argue whether the combination of a new protocol, which is not of exceptional originality, with a very carefully executed functional and pharmacological analysis, which does not yield new insights of exceptional relevance, is still sufficient to justify publication in PLOS Biology. In my opinion, however, the lack of new insights into ASIC function and pharmacology substantially weakens the impact of this study.

We would like to point out that the primary purpose of our work was to establish a novel methodology that would expand the scope and possibilities of studying ncAA-containing ion channels (see also 1) above). Further, we do think our data sheds new light on ASIC function and pharmacology: firstly, and as also pointed out by reviewer #2, we provide exhaustive evidence for bulky ncAAs causing accelerated current decay and lower apparent affinity of protons and show that PcTx1 still binds to the previously published F352L mutation, but just fails to modulate it. Secondly, our new data expand on these findings by demonstrating that differences in ncAA size at for example position D357 can have drastic consequences for the pH dependence and extent of potentiation by PcTx1.

Minor comments:

1) Page 6: The authors mention that they "cannot exclude the possibility of underestimating the degree of unspecific incorporation". Isn´t it actually likely that they underestimated the degree of unspecific incorporation if enriching of transfected cells grown in the absence of ncAAs by FACS was not feasible? Please comment.

We agree with the reviewer that the employed strategy is not the ideal solution for assessing unspecific incorporation. We are now more transparent about this issue by evaluating the success rate of this strategy through comparison of the number of tested cells with and without current for different positions (please see new Figure S1C). We conclude that despite its shortcomings, the approach allows us to detect unspecific incorporation when it occurs in >15% of cells (please also see comment 3) by reviewer #1).

2) Page 6: the authors state that they "observed mostly robust incorporation". As I understand from the next sentence, incorporation was observed at 44%-61% of the 80 positions tested. This is roughly 50%, but not "mostly".
We thank the reviewer for pointing this out and have removed the word "mostly" from the sentence.

3) At several places, the authors mention the limitation of the APC system in controlling the conditioning pH, which represents a major drawback for an ion channel like an ASIC. For a reader, who is not familiar with the APC system used, it is difficult to grasp the technical problems and potential limitations. Is it possible to explain this in more detail, perhaps in the Methods or the supplement?
We thank the reviewer for this suggestion and have explained the technical limitations for changing the conditioning pH in more detail in the method section and in the new Figure S9. In brief, the cell is patched at the bottom of an open well, with liquid addition being performed from above. This means that in order to change the conditioning pH, not all the liquid around the cell can be exchanged at once, as that would result in losing the cell. We therefore added new liquid stepwise by exchanging 50% of the solution in the well 2-3 times to approximate the target pH. Notably, there are alternative APC instruments on the market that utilize microfluidic flow channel systems that might be better suited to control the conditioning pH for SSD recordings.

4) The authors show that the F352L mutation still binds PcTx1 (Fig. 5C). But since the Western blot signal was greatly diminished, I think the authors cannot conclude that F352L "selectively abolishes the functional affects caused by PcTx1" (page 17).
We would like to point out that we do consider the observation that the F352L mutation does not eliminate PcTx1 binding quite notable because the concentration of PcTx1 used in this experiment (100 nM) is very low compared to the toxin concentrations required to functionally inhibit this mutant (>1 μM).
However, to account for the concern of the reviewer, we have altered the sentence in question to: "…but likely primarily abolishes the functional effects caused by PcTx1."

5) "ASIC1a*PcTx1" and "Endogenous carboxylases" should be clearly marked by arrows in Fig. 5C.
We thank the reviewer for this comment and have added arrows to Figure 5C and Figure S12 to more clearly mark the bands.

6) On page 21, the authors speculate that the introduction of the bulkier Bpa partially occludes the acidic pocket and thus hinders binding of PcTx1. In principle, the authors could test experimentally their speculation.
We agree with the reviewer that this speculation merits additional work. We have therefore assessed a selection of variants containing Bpa in the acidic pocket on the SyncroPatch to determine if PcTx1 still modulates these variants (new Figure S13, Table S6). The toxin inhibits all variants at pH 7.0 and should therefore still interact with the acidic pocket despite the presence of Bpa. This points towards steric constraints (orientation of Bpa towards PcTx1, reactivity of Bpa towards C-H bonds only) as the underlying cause for the lack of crosslinking. Interestingly, at pH 7.4 (the pH we used for our crosslinking attempts followed by Western blots), we do not see modulation for several of the

variants. It could therefore be interesting to assess potential differences in crosslinking patterns at different pH in future studies.

New text in results section (page 19):
"Attempts to photocrosslink PcTx1 using Bpa in the equivalent positions around the acidic pocket did not succeed (Figure S12). We therefore tested PcTx1 modulation of selected Bpa variants on the SyncroPatch 384PE to assess if the toxin binds to the acidic pocket when Bpa is present (Figure S13, Table S6). We observed robust inhibition at pH 7.0, indicating the interaction persists despite incorporating a bulky ncAA within the acidic pocket. However, most of the variants showed only weak modulation at the pH used during the UV exposure (7.4), which might partly explain the lack of crosslinking with Bpa."

New text in discussion (page 23):
"We note that unlike AzF, we were unable to employ Bpa for crosslinking experiments with PcTx1. To test if introduction of the more bulky photocrosslinker prevents toxin interaction, we assessed PcTx1 modulation of selected variants with APC and found robust inhibition for most variants, indicating that Bpa does not fully occlude the acidic pocket. We therefore speculate that steric constraints due to the positioning of the benzophenone diradical and the more selective reactivity of Bpa (reacts exclusively with C-H bonds) may play a role [70, 71]."
